# Definition of novel cell envelope associated proteins in Triton X-114 extracts of *Mycobacterium tuberculosis *H37Rv

**DOI:** 10.1186/1471-2180-10-132

**Published:** 2010-04-29

**Authors:** Hiwa Målen, Sharad Pathak, Tina Søfteland, Gustavo A de Souza, Harald G Wiker

**Affiliations:** 1 Section for Microbiology and Immunology, the Gade Institute, University of Bergen, Bergen, Norway; 2 Department of Microbiology and Immunology, Haukeland University Hospital, Bergen, Norway

## Abstract

**Background:**

Membrane- and membrane-associated proteins are important for the pathogenicity of bacteria. We have analysed the content of these proteins in virulent *Mycobacterium tuberculosis *H37Rv using Triton X-114 detergent-phase separation for extraction of lipophilic proteins, followed by their identification with high resolution mass spectrometry.

**Results:**

In total, 1417 different proteins were identified. *In silico *analysis of the identified proteins revealed that 248 proteins had at least one predicted trans-membrane region. Also, 64 of the identified proteins were predicted lipoproteins, and 54 proteins were predicted as outer membrane proteins. Three-hundred-and-ninety-five of the observed proteins, including 91 integral membrane proteins were described for the first time. Comparison of abundance levels of the identified proteins was performed using the exponentially modified protein abundance index (emPAI) which takes into account the number of the observable peptides to the number of experimentally observed peptide ions for a given protein. The outcome showed that among the membrane-and membrane-associated proteins several proteins are present with high relative abundance. Further, a close examination of the lipoprotein LpqG (Rv3623) which is only detected in the membrane fractions of *M. tuberculosis *but not in *M. bovis*, revealed that the homologous gene in *M. bovis *lack the signal peptide and lipobox motif, suggesting impaired export to the membrane.

**Conclusions:**

Altogether, we have identified a substantial proportion of membrane- and membrane-associated proteins of *M. tuberculosis *H37Rv, compared the relative abundance of the identified proteins and also revealed subtle differences between the different members of the *M. tuberculosis *complex.

## Background

Tuberculosis is an airborne infection caused by *Mycobacterium tuberculosis*. It is estimated that one-third of the world's population is latently infected with *M. tuberculosis*, and that each year about three million people die of this disease. The emergence of drug-resistant stains is further escalating the threat to public health (WHO, 2003). In spite of global research efforts, mechanisms underlying pathogenesis, virulence and persistence of *M. tuberculosis *infection remain poorly understood [1].

*M. tuberculosis *is a facultative intracellular pathogen that resides within the host macrophages [2-4]. When *M. tuberculosis *invades host cells, the interface between the host and the pathogen includes membrane- and surface proteins likely to be involved in intracellular multiplication and the bacterial response to host microbicidal processes [4]. Recently, the cell wall of *M. tuberculosis *was reported to posses a true outer membrane adding more complexity with regard to bacterial-host interactions and also important information relevant for susceptibility to anti-mycobacterial therapies [5-7]. Revealing the composition of the membrane proteome will have an impact on the design and interpretation of experiments aimed at elucidating the translocation pathways for nutrients, lipids, proteins, and anti-mycobacterial drugs across the cell envelope. According to bioinformatic predictions, 597 genes (~15%) of the *M. tuberculosis *H37Rv genome [8,9], could encode proteins having between 1 and 18 transmembrane α-helical domains (TMH), which interact with the hydrophobic core of the lipid bilayer. The confirmation of the expression of these genes at the protein level may lead to new therapeutic targets, new vaccine candidates and better serodiagnostic methods.

Membrane proteins resolve poorly in two-dimensional polyacrylamide gel electrophoresis (2D-PAGE) and proteomic profiling of mycobacterial membrane proteins remains a major challenge. Their limited solubility in aqueous buffer systems and their relatively low abundance in a background of highly abundant cytoplasmic proteins have yet to be overcome. Several studies have reported extraction of membrane- and membrane-associated proteins using centrifugation to obtain purified cell wall and cell membrane fractions for analysis by sodium-dodecyl-sulphate polyacrylamide gel electrophoresis (SDS-PAGE) in combination with liquid chromatography tandem mass spectrometry (LC-MS/MS) [10-13]. Common for these studies is pre-isolation of the membrane and cell wall of the bacteria, and application of different washing techniques prior to protein extraction by detergents. In this study, we separated hydrophobic membrane- and membrane-associated proteins directly from sonicated *M. tuberculosis *H37Rv using phase separation with Triton X-114. The efficacy of this method was shown with *Mycobacterium bovis *BCG in a previous work [14].

Comparison of expressed levels of the identified proteins was performed using the emPAI [15,16] This approach relates the number of experimentally observed peptide ions in a given protein to the number of theoretically observable peptides. Our results show that among the membrane-and membrane-associated proteins several proteins are present in high relative abundance. Using bioinformatic analysis, we also found that the gene sequence encoding Rv3623 which is annotated as a potential lipoprotein in both *M. tuberculosis *and *M. bovis*, is shorter in *M. bovis *and have lost the N-terminal signal peptide and lipobox that mediate the prelipoprotein translocation and its subsequent lipidation that retains it to the membrane.

## Results

### Identification of Triton X-114 extracted proteins

**Figure 1:**
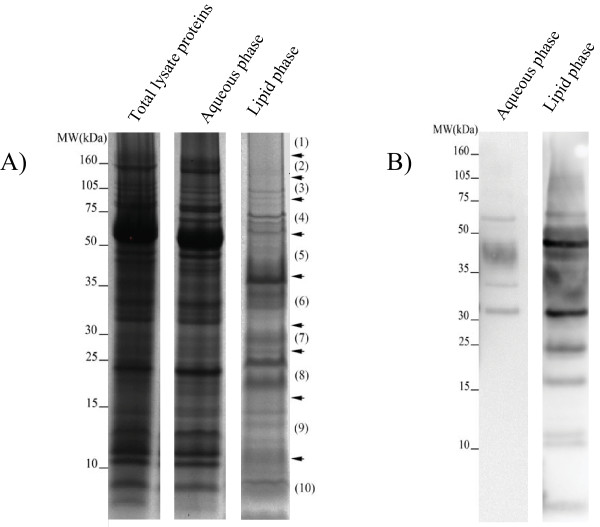
**SDS-PAGE analysis of the extracted *M. tuberculosis *H37Rv proteins**. Panel A, shows the whole cell lysate of *M. tuberculosis *H37Rv, the aqueous phase proteins and the lipid phase proteins after Triton X-114 extraction. The fractions for LC-MS/MS analysis of the lipid phase is indicated. Explanation of the fraction numbers: (1) >160 kDa, (2) 105-160 kDa, (3) 75-105 kDa, (4) 50-75 kDa, (5) 35-50 kDa, (6) 30-35 kDa, (7) 25-30 kDa, (8) 15-25 kDa, (9) 15-10 kDa, (10) <10 kDa. Panel B shows western blot analysis of the aqueous and lipid phases using a polyclonal rabbit antiserum against a BCG cell wall fraction. The molecular weight standards are shown on the left hand side of each panel.

The aim of this study was to enrich and perform a comprehensive proteomic analysis of membrane- and membrane-associated proteins of the virulent reference strain *M. tuberculosis *H37Rv. For this purpose, the hydrophobic proteins were enriched by lysing whole bacilli followed by phase separation with the Triton X-114 detergent. After phase separation, the proteins in the lipid phase were precipitated by acetone and separated by SDS-PAGE. As shown in Figure 1 panel A, the lipid phase was quite complex, but appeared to be enriched for certain proteins as compared to the unfractionated crude lysate. In a parallel experiment, and to validate that the protein content in the lipid and aqueous phases were different, proteins from both phases were separated and transferred to nitrocellulose membranes which were developed with polyclonal antibodies against a cell wall fraction of *M. bovis *BCG (Figure 1, panel B). Notably, Figure 1 not only demonstrates that the protein content of the aqueous phase and the lipid phase was different, but also clearly shows that the lipid phase was indeed enriched for cell wall proteins. In order to identify the proteins of the Triton X-114 detergent fraction, the protein mixture was separated with SDS-PAGE (Figure 1A), run in duplicate and cut into ten pieces each (twenty fractions in total) and subjected to in-gel digestion by trypsin. The resulting peptides were eluted and analysed by high accuracy mass spectrometry. Additional file 1, Figure S1 illustrates the sequence obtained for ion m/z 1210.62 which was identified by Mascot as peptide CGSPAWDLPTVFGPIAITYNIK from protein Rv0932c with a Mascot score of 79. Such fragmentation data contain a very good coverage of the expected y- and b-series daughter ions plus the presence of other ions which indicates the correct MS/MS assignment such as two highly abundant y-ions of proline (y19++ and y14). This is very typical for peptides containing proline.

**Figure 2:**
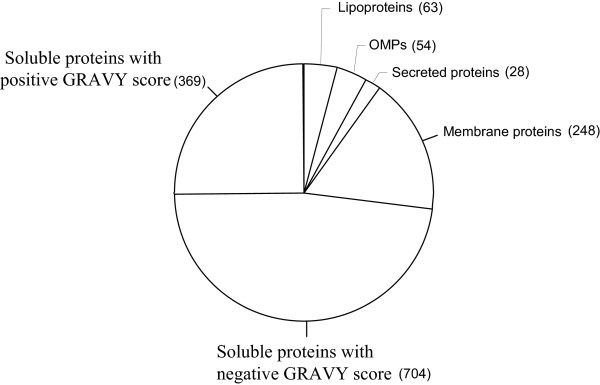
**Number of proteins within main functional categories identified in the Triton X-114 detergent phase prepared from *M. tuberculosis *H37Rv**.

In total, 1417 proteins extracted with Triton X-114 were identified from the *M. tuberculosis *H37Rv strain out of which 395 are described for the first time. The complete lists of proteins with identified peptides are provided as additional data files (Additional file 2, Table S1 and Additional file 3, Table S2). Information about the criteria for protein identifications, such as number of peptides matching each protein, scores, identification threshold and peak lists are given in Additional file 4, Table S3. Identified proteins were categorized according to functional classification (Table 1). An overview of the number of observed proteins belonging to major groups based on physicochemical properties is shown in Figure 2. These groups are described below:

**Table 1:** Functional classification of the identified *M. tuberculosis *H37Rv proteins.

*Functional group^a^*	***Functional group no***.	*Total protein number^b^*	*Number of observed proteins^c^*
Virulence, detoxification, adaptation	0	212	44 (21%)
Lipid metabolism	1	237	84 (35%)
Information pathways	2	232	98 (42%)
Cell wall and cell processes	3	751	313 (42%)
Stable RNAs	4	50	0 (0%)
Insertion sequences and phages	5	147	0 (0%)
PE/PPE	6	168	14 (8%)
Intermediary metabolism and respiration	7	898	412 (46%)
Unknown	8	15	0 (0%)
Regulatory proteins	9	194	54 (28%)
Conserved hypotheticals	10	895	299 (33%)
Conserved hypotheticals with an orthologue in *M. bovis*	16	262	52 (20%)

^a ^The functional groups were taken from the Tuberculist database, publically available at http://genolist.pasteur.fr/TubercuList/.

^b ^Total number of proteins in each group predicted in the genome.

^c ^Number of proteins identified and the ratio compared to the total number of proteins assigned to each functional group.

#### Membrane proteins

**Figure 3:**
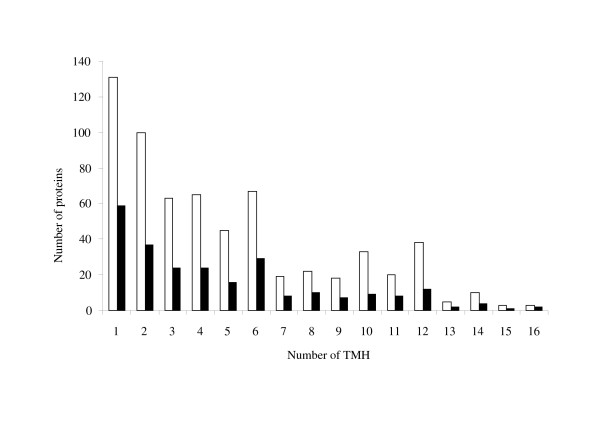
**Number of TMH regions in membrane proteins identified in the Triton X-114 lipid phase fraction of *M. tuberculosis *H37Rv**. Number of identified proteins compared to the total number of predicted proteins is given. The white bars represent the total number of predicted membrane proteins in the genome based on the TMHMM algorithm version 2.0, while the black bars represent those observed in the present study.

According to TMHMM version 2.0, a bioinformatic algorithm that predict transmembrane regions in the primary amino acid sequences, 597 genes (~15%) of the *M. tuberculosis *H37Rv genome were found to possess between 1 and 18 TMHs. Each α-helix consists of 10 to 15 amino acid residues which interact with the hydrophobic core of the lipid bilayer. The proteins identified in this study were analysed by the TMHMM algorithm and 248 were predicted to have 1 or more TMH regions (Figure 3), among those, 90 represented novel identifications (Additional file 2, Table S1). Proteins with one TMH were only considered as possible membrane proteins if the TMH region was positioned beyond the first 70 N-terminal amino acids. This was done to avoid confusion with potential secreted proteins.

#### Lipoproteins

Lipoproteins represent a subgroup of exported proteins characterized by the presence of a lipobox. The lipobox motif is located in the distal C-terminal part of the N-terminal signal peptide [17]. This motif is a recognition signal for lipid modification on the conserved and essential cysteine residue. Precursor lipoproteins are mainly translocated in a Sec-dependent manner across the plasma membrane and are subsequently modified [18]. The proteins identified in this study were analysed by the lipoP algorithm http://www.cbs.dtu.dk/services/LipoP/, and 63 were predicted as potential lipoproteins (Additional file 2, Table S1) based on the presence of a cleavable signal peptide and a lipobox motif. Eight lipoproteins are described for the first time. In sum the findings comprises over 56% of all predicted lipoproteins in the genome.

#### Outer membrane proteins

Outer membrane proteins (OMPs) are a class of proteins residing in the outer membrane of bacterial cells. Identification of OMPs is important as they are exposed on the bacterial surface and so are accessible drug targets. Recently, Song and colleagues analysed the genome of *M. tuberculosis *and predicted 144 proteins as potential OMPs based on the amphilicity of the β-strand regions, absence of hydrophobic α-helices and the presence of a signal peptide [19]. In our study, we observed 54 (37.5%) of these proteins, and 9 of them have not been described in previous proteomic works (Additional file 2, Table S1).

#### GRAVY

The 'grand mean of hydropathicity' (GRAVY) score is the average hydropathy score for a protein. According to Kyte and Doolittle, integral membrane proteins have a higher GRAVY score than soluble proteins. A positive score >-0.4 suggests increased probability for membrane association; the higher the score, the greater the probability [20]. GRAVY scores were calculated for all the identified proteins using the PROTPARAM tool http://us.expasy.org/tools/protparam.html. Three-hundred and sixty nine proteins without a TMH region had positive GRAVY scores (Additional file 3, Table S2). A substantial proportion of the detected proteins lacked a predicted retention region and had a negative GRAVY score, suggesting that they were soluble proteins. However, it is possible that at least some of them might be functionally membrane-associated through formation of protein complexes with membrane-anchored proteins. In a previous study we showed that several hydrophilic proteins are retained in the lipophilic membrane fraction due to interaction with hydrophobic proteins [21-23].

#### Relative abundance index

To estimate the relative abundance of the observed proteins, we used the emPAI algorithm, which is based on the calculation of identified peptides per protein and normalized by the theoretical number of peptides for the same protein (PAI). The outcome of the emPAI analysis is given for a selection of membrane proteins and lipoproteins with the highest values in Table 2 and 3, respectively. At the top of the membrane protein list is the possible proline rich antigen *pra *(Rv1078), with 5.66 mol %. This is a small protein with 25 kDa, and has 2 TMHs. When digested with trypsin, it constitutes 6 observable tryptic peptides, where 5 of them were identified. This protein has also been observed in *M. bovis *[14,24]. The membrane proteins Rv1078 and Rv1489 are the most abundant ones, but with no annotated biological functions. In the lipoprotein list only the first three proteins are assigned functions, while the 7 others have unknown biological functions.

**Table 2:** List of the 14 most frequently observed membrane proteins.

*Sanger ID*	*Gene name*	*Protein identity*	*No. of TMH^a^*	*No. of observed peptides^b^*	*emPAI* *(Mol %)^c^*	*References*
Rv1078	*pra*	Possible proline rich antigen	2	5	5.66	[14,24]
Rv1489	-	Conserved hypothetical protein	2	5	1.30	[26]
Rv1306	*atpF*	Possible ATP synthase b chain	1	7	0.36	[14,24-26]
Rv2563	-	Possible glutamine-transport transmembrane protein	4	13	0.35	[14,25,26,32]
Rv1234	-	Possible transmembrane protein	2	7	0.26	[25,26]
Rv0072	-	Possible glutamine-transport transmembrane protein	4	11	0.23	[25,26]
Rv0479c	-	Possible conserved membrane protein	1	11	0.23	[24-26]
Rv2969c	-	Possible conserved membrane or secreted protein	1	11	0.19	[14,24-26,40]
Rv2200c	*ctaC*	Possible transmembrane cytochrome C oxidase	3	13	0.17	[14,24-26,32]
Rv2195	*qcrA*	Possible rieske iron-sulfur protein	3	15	0.16	[14,24-26,40,54]
Rv1223	*htrA*	Possible serine protease	1	19	0.15	[24,26,54]
Rv1822	-	Phosphatidylglycerophosphate synthase	4	5	0.14	[14]
Rv2721c	-	Possible conserved transmembrane protein	2	12	0.13	[14,24-26,32]
Rv3273	-	Possible transmembrane carbonic anhydrase	10	11	0.11	[24-26,54]

^a ^Number of TMH regions predicted by TMHMM version 2.0 publically available at http://www.cbs.dtu.dk/services/TMHMM/.

^b ^Number of observed unique peptides from each protein.

^c ^Relative protein abundance provided in mol % concentration.

**Table 3:** List of the 10 most frequently observed lipoproteins.

*Sanger ID*	*Gene name*	*Protein identity*	*No. of observed peptides^a^*	*emPAI* *(Mol %)^b^*	*References*
Rv0432	*sodC*	Possible periplasmic superoxide dismutase	6	2.36	[14,24-26,40]
Rv3763	*lpqH*	19 kda lipoprotein antigen precursor	3	1.05	[14,24-26,40,55]
Rv0932c	*pstS2*	Periplasmic phosphate-binding lipoprotein	9	0.39	[14,24-26,45]
Rv2945c	*lppX*	Possible conserved lipoprotein	6	0.21	[14,24-26,45,54]
Rv1411c	*lprG*	Possible conserved lipoprotein	6	0.19	[14,24-26,40,54]
Rv0928	*pstS3*	Periplasmic phosphate-binding lipoprotein	7	0.16	[14,24,26,45]
Rv0583c	*lpqN*	Probable conserved lipoprotein	3	0.12	[14,25,26,32]
Rv1275	*lprC*	Possible lipoprotein	6	0.12	[14,24,25,54]
Rv2116	*lppK*	Probable conserved lipoprotein	4	0.12	[14,25,26]
Rv3623	*lpqG*	Possible conserved lipoprotein	7	0.11	[25,26,40]

^a ^Number of observed unique peptides from each protein.

^b ^Relative protein abundance provided in mol % concentration.

### Gene sequence analysis

**Figure 4:**
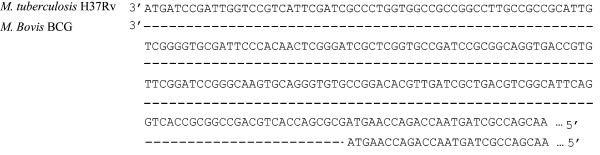
**Alignment of LpqG, "possible conserved lipoprotein" gene sequences from *M. tuberculosis *and *M. bovis***.

An in-depth analysis of our data indicated that 2 proteins were consistently identified in *M. tuberculosis *and not in *M. bovis *and these were: possible glutamine-transport transmembrane protein ATP binding cassette (ABC) transporter (Rv0072) and possible conserved lipoprotein LpqG (Rv3623). The DNA sequences encoding the two proteins including 100 base pairs (bp) up-stream were obtained from Tuberculist for *M. tuberculosis *and BoviList for *M. bovis *and the sequences were aligned using the Blast 2 algorithm. No differences were found for Rv0072 which had 100% similarity between *M. bovis *and *M. tuberculosis*. However, the conserved lipoprotein LpqG (Rv3623) appeared to be 207 bp shorter in *M. bovis *compared to *M. tuberculosis *with a difference in the N-terminal end of the gene. Consequently, the protein product was 69 amino acids shorter. When the primary sequence of the protein product was analysed by the LipoP algorithm, it appeared that the lipobox was missing in *M. bovis *and the protein cannot be considered as a lipoprotein (Figure 4).

## Discussion

Due to the anticipated role of membrane- and membrane-associated proteins of *M. tuberculosis *in virulence, it is important to characterize these proteins. Therefore, the aim of the present study was to perform a proteomic analysis of these proteins from the virulent reference strain *M. tuberculosis *H37Rv in extracts obtained with the non-ionic detergent Triton X-114. The proteins from the lipid phase of the detergent, which was enriched for membrane proteins as validated by immuno-blotting (Figure 1, panel B), were precipitated, separated, and identified by high accuracy mass spectrometry. In total, 1417 proteins were identified and analysis of the primary amino acid sequences by bioinformatic tools revealed that 31% of the proteins were membrane- or membrane-associated. The list included more than 50% of all predicted integral membrane proteins in the genome.

These results show a significant improvement compared to the two studies of mycobacterial plasma membrane proteins by Gu *et. al*. [25] and Xiong *et al*., [26]. In these studies, membrane proteins were enriched by differential centrifugation and alkaline treatment of crude membranes with sodium carbonate and urea and separated by SDS-PAGE followed by protein identification with LC-MS/MS. The study by Gu *et al*. revealed 739 *M. tuberculosis *H37Rv proteins including 85 membrane proteins (11.5%), while Xiong *et al*. identified 349 proteins, of which 100 were predicted membrane proteins (28.7%). The low percentage of integral plasma membrane proteins among the proteins identified in these studies was probably based in the membrane enrichment methods. We reduced the soluble protein contamination by phase separation of whole bacterial sonicates, and also applied state-of-the-art mass spectrometry analysis for identification of peptides.

More than 50% of all predicted lipoproteins in the genome were found. These are proteins translocated across the cell membrane and retained in the cell envelope by post-translational lipid modification. They are functionally diverse, and are suggested to be involved in host-pathogen interactions [27,28]. They are also of interest with respect to development of serodiagnostic tests for tuberculosis due to their strong immunogenicity [29,30].

We also found 37% of all predicted OMPs [19], which is an essential group of proteins involved in import of nutrients, secretion processes and host-pathogen interactions in gram-negative bacteria [31], and this is also likely to be of great importance in mycobacteria because it is now firmly established that they have a true outer membrane [5-7].

Even though a considerable number of observed proteins were predicted as integral membrane- or membrane-associated proteins, a substantial proportion of the detected proteins lacked a predicted retention region. For those proteins we measured the GRAVY score which express the total hydrophobicity of a protein as an indicator for membrane association. However, this is just a measure of increased probability for membrane association based on the fact that most integral membrane proteins have a positive GRAVY value. If a protein has a positive value, even though it lacks a retention signal, it is probably associated with the membrane. On the other hand, some of the hydrophilic proteins with a negative GRAVY value might still be retained in the membrane through formation of protein complexes with membrane-anchored proteins [21-23]. Several proteins in this group are encoded in operons of well known integral enzyme complexes [14].

Using state-of-the-art proteomic instrumentation and techniques, subtle details could be revealed at the individual protein level, such as experimental identification of signal peptide cleavage sites of predicted secreted proteins [32], or confirmation of the start codon, or identification of peptides from regions predicted to be non-coding thus indicating a more up-stream start codon [33,34], or even detection of novel genes [35]. Therefore, the data obtained in this study was examined both in detail and in the context of what have been reported in the literature. To examine the amounts of individual proteins in the membrane fraction we applied the emPAI algorithm. The emPAI calculation gives an approximate estimate of the abundance of a certain protein, and it calculates the protein concentration (in mol %) [15,16]. An advantage of this method is that it gives a more realistic picture of the protein profile compared to the mRNA levels, which could be difficult to relate to the actual protein amount. The membrane proteins (14 proteins) and the lipoproteins (10 proteins), with the highest relative abundance values are listed in Tables 2 and 3, respectively.

Interestingly, two of the proteins (Rv0072 and Rv2563) among those with the highest relative abundance values were "possible glutamine-transport transmembrane ABC transporter protein", with sequence motifs that belong to the ABC transport system. Glutamine is a major cell wall component of pathogenic mycobacteria only [36]. Its production is mainly catalyzed extracellulary by glutamine synthetase *GlnA1 *(Rv2220) [37]. Tullius *et. al*., 2003 showed that a *M*. *tuberculosis glnA1 *mutant requires a relatively high level of exogenous L-glutamine for growth *in vitro*, and the mutant was attenuated for intracellular growth in differentiated THP-1 cells, and it was also avirulent in infected guinea pigs [38]. Identification of two related proteins among the most abundant membrane proteins in *M. tuberculosis*, underlines the importance of production and transport of glutamine for the pathogen and its virulence.

The Rv0072 protein is only reported in studies conducted on *M. tuberculosis *[25,26] and not on *M. bovis *BCG (11, 17). It was identified by 11 different peptides giving sequence coverage of 44%, and the high emPAI value observed for this membrane protein suggests that it is abundantly present in the membrane of the virulent *M. tuberculosis *H37Rv strain. The open reading frames and sequences 100 bp up-stream to the start codon from *M. tuberculosis *H37Rv and *M. bovis *BCG 1173P2 and AF2122/97 were aligned, but the DNA sequences were identical and could not explain why Rv0072 has not been observed in *M. bovis *(data not shown).

Among the 10 most abundant lipoproteins 7 were not assigned any biological function, reflecting a fundamental lack of knowledge about these proteins. A careful examination revealed that the possible conserved lipoprotein LpqG (Rv3623) lies on the border of region of difference 9 (RD9) [39]. RD9 is deleted from all *M. bovis *lineages and consequently this protein has only been identified in proteomic studies performed on *M. tuberculosis *H37Rv [25,40], but not been reported in previous proteomic works on *M. bovis *BCG [14,24,41]. This RD region is also missing in other mycobacterial strains such as *Mycobacterium microti *or *Mycobacterium pinnipedii*. This region was first described by Gordon *et. al*., 1999 [42] as RD8 and later put in an evolutionary context by Brosch *et. al*., 2002 [43], which now corresponds to the region RD09 described by Behr *et. al*., [39]. A close examination of the gene encoding Rv3623 revealed that it is 207 bp shorter with a deletion in the N-terminal region that includes the signal peptide and the predicted lipo-box in the genomic sequences of *M. bovis *AF2122/97 and *M. bovis *BCG Pasteur 1173P2. The gene is annotated to encode a lipoprotein in the *M. bovis *strains even though the lipo-box is missing and it is therefore questionable whether it should be considered as a lipoprotein in *M. bovis*. The identification of this protein with 7 peptides covering 34% of its sequence in *M. tuberculosis *H37Rv suggests that it is a major lipoprotein.

The two lipoproteins listed in Table 3, annotated as "periplasmic phosphate-binding lipoprotein" (Rv0932c) is a known antigen [44] that also induces antibody responses in tuberculosis patients [45]. The 19 kDa lipoprotein antigen precursor (Rv3763) have been extensively studied due to its immunogenic properties [46-49]. Enrichment and analysis of lipoproteins with respect to humoral and cell-mediated immunity in infected individuals might ultimately lead to the identification of additional antigens that can serve as biomarkers for *M. tuberculosis *infection.

## Conclusion

In summary, we have enriched and extracted membrane- and membrane-associated proteins from *M. tuberculosis *H37Rv using Triton X-114, and identified the largest number of this subset of proteins reported so far. Further analysis of the data obtained in this study with bioinformatic tools suggests that several of these proteins are major membrane proteins. We have described one major lipoprotein of *M. tuberculosis *which has become a pseudogene by the RD9 deletion in *M. bovis*.

## Methods

### Preparation of crude bacterial extracts

The mycobacterial reference strain *M. tuberculosis *H37Rv (ATCC 27294), used in this study was kindly provided by Dr Harleen Grewal, The Gade Institute, University of Bergen, Bergen, Norway. The bacilli were cultured on Middelbrook 7H10 agar plates with OADC enrichment (BD Difco) at 37°C and 5% CO_2 _for 3-4 weeks. Bacterial colonies were harvested by using an extraction buffer consisting of phosphate-buffered saline (PBS), pH 7.4 with freshly added Roche Protease Inhibitor Cocktail (Complete, EDTA-free, Roche Gmbh, Germany). Six hundred μl of this extraction buffer was added to each agar plate and the mycobacterial colonies were gently scraped off the agar surface using a cell scraper. Aliquots of the resulting pasty bacterial mass was transferred into 2 ml cryo-tubes with O-rings (Sarstedt, Norway) containing 250 μl of acid washed glass beads (≤ 106 μm; Sigma-Aldrich, Norway) and an additional 600 μl of extraction buffer, and stored at -80°C until protein extraction was performed. For protein extraction, the mycobacteria were disrupted mechanically by bead-beating in a Ribolyser (Hybaid, UK) at max speed (6.5) for 45 seconds.

### Triton X-114 extraction of exported proteins from whole bacteria

Triton X-114 phase-separation was used to isolate lipophilic proteins following the method of Bordier [50]. In brief, 3-4 week old bacilli were lysed by bead beating and centrifuged, initially at 2300 *g *to remove unbroken cells and cell-wall debris. Triton X-114 was added to the supernatant (final detergent concentration 2%, v/v) and the suspension was stirred at 4°C for 20 minutes to obtain the protein extract in a single phase. Residual insoluble matter was removed by centrifugation at 15700 *g *for 10 min, and the solution separated into two phases, an upper (aqueous) and lower (detergent) phase after 10 minutes incubation at 37°C. The detergent phase was collected and proteins were precipitated by acetone.

### Gel electrophoresis and in-gel digestion of proteins

Extracted proteins (50 μg) were mixed with 25 μl SDS loading buffer and boiled for 5 minutes before separation on a 10 cm long 1 mm thick 12% SDS polyacrylamide gel (Invitrogen, Carlsbad, CA, U.S.A.). The protein migration was allowed to proceed until the bromophenol dye had migrated to the bottom of the gel. The protein bands were visualized with Coomassie Brilliant Blue R-250 staining (Invitrogen). Protein lanes were excised and divided in fractions according to the bands of the protein standard, ranging from ~3 kDa to ~188 kDa. The gel pieces were washed twice with 50% acetonitrile (ACN) in 25 mM ammonium bicarbonate (NH_4_HCO_3_) for 15 minutes at room temperature (RT), and subsequently dehydrated by incubating them with 50 μl 100% ACN for 20 minutes at RT. The proteins were reduced using 10 mM dithiotreitol and alkylated with 55 mM iodoacetamide; both in 100 mM NH_4_HCO_3_. The gel pieces were dehydrated by 100% ACN as described above, and rehydrated in 25 mmol/l NH_4_HCO_3 _followed by in-gel protein digestion with trypsin (Promega, Madison, U.S.A.) for 16-20 h at 37°C. The digested peptides were eluted by incubating the gel pieces with 50 μl 1% formic acid (FA) for 20 minutes at RT. The supernatant containing the peptides were collected after centrifugation at 15700 *g *for 10 minutes. Then, the gel pieces were incubated with 50 μl 0.1% FA in 50% ACN for 20 minutes at RT, followed by centrifugation at 15700 *g*. The supernatant was collected and combined with the previous one. Finally, the gel pieces were dehydrated with 50 μl 100% ACN for 20 minutes at RT, and the supernatant was collected after centrifugation as described above and added to the pool.

### Mass spectrometry

Experiments were performed on a Dionex Ultimate 3000 nano-LC system (Sunnyvale CA, USA) connected to a linear quadrupole ion trap-Orbitrap (LTQ-Orbitrap) mass spectrometer (Thermo Electron, Bremen, Germany) equipped with a nanoelectrospray ion source. The mass spectrometer was operated in the data-dependent mode to automatically switch between Orbitrap-MS and LTQ-MS/MS acquisition. Survey full scan MS spectra (from m/z 400 to 2,000) were acquired in the Orbitrap with resolution R = 60,000 at m/z 400 (after accumulation to a target of 1,000,000 charges in the LTQ). The method allowed sequential isolation of up to five of the most intense ions for fragmentation on the linear ion trap using collision induced dissociation at a target value of 100,000 charges.

For accurate mass measurements the lock mass option was enabled in MS mode and the polydimethylcyclosiloxane (PCM) ions generated in the electrospray process from ambient air (protonated (Si(CH_3_)_2_O)6; m/z 445.120025) were used for internal recalibration during the analysis [51]. Target ions already selected for MS/MS were dynamically excluded for 30 seconds. General mass spectrometry conditions were: electrospray voltage, 1.9 kV Ion selection threshold was 500 counts for MS/MS, an activation Q-value of 0.25 and activation time of 30 ms was also applied for MS/MS.

The obtained data was searched against the publicly available Tuberculist database version R10 http://genolist.pasteur.fr/TubercuList/ using MASCOT software version 2.1 (Matrix Science, UK). The database was in-house modified to include reversed sequences of the original ORFs in order to determine false-positive thresholds of the Mascot identification engine [52]. Tuberculist was preferred over secondary annotations performed by independent institutes because previous data from our group demonstrated that the Tuberculist annotation appear to be more reliable [33]. The criteria for the Mascot search were as follows: Cysteine carbamidomethylation was set as fixed modification, methionine oxidation and N-acetylation (protein) as variable modifications. Up to 3 missed cleavages were allowed. Peptide (precursor) ion mass tolerance was 15 ppm, and the fragment ion tolerance was 0.5 Da. Mascot scoring showed that p > 0.01 was equivalent to a score of 24. The criterion for a positive identification of proteins identified with at least 2 peptides was a minimal score of 24 for each peptide which represents a 1:10,000 false positive rate at protein level. The maximal score for a peptide from a reversed entry of the annotated *M. tuberculosis *H37Rv database was found to be 31 (data not shown). This was considered as a threshold for false-positive identifications, and all proteins identified in this study with only one peptide were based on a score higher than 37 (25:10,000). No false positive identifications were observed from the reversed database using these criteria. For visualization and validation of spectra, MSQuant version +1.4.2 was used. MSQuant is an open source tool available at http://msquant.sourceforge.net and is widely used for LC-MS/MS data analysis [51].

### Western blot

Proteins from both lipid and aqueous phase were separated by SDS-PAGE, electroblotted to nitrocellulose membranes (Amersham Biosciences) and blocked with 5% non-fat milk in PBS containing 0.5% Tween 20 (PBST) for 1 hour at RT. The membranes were then washed with PBST for 10 min. This was repeated three times. After the last wash, the membranes were incubated overnight at 4°C with rabbit antisera raised against 1) a cell wall fraction and 2) a crude whole cell lysate of *M. bovis *BCG. Sera were diluted 1:500 in PBS with 1% non-fat milk and 0.1% Tween 20. The blots were washed thoroughly with PBST as described above, and probed with Horse Radish Peroxidase (HRP) conjugated anti-rabbit IgG (1:2000 dilution) (Amersham Biosciences) for 1 hour at RT. Antigen-antibody complexes were visualized by a chemiluminescent reaction (Pierce, Rockford, IL, U.S.A.) using Chemidoc XRS (Bio-Rad, Hercules, CA, USA).

### Gene and protein sequence analysis

Gene and protein sequences were obtained from Tuberculist http://genolist.pasteur.fr/TubercuList/ and BoviList http://genolist.pasteur.fr/BoviList/. Sequences alignments were done using the Blast 2 algorithm http://blast.ncbi.nlm.nih.gov/Blast.cgi. For prediction of lipoproteins, the LipoP algorithm was used http://www.cbs.dtu.dk/services/LipoP/. For detection of potential secreted proteins SignalP version 3.0 was used http://www.cbs.dtu.dk/services/SignalP/.

### Estimation of protein abundance

The abundance of each protein was estimated by calculating the protein abundance index (PAI) [53], and the emPAI [15]. The estimation is based on the calculation of identified peptides per protein normalized by the theoretical number of peptides for the same protein. This is considered to be a good method for quantitative estimation because it takes into account that larger proteins are expected to generate more observable peptides in the mass spectrometry analysis, compared to smaller ones [15,16]. The final peptide list obtained from the MS analysis was submitted to a publicly available tool http://empai.iab.keio.ac.jp/, and emPAI values were calculated using the following parameters: *M. tuberculosis *H37Rv Tuberculist version R10 database; trypsin enzyme, carbamidomethyl (C) modification; peptide MW range from 300 to 6000 Da; no retention time filtering; peptide score higher than 24 as filtered by Mascot.

## Authors' contributions

HM contributed to overall conception and design, analysis and interpretation of data, and manuscript drafting. SP cultured *M. tuberculosis *and extracted proteins. TS contributed with protein separation and mass spectrometry analysis. GAdS contributed with LTQ-Orbitrap expertise, data acquisition and critical revision of the data. HGW contributed with design, project coordination, manuscript drafting and critical revision. All authors have read and approved the final manuscript.

## Supplementary Material

Additional file 1 **Figure S1: Collision induced dissociation fragmentation pattern of ion M+2H 1210.62.** The sequence identified by the Mascot engine was CGSPAWDLPTVFGPIAITYNIK_119-140 _from protein Rv0932c. Click here for file

Additional file 2 Table S1: List of observed membrane- and membrane-associated proteins from *M. tuberculosis *H37Rv. Click here for file

Additional file 3 Table S2: List of all observed *M. tuberculosis *H37Rv proteins in the lipid phase of Triton X-114 detergent, sorted by their Sanger IDs. Click here for file

Additional file 4 Table S3: Information about the criteria for protein identifications, such as number of peptides matching each protein, scores, identification threshold and peak lists. Click here for file
