# Supplementary figures and images for: Definition of novel cell envelope associated proteins in Triton X-114 extracts of Mycobacterium tuberculosis H37Rv
Source: BMC Microbiol. 2010 Apr 29;10:132. doi: 10.1186/1471-2180-10-132 (PMC2874799; doi:10.1186/1471-2180-10-132)

## Slide 1
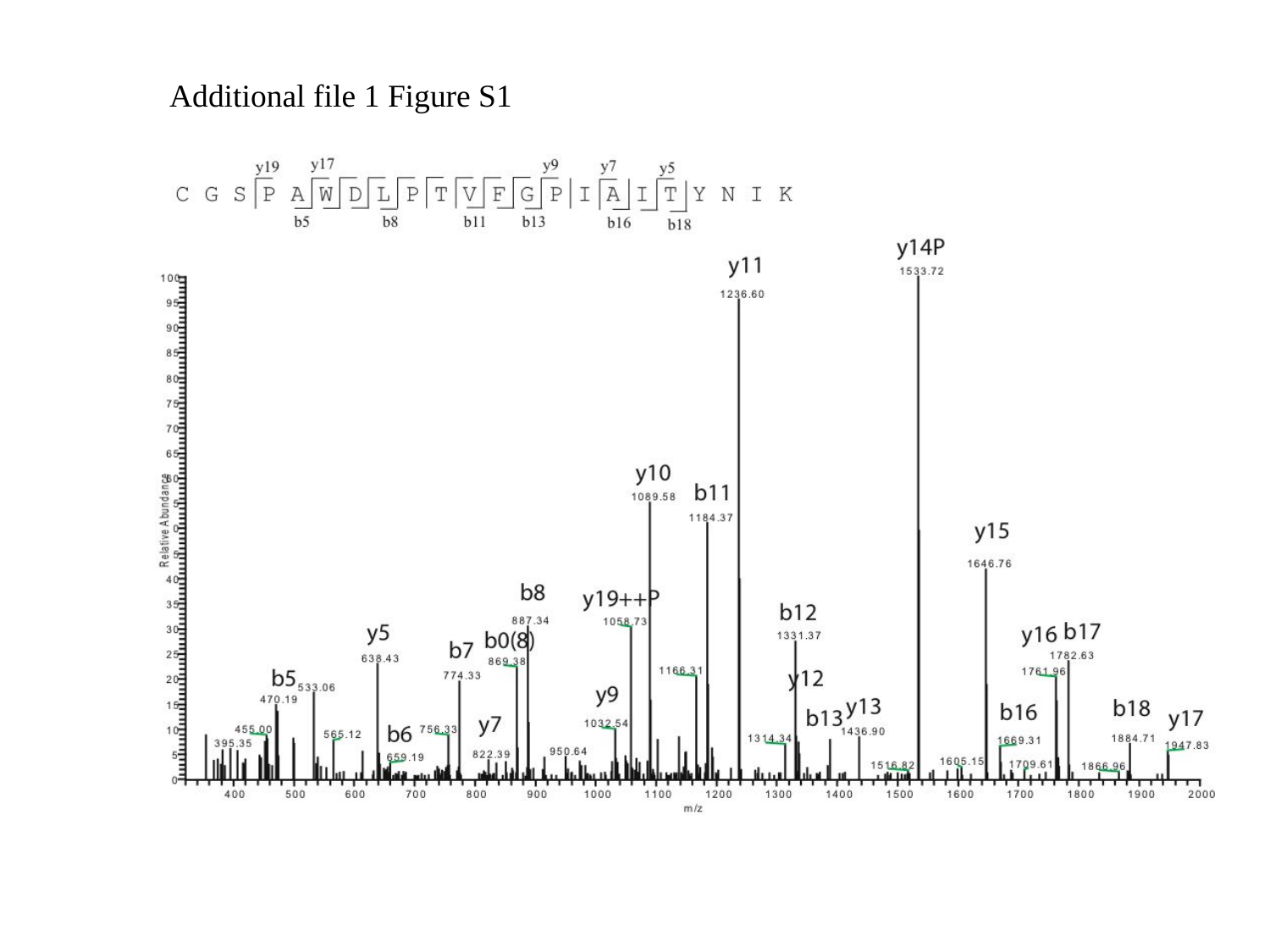

Additional file 1 Figure S1

Supplement: Additional file 1 — Figure S1: Collision induced dissociation fragmentation pattern of ion M+2H 1210.62. The sequence identified by the Mascot engine was CGSPAWDLPTVFGPIAITYNIK119-140 from protein Rv0932c. [file 1471-2180-10-132-S1.PPT]
