# Supplementary material for: Definition of novel cell envelope associated proteins in Triton X-114 extracts of Mycobacterium tuberculosis H37Rv
Source: BMC Microbiol. 2010 Apr 29;10:132. doi: 10.1186/1471-2180-10-132 (PMC2874799; doi:10.1186/1471-2180-10-132)
Supplement: Additional file 2 — Table S1: List of observed membrane- and membrane-associated proteins from M. tuberculosis H37Rv. [file 1471-2180-10-132-S2.DOC]

Additional file 2, Table S1. List of observed membrane and membrane- associated proteins from *M. tuberculosis* H37Rv.

| Sanger ID | Gene  name | protein description | Functional category | Lipoprotein  /OMP | No. of TMH | References |
| --- | --- | --- | --- | --- | --- | --- |
| Rv3156 | *nuoL* | Possible NADH dehydrogenase | 7 | - | 16 |  |
| Rv3910 | *-* | Possible conserved transmembrane protein | 3 | - | 15 | [1-3] |
| Rv3158 | *nuoN* | Possible NADH dehydrogenase | 7 | - | 14 | [1, 3-6] |
| Rv3157 | *nuoM* | Possible NADH dehydrogenase | 7 | - | 14 | [5] |
| Rv1410c | *P55* | Aminoglycosides/tetracycline-transport integral membrane protein | 3 | - | 14 | [1, 5] |
| Rv2846c | *efpA* | Possible integral membrane efflux protein | 3 | - | 14 |  |
| Rv3792 | *-* | Possible conserved transmembrane protein | 3 | - | 13 | [2] |
| Rv3794 | *embA* | Integral membrane indolylacetylinositol arabinosyltransferase | 3 | - | 13 | [1, 2, 5] |
| Rv3793 | *embC* | Integral membrane indolylacetylinositol arabinosyltransferase | 3 | - | 13 | [2, 5] |
| Rv2320c | *rocE* | Possible cationic amino acid transport integral membrane protein | 3 | - | 12 |  |
| Rv1737c | *narK2* | Possible nitrate/nitrite transporter | 3 | - | 12 | [3] |
| Rv2174 | *mptA* | Alpha(1->6)mannosyltransferase | 3 | - | 12 |  |
| Rv2508c | *-* | Possible conserved integral membrane leucine and alanine rich protein | 3 | - | 12 |  |
| Rv1217c | *-* | Possible tetronasin-transport integral membrane protein abc transporter | 3 | - | 12 |  |
| Rv0346c | *ansP2* | Possible l-asparagine permease | 3 | - | 12 |  |
| Rv2127 | *ansP1* | Possible l-asparagine permease | 3 | - | 12 | [5] |
| Rv3043c | *ctaD* | Possible cytochrome c oxidase polypeptide | 7 | - | 12 | [1, 5, 6] |
| Rv0537c | *-* | Possible integral membrane protein | 3 | - | 12 |  |
| Rv2942 | *mmpL7* | Conserved transmembrane transport protein | 3 | - | 12 | [1] |
| Rv0676c | *mmpL5* | Possible conserved transmembrane transport protein | 3 | - | 12 | [1, 2] |
| Rv3795 | *embB* | Integral membrane indolylacetylinositol arabinosyltransferase | 3 | - | 12 | [2, 5] |
| Rv0402c | *mmpL1* | Possible conserved transmembrane transport protein | 3 | - | 12 | [2, 4] |
| Rv0290 | *-* | Possible conserved transmembrane protein | 3 | - | 11 | [1-3, 5] |
| Rv1302 | *rfe* | Possible undecapaprenyl-phosphate alpha-n-acetylglucosaminyltransferase | 3 | - | 11 | [2] |
| Rv0037c | *-* | Possible conserved integral membrane protein | 3 | - | 11 |  |
| Rv2920c | *amt* | Possible ammonium-transport integral membrane protein | 3 | - | 11 |  |
| Rv1707 | *-* | Possible conserved transmembrane protein | 3 | - | 11 |  |
| Rv3877 | *-* | Possible conserved transmembrane protein | 3 | - | 11 |  |
| Rv3476c | *kgtP* | Possible dicarboxylic acid transport integral membrane protein | 3 | - | 11 | [2] |
| Rv1183 | *mmpL10* | Possible conserved transmembrane transport protein | 3 | - | 11 | [2, 4] |
| Rv0450c | *mmpL4* | Possible conserved transmembrane transport protein | 3 | - | 11 | [2, 5] |
| Rv0206c | *mmpL3* | Possible conserved transmembrane transport protein | 3 | - | 11 | [1, 2, 5] |
| Rv3236c | *kefB* | Possible conserved integral membrane transport protein | 3 | - | 10 |  |
| Rv1795 | *-* | Conserved hypothetical membrane protein | 3 | - | 10 | [3] |
| Rv2181 | *-* | Possible alpha(1->2)mannosyltransferase | 3 | - | 10 | [2, 5] |
| Rv1254 | *-* | Possible acyltransferase | 7 | - | 10 |  |
| Rv0226c | *-* | Possible conserved transmembrane protein | 3 | - | 10 | [2] |
| Rv1459c | *-* | Possible conserved integral membrane protein | 3 | - | 10 |  |
| Rv0143c | *-* | Possible conserved transmembrane protein | 3 | - | 10 |  |
| Rv2387 | *-* | Conserved hypothetical protein | 10 | - | 10 | [5] |
| Rv1508c | *-* | Possible membrane protein | 3 | - | 10 | [2] |
| Rv3273 | *-* | Possible transmembrane carbonic anhydrase | 7 | - | 10 | [1-3, 6] |
| Rv2963 | *-* | Possible integral membrane protein | 3 | - | 9 |  |
| Rv0732 | *secY* | Possible preprotein translocase | 3 | - | 9 | [4] |
| Rv2723 | *-* | Possible conserved integral membrane protein | 3 | - | 9 |  |
| Rv3152 | *nuoH* | Possible NADH dehydrogenase | 7 | - | 9 |  |
| Rv1623c | *cydA* | Possible integral membrane cytochrome d ubiquinol oxidase | 7 | - | 9 | [5] |
| Rv0908 | *ctpE* | Possible metal cation transporter atpase p-type | 3 | - | 9 |  |
| Rv2196 | *qcrB* | Possible ubiquinol-cytochrome c reductase | 7 | - | 9 | [1-3, 5] |
| Rv0205 | *-* | Possible conserved transmembrane protein | 3 | - | 8 | [5] |
| Rv0935 | *pstC1* | Phosphate-transport integral membrane abc transporter | 3 | - | 8 |  |
| Rv1101c | *-* | Conserved membrane protein | 3 | - | 8 | [1, 5] |
| Rv2113 | *-* | Possible integral membrane protein | 3 | - | 8 | [1, 3] |
| Rv1622c | *cydB* | Possible integral membrane cytochrome d ubiquinol oxidase | 7 | - | 8 |  |
| Rv2673 | *-* | Possible conserved integral membrane protein | 3 | - | 8 | [5] |
| Rv1002c | *-* | Conserved membrane protein | 3 | - | 8 |  |
| Rv1132 | *-* | Conserved membrane protein | 3 | - | 8 | [2] |
| Rv2994 | *-* | Possible conserved integral membrane protein | 3 | - | 8 | [4] |
| Rv1431 | *-* | Conserved membrane protein | 3 | - | 8 |  |
| Rv2877c | *merT* | Possible conserved integral membrane protein | 3 | - | 7 |  |
| Rv1072 | *-* | Possible conserved transmembrane protein | 3 | - | 7 |  |
| Rv2881c | *cdsA* | Possible integral membrane phosphatidate cytidylyltransferase | 1 | - | 7 |  |
| Rv0110 | *-* | Possible conserved integral membrane protein | 3 | - | 7 |  |
| Rv3806c | *-* | Possible conserved integral membrane protein | 3 | - | 7 | [1, 3, 5] |
| Rv2443 | *dctA* | Possible c4-dicarboxylate-transport transmembrane protein | 3 | - | 7 |  |
| Rv1030 | *kdpB* | Possible potassium-transporting p-type ATPase b chain | 3 | - | 7 |  |
| Rv3193c | *-* | Possible conserved transmembrane protein | 3 | - | 7 | [1, 3-5] |
| Rv2051c | *ppm1* | Polyprenol-monophosphomannose synthase | 3 | - | 7 | [2, 3] |
| Rv1624c | *-* | Possible conserved membrane protein | 3 | - | 6 |  |
| Rv0167 | *yrbE1A* | Conserved hypothetical integral membrane protein | 0 | - | 6 | [3, 5] |
| Rv3481c | *-* | Possible integral membrane protein | 3 | - | 6 | [5] |
| Rv2938 | *drrC* | Possible daunorubicin-dim-transport integral membrane protein abc transporter | 3 | - | 6 |  |
| Rv1858 | *modB* | Possible molbdenum-transport integral membrane protein abc transporter | 3 | - | 6 |  |
| Rv3501c | *yrbE4A* | Conserved hypothetical integral membrane protein | 0 | - | 6 |  |
| Rv1237 | *sugB* | Possible sugar-transport integral membrane protein abc transporter | 3 | - | 6 |  |
| Rv0359 | *-* | Possible conserved integral membrane protein | 3 | - | 6 |  |
| Rv3335c | *-* | Possible conserved integral membrane protein | 3 | - | 6 |  |
| Rv2937 | *drrB* | Possible daunorubicin-dim-transport integral membrane protein abc transporter | 3 | - | 6 |  |
| Rv2693c | *-* | Possible conserved integral membrane alanine and leucine rich protein | 3 | - | 6 | [4] |
| Rv0308 | *-* | Possible conserved integral membrane protein | 3 | - | 6 | [5, 6] |
| Rv0936 | *pstA2* | Phosphate-transport integral membrane abc transporter | 3 | - | 6 |  |
| Rv3689 | *-* | Possible conserved transmembrane protein | 3 | - | 6 |  |
| Rv2093c | *tatC* | Possible sec-independent protein translocase transmembrane protein | 3 | - | 6 | [2] |
| Rv3694c | *-* | Possible conserved transmembrane protein | 3 | - | 6 |  |
| Rv3783 | *rfbD* | Possible o-antigen/lipopolysaccharide transport integral membrane protein abc transporter | 3 | - | 6 |  |
| Rv0180c | *-* | Possible conserved transmembrane protein | 3 | - | 6 | [1, 3, 5, 6] |
| Rv1273c | *-* | Possible drugs-transport transmembrane ATP-binding protein abc transporter | 3 | - | 6 |  |
| Rv0969 | *ctpV* | Possible metal cation transporter p-type ATPase | 3 | - | 6 | [1, 3] |
| Rv0235c | *-* | Possible conserved transmembrane protein | 3 | - | 6 |  |
| Rv1819c | *-* | Possible drugs-transport transmembrane ATP-binding protein abc transporter | 3 | - | 6 | [1-3] |
| Rv2586c | *secF* | Possible protein-export membrane protein | 3 | - | 6 | [1-3, 5] |
| Rv2587c | *secD* | Possible protein-export membrane protein | 3 | - | 6 | [1-3, 5] |
| Rv1747 | *-* | Possible conserved transmembrane ATP-binding protein abc transporter | 3 | - | 6 | [2, 3] |
| Rv1614 | *lgt* | Possible prolipoprotein diacylglyceryl transferases | 3 | - | 6 | [1, 3] |
| Rv0338c | *-* | Possible iron-sulfur-binding reductase | 7 | - | 6 | [1-3] |
| Rv2120c | *-* | Possible conserved integral membrane protein | 3 | - | 5 | [3, 5] |
| Rv3756c | *proZ* | Possible osmoprotectant | 0 | - | 5 |  |
| Rv2136c | *-* | Possible conserved transmembrane protein | 3 | - | 5 |  |
| Rv1304 | *atpB* | Possible ATP synthase a chain | 7 | - | 5 | [1, 3, 5] |
| Rv3500c | *yrbE4B* | Conserved hypothetical integral membrane protein | 0 | - | 5 |  |
| Rv0168 | *yrbE1B* | Conserved hypothetical integral membrane protein | 0 | - | 5 |  |
| Rv0364 | *-* | Possible conserved transmembrane protein | 3 | - | 5 |  |
| Rv1236 | *sugA* | Possible sugar-transport integral membrane protein abc transporter | 3 | - | 5 |  |
| Rv1216c | *-* | Possible conserved integral membrane protein | 3 | - | 5 |  |
| Rv0625c | *-* | Possible conserved transmembrane protein | 3 | - | 5 |  |
| Rv3238c | *-* | Possible conserved integral membrane protein | 3 | - | 5 |  |
| Rv0218 | *-* | Possible conserved transmembrane protein | 3 | - | 5 |  |
| Rv1164 | *narI* | Possible respiratory nitrate reductase | 7 | - | 5 |  |
| Rv1226c | *-* | Possible transmembrane protein | 3 | - | 5 | [2] |
| Rv0249c | *-* | Possible succinate dehydrogenase | 7 | - | 5 | [5] |
| Rv1069c | *-* | Conserved hypothetical protein | 10 | - | 5 | [1, 2] |
| Rv2620c | *-* | Possible conserved transmembrane protein | 3 | - | 4 |  |
| Rv2746c | *pgsA3* | Possible pgp synthase | 1 | - | 4 |  |
| Rv3092c | *-* | Possible conserved integral membrane protein | 3 | - | 4 | [2] |
| Rv1822 | *pgsA2* | Possiblecdp-diacylglycerol--glycerol-3-phosphate 3-phosphatidyltransferase | 1 | - | 4 | [5] |
| Rv2563 | *-* | Possible glutamine-transport transmembrane protein abc transporter | 3 | - | 4 | [1, 3-5] |
| Rv2637 | *dedA* | Possible transmembrane protein | 3 | - | 4 | [5] |
| Rv0072 | *-* | Possible glutamine-transport transmembrane protein abc transporter | 3 | - | 4 | [1, 3] |
| Rv3671c | *-* | Possible membrane-associated serine protease | 7 | - | 4 | [1, 4] |
| Rv2197c | *-* | Possible conserved transmembrane protein | 3 | - | 4 | [3, 5] |
| Rv3069 | *-* | Possible conserved transmembrane protein | 3 | - | 4 | [5] |
| Rv2560 | *-* | Possible proline and glycine rich transmembrane protein | 3 | - | 4 | [5] |
| Rv2869c | *rip* | Membrane bound metalloprotease | 3 | - | 4 | [7] |
| Rv3435c | *-* | Possible conserved transmembrane protein | 3 | - | 4 | [2] |
| Rv3101c | *ftsX* | Putative cell division protein | 3 | - | 4 | [1-3, 5] |
| Rv2732c | *-* | Possible conserved transmembrane protein | 3 | - | 4 |  |
| Rv1610 | *-* | Possible conserved membrane protein | 3 | - | 4 |  |
| Rv1749c | *-* | Possible integral membrane protein | 3 | - | 4 | [6] |
| Rv2326c | *-* | Possible transmembrane ATP-binding protein abc transorter | 3 | - | 4 | [1, 3] |
| Rv0563 | *htpX* | Possible protease transmembrane protein heat shock protein | 0 | - | 4 | [1, 4-6] |
| Rv3277 | *-* | Possible conserved transmembrane protein | 3 | - | 4 | [5] |
| Rv2536 | *-* | Possible conserved transmembrane protein | 3 | - | 4 | [1, 5-7] |
| Rv0954 | *-* | Possible conserved transmembrane protein | 3 | - | 4 | [1, 5, 6] |
| Rv0412c | *-* | Possible conserved membrane protein | 3 | - | 4 | [1, 6] |
| Rv3723 | *-* | Possible conserved transmembrane protein | 3 | - | 4 | [1, 3, 5, 6] |
| Rv1779c | *-* | Hypothetical integral membrane protein | 3 | - | 4 | [2] |
| Rv0870c | *-* | Possible conserved integral membrane protein | 3 | - | 3 |  |
| Rv3316 | *sdhC* | Possible succinate dehydrogenase | 7 | - | 3 |  |
| Rv3145 | *nuoA* | Possible NADH dehydrogenase | 7 | - | 3 | [5] |
| Rv1924c | *-* | hypothetical protein | 10 | - | 3 | [1] |
| Rv2617c | *-* | Possible transmembrane protein | 3 | - | 3 |  |
| Rv2612c | *pgsA1* | Possible pi synthase | 1 | - | 3 | [5] |
| Rv1342c | *-* | Conserved membrane protein | 3 | - | 3 | [1] |
| Rv3807c | *-* | Possible conserved transmembrane protein | 3 | - | 3 | [3] |
| Rv0461 | *-* | Possible transmembrane protein | 3 | - | 3 |  |
| Rv3632 | *-* | Possible conserved membrane protein | 3 | - | 3 |  |
| Rv3104c | *-* | Possible conserved transmembrane protein | 3 | - | 3 | [5] |
| Rv3695 | *-* | Possible conserved membrane protein | 3 | - | 3 | [3] |
| Rv3200c | *-* | Possible transmembrane cation transporter | 3 | - | 3 | [1-3] |
| Rv1481 | *-* | Possible membrane protein | 3 | - | 3 | [1, 3, 5, 6] |
| Rv0093c | *-* | Possible conserved membrane protein | 3 | - | 3 | [2] |
| Rv1272c | *-* | Possible drugs-transport transmembrane ATP-binding protein abc transporter | 3 | - | 3 |  |
| Rv3317 | *sdhD* | Possible succinate dehydrogenase | 7 | - | 3 |  |
| Rv0176 | *-* | Possible conserved mce associated transmembrane protein | 3 | - | 3 | [3, 5] |
| Rv3479 | *-* | Possible transmembrane protein | 3 | - | 3 | [1, 2] |
| Rv2284 | *lipM* | Possible esterase | 7 | - | 3 | [1-3, 5] |
| Rv2235 | *-* | Possible conserved transmembrane protein | 3 | - | 3 | [2] |
| Rv2200c | *ctaC* | Possible transmembrane cytochrome c oxidase | 7 | Lipo | 3 | [1, 3-6] |
| Rv2345 | *-* | Possible conserved transmembrane protein | 3 | - | 3 | [1-3, 6] |
| Rv2195 | *qcrA* | Possible rieske iron-sulfur protein | 7 | - | 3 | [1-3, 5-7] |
| Rv0497 | *-* | Possible conserved transmembrane protein | 3 | - | 3 | [5] |
| Rv2146c | *-* | Possible conserved transmembrane protein | 3 | - | 2 |  |
| Rv0476 | *-* | Possible conserved transmembrane protein | 3 | - | 2 |  |
| Rv0544c | *-* | Possible conserved transmembrane protein | 3 | - | 2 |  |
| Rv1487 | *-* | Conserved membrane protein | 3 | - | 2 | [6] |
| Rv0879c | *-* | Possible conserved transmembrane protein | 3 | - | 2 |  |
| Rv0011c | *-* | Possible conserved transmembrane protein | 3 | - | 2 |  |
| Rv3851 | *-* | Possible membrane protein | 3 | - | 2 |  |
| Rv1417 | *-* | Possible conserved membrane protein | 3 | - | 2 | [3] |
| Rv0531 | *-* | Possible conserved membrane protein | 3 | - | 2 | [6] |
| Rv2698 | *-* | Possible conserved alanine rich transmembrane protein | 3 | - | 2 |  |
| Rv0514 | *-* | Possible transmembrane protein | 3 | - | 2 |  |
| Rv0556 | *-* | Possible conserved transmembrane protein | 3 | - | 2 | [3] |
| Rv0985c | *mscL* | Possible large-conductance ion mechanosensitive channel | 3 | - | 2 | [1, 3] |
| Rv0426c | *-* | Possible transmembrane protein | 3 | - | 2 |  |
| Rv2843 | *-* | Possible conserved transmembrane alanine rich protein | 3 | Lipo | 2 |  |
| Rv0010c | *-* | Possible conserved membrane protein | 3 | - | 2 | [5] |
| Rv3669 | *-* | Possible conserved transmembrane protein | 3 | - | 2 | [1] |
| Rv0686 | *-* | Possible membrane protein | 3 | - | 2 | [1, 3] |
| Rv3278c | *-* | Possible conserved transmembrane protein | 3 | - | 2 | [3, 5, 6] |
| Rv1234 | *-* | Possible transmembrane protein | 3 | - | 2 | [1, 3] |
| Rv2772c | *-* | Possible conserved transmembrane protein | 3 | - | 2 |  |
| Rv0680c | *-* | Possible conserved transmembrane protein | 3 | - | 2 | [4, 5] |
| Rv3732 | *-* | Conserved hypothetical protein | 10 | - | 2 | [1] |
| Rv1227c | *-* | Possible transmembrane protein | 3 | - | 2 |  |
| Rv1078 | *pra* | Possible proline-rich antigen homolog | 10 | - | 2 | [3, 5, 6] |
| Rv3826 | *fadD23* | Possible fatty-acid-CoA ligase | 1 | - | 2 |  |
| Rv1615 | *-* | Possible hypothetical membrane protein | 3 | - | 2 |  |
| Rv0513 | *-* | Possible conserved transmembrane protein | 3 | - | 2 | [5] |
| Rv3090 | *-* | Hypothetical alanine and valine rich protein | 10 | - | 2 | [1, 3, 5, 6] |
| Rv1796 | *mycP5* | Possible proline rich membrane-anchored mycosin | 7 | - | 2 | [5] |
| Rv0292 | *-* | Possible conserved transmembrane protein | 3 | - | 2 | [1, 3] |
| Rv2219 | *-* | Possible conserved transmembrane protein | 3 | - | 2 | [1, 2, 5] |
| Rv2194 | *qcrC* | Possible ubiquinol-cytochrome c reductase | 7 | - | 2 | [1, 5, 6] |
| Rv1239c | *corA* | Possible magnesium and cobalt transport transmembrane protein | 3 | - | 2 | [1, 5] |
| Rv2721c | *-* | Possible conserved transmembrane alanine and glycine rich protein | 3 | - | 2 | [1, 3-6] |
| Rv2169c | *-* | Possible conserved transmembrane protein | 3 | - | 2 |  |
| Rv0007 | *-* | Possible conserved membrane protein | 3 | - | 2 | [1, 8] [3] |
| Rv3165c | *-* | hypothetical protein | 10 | - | 2 | [2] |
| Rv1231c | *-* | Possible membrane protein | 3 | - | 2 | [2] |
| Rv1343c | *lprD* | Possible conserved lipoprotein | 3 | Lipo | 2 |  |
| Rv0227c | *-* | Possible conserved membrane protein | 3 | - | 2 | [1, 5, 9] |
| Rv1489 | *-* | Conserved hypothetical protein | 10 | - | 2 | [1] |
| Rv2219A | *-* | Possible conserved membrane protein | 3 | - | 2 |  |
| Rv3004 | *cfp6* | Low molecular weight protein antigen | 3 | Omp | 1 | [4] |
| Rv0506 | *mmpS2* | Possible conserved membrane protein | 3 | Omp | 1 | [4] |
| Rv2376c | *cfp2* | Low molecular weight antigen | 3 | Omp | 1 | [4, 9] |
| Rv1209 | *-* | Conserved hypothetical protein | 10 | Omp | 1 | [3, 5] |
| Rv0677c | *mmpS5* | Possible conserved membrane protein | 3 | Omp | 1 | [4] |
| Rv3584 | *lpqE* | Possible conserved lipoprotein | 3 | Lipo | 1 | [1, 3-6] |
| Rv0817c | *-* | Possible conserved exported protein | 3 | Omp | 1 |  |
| Rv0291 | *mycP3* | Possible membrane-anchored mycosin | 7 | - | 1 | [1, 3, 4, 6] |
| Rv3883c | *mycP1* | Membrane-anchored mycosin | 7 | - | 1 |  |
| Rv3627c | *-* | Conserved hypothetical protein | 10 | Omp | 1 | [1, 4] |
| Rv0309 | *-* | Possible conserved exported protein | 3 | Omp | 1 | [4] |
| Rv2537c | *aroD* | 3-dehydroquinate dehydratase | 7 | - | 1 |  |
| Rv2980 | *-* | Possible conserved secreted protein | 3 | Omp | 1 |  |
| Rv3864 | *-* | Conserved hypothetical protein | 10 | - | 1 |  |
| Rv0052 | *-* | Conserved hypothetical protein | 10 | - | 1 | [3, 5, 9] |
| Rv0392c | *ndhA* | Possible membrane NADH dehydrogenase | 7 | - | 1 | [2, 3] |
| Rv2138 | *lppL* | Possible conserved lipoprotein | 3 | Lipo | 1 | [5] |
| Rv0169 | *mce1A* | Mce-family protein | 0 | Omp | 1 | [2] |
| Rv2171 | *lppM* | Possible conserved lipoprotein | 3 | Lipo | 1 | [5] |
| Rv1382 | *-* | Possible export or membrane protein | 3 | Omp | 1 | [4] |
| Rv2672 | *-* | Possible secreted protease | 7 | Lipo/Omp | 1 | [1, 4, 5] |
| Rv0283 | *-* | Possible conserved membrane protein | 3 | - | 1 | [1-4] |
| Rv2597 | *-* | Possible membrane protein | 3 | Omp | 1 | [1, 3, 5, 7] |
| Rv0983 | *pepD* | Possible serine protease | 7 | - | 1 |  |
| Rv1476 | *-* | Possible membrane protein | 3 | - | 1 | [3, 5, 6] |
| Rv0314c | *-* | Possible conserved membrane protein | 3 | - | 1 | [5] |
| Rv3497c | *mce4C* | Mce-family protein | 0 | Omp | 1 | [1] |
| Rv1270c | *lprA* | Possible lipoprotein | 3 | Lipo | 1 | [1-6] |
| Rv2945c | *lppX* | Possible conserved lipoprotein | 3 | Lipo | 1 | [1-6] |
| Rv3693 | *-* | Possible conserved membrane protein | 3 | Omp | 1 | [2, 4] |
| Rv0538 | *-* | Possible conserved membrane protein | 3 | - | 1 | [5] |
| Rv1418 | *lprH* | Possible lipoprotein | 3 | Lipo | 1 |  |
| Rv3212 | *-* | Conserved hypothetical alanine valine rich protein | 10 | Omp | 1 | [1, 2] |
| Rv3764c | *tcrY* | Possible two component sensor kinase | 9 | - | 1 | [2] |
| Rv0419 | *lpqM* | Possible lipoprotein peptidase | 3 | Lipo | 1 | [5] |
| Rv0172 | *mce1D* | Mce-family protein | 0 | Omp | 1 | [1, 4] |
| Rv1368 | *lprF* | Possible conserved lipoprotein | 3 | Lipo | 1 | [1-3, 5, 6] |
| Rv1825 | *-* | Conserved hypothetical protein | 10 | - | 1 | [1] |
| Rv1782 | *-* | Possible conserved membrane protein | 3 | - | 1 | [3] |
| Rv3683 | *-* | Conserved hypothetical protein | 10 | Omp | 1 | [3, 9] |
| Rv0931c | *pknD* | Transmembrane serine/threonine-protein kinase | 9 | - | 1 | [1-3] |
| Rv0418 | *lpqL* | Possible lipoprotein aminopeptidase | 3 | Lipo | 1 | [1-3, 5, 6] |
| Rv0170 | *mce1B* | Mce-family protein | 0 | Omp | 1 | [3, 4] |
| Rv2330c | *lppP* | Possible lipoprotein | 3 | Lipo | 1 |  |
| Rv0173 | *lprK* | Possible mce-family lipoprotein | 3 | Lipo | 1 | [1, 4] |
| Rv1022 | *lpqU* | Possible conserved lipoprotein | 3 | Lipo | 1 | [5] |
| Rv0174 | *mce1F* | Mce-family protein | 0 | Omp | 1 | [2, 4, 5, 8] |
| Rv1836c | *-* | Conserved hypothetical protein | 10 | - | 1 | [1, 3, 5, 6] |
| Rv3390 | *lpqD* | Possible conserved lipoprotein | 3 | Lipo | 1 | [1, 3, 5, 7] |
| Rv1097c | *-* | Possible membrane glycine and proline rich protein | 3 | - | 1 | [1, 2, 5, 6] |
| Rv1488 | *-* | Possible exported conserved protein | 3 | Omp | 1 | [1-7] |
| Rv0144 | *-* | Possible transcriptional regulatory protein | 9 | - | 1 | [2, 3] |
| Rv0888 | *-* | Possible exported protein | 3 | Omp | 1 |  |
| Rv0899 | *ompA* | Outer membrane protein |  | Omp | 1 | [1, 5] |
| Rv1881c | *lppE* | Possible conserved lipoprotein | 3 | Lipo | 1 | [2] |
| Rv1184c | *-* | Possible exported protein | 3 | Omp | 1 |  |
| Rv0592 | *mce2D* | Mce-family protein | 0 | Omp | 1 | [2] |
| Rv1566c | *-* | Possible inv protein | 0 | Omp | 1 |  |
| Rv3587c | *-* | Possible conserved membrane protein | 3 | Omp | 1 | [4, 5] |
| Rv2518c | *lppS* | Possible conserved lipoprotein | 3 | Lipo | 1 | [5] |
| Rv1223 | *htrA* | Possible serine protease | 7 | - | 1 | [1, 2, 6] |
| Rv0048c | *-* | Possible membrane protein | 3 | - | 1 | [1, 3, 5-7] |
| Rv1280c | *oppA* | Possible periplasmic oligopeptide-binding lipoprotein | 3 | Lipo | 1 | [1, 2, 6, 7] |
| Rv0638 | *secE1* | Possible preprotein translocase | 3 | - | 1 | [1, 5] |
| Rv3802c | *-* | Possible conserved membrane protein | 3 | Omp | 1 | [1, 3, 5, 6] |
| Rv0171 | *mce1C* | Mce-family protein | 0 | Omp | 1 | [5] |
| Rv0014c | *pknB* | transmembrane serine/threonine-protein kinase | 9 | - | 1 | [5] |
| Rv2203 | *-* | Possible conserved membrane protein | 3 | - | 1 | [5] |
| Rv0177 | *-* | Possible conserved mce associated protein | 10 | Lipo | 1 | [2, 3] |
| Rv1746 | *pknF* | Anchored-membrane serine/threonine-protein kinase | 9 | - | 1 |  |
| Rv2473 | *-* | Possible alanine and proline rich membrane protein | 3 | - | 1 |  |
| Rv1977 | *-* | Conserved hypothetical protein | 10 | - | 1 | [2, 3] |
| Rv0988 | *-* | Possible conserved exported protein | 3 | Omp | 1 |  |
| Rv2599 | *-* | Possible conserved membrane protein | 3 | Omp | 1 |  |
| Rv0479c | *-* | Possible conserved membrane protein | 3 | - | 1 | [1, 3, 6] |
| Rv2224c | *-* | Possible exported protease | 3 | Lipo/Omp | 1 | [1-7] |
| Rv2903c | *lepB* | Possible signal peptidase | 3 | - | 1 | [1, 6] |
| Rv3484 | *cpsA* | Possible conserved protein | 10 | Omp | 1 | [2, 4] |
| Rv3690 | *-* | Possible conserved membrane protein | 3 | - | 1 |  |
| Rv0008c | *-* | Possible membrane protein | 3 | - | 1 | [1, 3, 5] |
| Rv2080 | *lppJ* | Possible lipoprotein | 3 | Lipo | 1 | [4, 6] |
| Rv2507 | *-* | Possible conserved proline rich membrane protein | 3 | - | 1 |  |
| Rv1363c | *-* | Possible membrane protein | 3 | - | 1 |  |
| Rv1754c | *-* | Conserved hypothetical protein | 10 | - | 1 | [2] |
| Rv0361 | *-* | Possible conserved membrane protein | 3 | - | 1 | [1, 5] |
| Rv2198c | *mmpS3* | Possible conserved membrane protein | 3 | - | 1 | [1, 3, 5] |
| Rv3852 | *hns* | Possible histone-like protein | 2 | - | 1 | [1, 6] |
| Rv2091c | *-* | Possible membrane protein | 3 | - | 1 | [1, 3, 5-7] |
| Rv1386 | *PE15* | PE family protein | 6 | Omp | 0 | [4] |
| Rv1857 | *modA* | Possible molybdate-binding lipoprotein | 3 | Lipo | 0 |  |
| Rv1244 | *lpqZ* | Possible lipoprotein | 3 | Lipo | 0 | [2, 5] |
| Rv2873 | *mpt83* | Cell surface lipoprotein | 3 | Lipo | 0 | [1, 3-5] |
| Rv1914c | *-* | Hypothetical protein | 10 | Omp | 0 | [3] |
| Rv1325c | *PE_PGRS24* | PE-PGRS family protein | 6 | Omp | 0 |  |
| Rv0999 | *-* | Hypothetical protein | 10 | Lipo/Omp | 0 | [1, 3-5] |
| Rv1275 | *lprC* | Possible lipoprotein | 3 | Lipo | 0 | [2, 3, 5, 6] |
| Rv3298c | *lpqC* | Possible esterase lipoprotein | 3 | Lipo | 0 | [1] |
| Rv0237 | *lpqI* | Possible conserved lipoprotein | 3 | Lipo | 0 | [1, 2, 4-6] |
| Rv3763 | *lpqH* | 19 kDa lipoprotein antigen precursor | 3 | Lipo | 0 | [1, 3, 5-7, 9] |
| Rv2046 | *lppI* | Possible lipoprotein | 3 | Lipo | 0 | [3, 5] |
| Rv3705c | *-* | Conserved hypothetical protein | 10 | Omp | 0 | [4] |
| Rv0934 | *pstS1* | Periplasmic phosphate-binding lipoprotein | 3 | Lipo | 0 | [1-7] |
| Rv0265c | *fecB2* | Possible periplasmic iron-transport lipoprotein | 3 | Lipo | 0 | [3-6] |
| Rv2565 | *-* | Conserved hypothetical protein | 10 | Omp | 0 | [2] |
| Rv1166 | *lpqW* | Possible conserved lipoprotein | 3 | Lipo | 0 | [4-6] |
| Rv0411c | *glnH* | Possible glutamine-binding lipoprotein | 3 | Lipo | 0 | [4, 5] |
| Rv0241c | *-* | Double hotdog hydratase | 7 | Omp | 0 | [3, 7] |
| Rv1016c | *lpqT* | Possible conserved lipoprotein | 3 | Lipo | 0 | [1] |
| Rv2251 | *-* | Possible flavoprotein | 7 | Omp | 0 | [2-4] |
| Rv0604 | *lpqO* | Possible conserved lipoprotein | 3 | Lipo | 0 | [3] |
| Rv1339 | *-* | Conserved hypothetical protein | 10 | Omp | 0 | [3] |
| Rv1235 | *lpqY* | Possible sugar-binding lipoprotein | 3 | Lipo | 0 | [5] |
| Rv3796 | *atsH* | Conserved hypothetical protein | 10 | Lipo/Omp | 0 | [5] |
| Rv0838 | *lpqR* | Possible conserved lipoprotein | 3 | Lipo | 0 | [4] |
| Rv3244c | *lpqB* | Possible conserved lipoprotein | 3 | Lipo | 0 | [1, 2, 4-6] |
| Rv0906 | *-* | Conserved hypothetical protein | 10 | Omp | 0 | [1, 3, 5, 6, 9] |
| Rv0679c | *-* | Conserved hypothetical threonine rich protein | 10 | Omp | 0 | [3, 5] |
| Rv3623 | *lpqG* | Possible conserved lipoprotein | 3 | Lipo | 0 | [1-3] |
| Rv0432 | *sodC* | Possible periplasmic superoxide dismutase | 0 | Lipo | 0 | [1, 3, 5-7] |
| Rv3878 | *-* | Conserved hypothetical alanine rich protein | 10 | Omp | 0 |  |
| Rv3006 | *lppZ* | Possible conserved lipoprotein | 3 | Lipo | 0 | [1, 3-6] |
| Rv3572 | *-* | Hypothetical protein | 10 | Omp | 0 | [4] |
| Rv3576 | *lppH* | Possible conserved lipoprotein | 3 | Lipo | 0 | [5, 7] |
| Rv0799c | *-* | Conserved hypothetical protein | 10 | Omp | 0 | [1] |
| Rv1899c | *lppD* | Possible lipoprotein | 3 | Lipo | 0 | [3, 4, 9] |
| Rv2068c | *blaC* | Class a beta-lactamase | 7 | Lipo | 0 | [1-5] |
| Rv0399c | *lpqK* | Possible conserved lipoprotein | 3 | Lipo | 0 |  |
| Rv2116 | *lppK* | Possible conserved lipoprotein | 3 | Lipo | 0 | [1, 3, 5] |
| Rv3033 | *-* | Hypothetical protein | 10 | Omp | 0 | [3-6] |
| Rv2784c | *lppU* | Possible lipoprotein | 3 | Lipo | 0 |  |
| Rv2999 | *lppY* | Possible conserved lipoprotein | 3 | Lipo | 0 | [1-3, 5, 6] |
| Rv2394 | *ggtB* | Possible gamma-glutamyltranspeptidase precursor | 7 | Lipo | 0 | [1, 3, 5] |
| Rv1784 | *-* | Conserved hypothetical protein | 10 | Omp | 0 | [2, 4, 5] |
| Rv1411c | *lprG* | Possible conserved lipoprotein | 3 | Lipo | 0 | [1-3, 5-7] |
| Rv2041c | *-* | Possible sugar-binding lipoprotein | 3 | Lipo | 0 | [2, 5] |
| Rv1252c | *lprE* | Possible lipoprotein | 3 | Lipo | 0 | [4] |
| Rv0526 | *-* | Possible thioredoxin protein | 7 | Lipo | 0 | [1, 3-6] |
| Rv0932c | *pstS2* | Periplasmic phosphate-binding lipoprotein | 3 | Lipo | 0 | [1, 3-6] |
| Rv0928 | *pstS3* | Periplasmic phosphate-binding lipoprotein | 3 | Lipo | 0 | [1, 4-6] |
| Rv1424c | *-* | Possible membrane protein | 3 | Lipo | 0 | [2] |
| Rv0583c | *lpqN* | Possible conserved lipoprotein | 3 | Lipo | 0 | [1, 3-5] |
| Rv2112c | *-* | Conserved hypothetical protein | 10 | Omp | 0 | [3] |
| Rv0846c | *-* | Possible oxidase | 7 | Lipo | 0 |  |
| Rv2833c | *ugpB* | Possible sn-glycerol-3-phosphate-binding lipoprotein | 3 | Lipo | 0 | [2, 5] |
| Rv1274 | *lprB* | Possible lipoprotein | 3 | Lipo | 0 | [5] |
| Rv1006 | *-* | hypothetical protein | 10 | Omp | 0 | [1-3, 5-7] |
| Rv0088 | *-* | hypothetical protein | 10 | Omp | 0 | [2, 3, 5, 9] |
| Rv3547 | *-* | Conserved hypothetical protein | 10 | Omp | 0 | [1-3, 7] |

Reference List

1. Xiong Y, Chalmers MJ, Gao FP, Cross TA, and Marshall AG: **Identification of *Mycobacterium tuberculosis* H37Rv integral membrane proteins by one-dimensional gel electrophoresis and liquid chromatography electrospray ionization tandem mass spectrometry**. *J.Proteome.Res.* 2005*,* **4:** 855-861.

2. Mawuenyega KG, Forst CV, Dobos KM, Belisle JT, Chen J, Bradbury EM, Bradbury AR, and Chen X: ***Mycobacterium tuberculosis* functional network analysis by global subcellular protein profiling**. *Mol.Biol.Cell* 2005*,* **16:** 396-404.

3. Gu SL, Chen J, Dobos KM, Bradbury EM, Belisle JT, and Chen X: **Comprehensive proteomic profiling of the membrane constituents of a *Mycobacterium tuberculosis* strain.** *Mol.Cell Proteomics* 2003, **2**:1284-1296.

4. Malen H, Berven FS, Fladmark KE, and Wiker HG: **Comprehensive analysis of exported proteins from *Mycobacterium tuberculosis* H37Rv**. *Proteomics* 2007, **7**:1702-1718.

5. Malen H, Berven FS, Softeland T, Arntzen MO, D'Santos CS, De Souza GA, and Wiker HG: **Membrane and membrane-associated proteins in Triton X-114 extracts of *Mycobacterium bovis* BCG identified using a combination of gel-based and gel-free fractionation strategies**. *Proteomics.* 2008*,* **8:** 1859-1870.

6. Mattow J, Siejak F, Hagens K, Schmidt F, Koehler C, Treumann A, Schaible UE, and Kaufmann SH: **An improved strategy for selective and efficient enrichment of integral plasma membrane proteins of mycobacteria**. *Proteomics.* 2007*,* **7:** 1687-1701.

7. Sinha S, Kosalai K, Arora S, Namane A, Sharma P, Gaikwad AN, Brodin P, and Cole ST: **Immunogenic membrane-associated proteins of *Mycobacterium tuberculosis* revealed by proteomics**. *Microbiology* 2005*,* **151:** 2411-2419.

8. Schmidt F, Donahoe S, Hagens K, Mattow J, Schaible UE, Kaufmann SH, Aebersold R, and Jungblut PR: **Complementary analysis of the *Mycobacterium tuberculosis* proteome by two-dimensional electrophoresis and isotope-coded affinity tag technology**. *Mol.Cell Proteomics* 2004. **3**:24-42.

9. Rosenkrands I, King A, Weldingh K, Moniatte M, Moertz E, and Andersen P: **Towards the proteome of *Mycobacterium tuberculosis***. *Electrophoresis* 2000*,* **21:** 3740-3756.
