# Supplementary material for: Definition of novel cell envelope associated proteins in Triton X-114 extracts of Mycobacterium tuberculosis H37Rv
Source: BMC Microbiol. 2010 Apr 29;10:132. doi: 10.1186/1471-2180-10-132 (PMC2874799; doi:10.1186/1471-2180-10-132)
Supplement: Additional file 3 — Table S2: List of all observed M. tuberculosis H37Rv proteins in the lipid phase of Triton X-114 detergent, sorted by their Sanger IDs. [file 1471-2180-10-132-S3.DOC]

Additional file 3, Table S2. List of all observed *M. tuberculosis* H37Rv proteins in the lipid phase of Triton X-114 detergent, sorted by their Sanger IDs.

| Sanger ID | *Gene name* | Protein description | Functional group | GRAVY score | No. of TMH | References |
| --- | --- | --- | --- | --- | --- | --- |
| Rv0001 | *dnaA* | Chromosomal replication initiator protein | 2 | -0.385 | 0 | [3] |
| Rv0002 | *dnaN* | DNA polymerase III | 2 | 0.16 | 0 | [2] |
| Rv0006 | *gyrA* | DNA gyrase | 2 | -0.303 | 0 | [1, 10] |
| Rv0007 | *-* | Possible conserved membrane protein | 3 | -0.138 | 2 | [10] |
| Rv0008c | *-* | Possible membrane protein | 3 | -0.284 | 1 | [1, 3, 10] |
| Rv0009 | *ppiA* | Possible iron-regulated peptidyl-prolyl cis-trans isomerase a | 2 | -0.303 | 0 | [1-4, 6, 7] |
| Rv0010c | *-* | Possible conserved membrane protein | 3 | 0.279 | 2 | [3] |
| Rv0011c | *-* | Possible conserved transmembrane protein | 3 | 0.632 | 2 |  |
| Rv0014c | *pknB* | transmembrane serine/threonine-protein kinase b | 9 | -0.173 | 1 | [3] |
| Rv0016c | *pbpA* | Possible penicillin-binding protein | 3 | -0.085 | 1 | [6] |
| Rv0019c | *fhaB* | Conserved protein with fha domain | 9 | 0.117 | 1 | [1-3, 5, 6] |
| Rv0020c | *fhaA* | Conserved hypothetical protein with fha domain | 9 | -1.068 | 0 | [1, 3, 4, 6, 7] |
| Rv0023 | *-* | Possible transcriptional regulatory protein | 9 | -0.098 | 0 |  |
| Rv0036c | *-* | Conserved hypothetical protein | 10 | -0.051 | 0 | [1, 7] |
| Rv0037c | *-* | Possible conserved integral membrane protein | 3 | 0.84 | 11 |  |
| Rv0041 | *leuS* | Possible leucyl-tRNA synthetase | 2 | -0.317 | 0 | [1, 6] |
| Rv0042c | *-* | Possible transcriptional regulatory protein | 9 | -0.224 | 0 | [1] |
| Rv0046c | *ino1* | Myo-inositol-1-phosphate synthase | 7 | -0.099 | 0 | [1, 7] |
| Rv0047c | *-* | Conserved hypothetical protein | 10 | -0.543 | 0 |  |
| Rv0048c | *-* | Possible membrane protein | 3 | -0.129 | 1 | [1, 3, 5, 9, 10] |
| Rv0050 | *ponA1* | Possible bifunctional penicillin-binding protein | 3 | -0.126 | 0 | [5] |
| Rv0052 | *-* | Conserved hypothetical protein | 10 | 0.108 | 1 | [1, 3, 7] |
| Rv0053 | *rpsF* | Possible 30s ribosomal protein | 2 | -0.251 | 0 | [1, 3, 4, 7] |
| Rv0054 | *ssb* | Possible single-strand binding protein | 2 | -0.488 | 0 | [1, 2, 4, 7, 10] |
| Rv0055 | *rpsR1* | Possible 30s ribosomal protein | 2 | -0.614 | 0 | [1] |
| Rv0056 | *rplI* | Possible 50s ribosomal protein | 2 | -0.021 | 0 | [1, 6, 7, 10] |
| Rv0066c | *icd2* | Possible isocitrate dehydrogenase | 7 | -0.289 | 0 | [1, 6, 7] |
| Rv0069c | *sdaA* | Possible l-serine dehydratase | 7 | 0.064 | 0 | [6] |
| Rv0070c | *glyA2* | Possible serine hydroxymethyltransferase | 7 | -0.081 | 0 | [6] |
| Rv0072 | *-* | Possible glutamine-transport transmembrane protein abc transporter | 3 | 0.663 | 4 | [1, 10] |
| Rv0073 | *-* | Possible glutamine-transport ATP-binding protein abc transporter | 3 | 0.017 | 0 |  |
| Rv0074 | *-* | Conserved hypothetical protein | 10 | 0.05 | 0 | [6] |
| Rv0075 | *-* | Possible aminotransferase | 7 | 0.066 | 0 | [1, 2] |
| Rv0077c | *-* | Possible oxidoreductase | 7 | 0.138 | 0 |  |
| Rv0078 | *-* | Possible transcriptional regulatory protein | 9 | -0.05 | 0 | [1] |
| Rv0082 | *-* | Possible oxidoreductase | 7 | 0.377 | 0 |  |
| Rv0088 | *-* | Hypothetical protein | 10 | -0.365 | 0 | [1, 3, 6, 7] |
| Rv0093c | *-* | Possible conserved membrane protein | 3 | 0.163 | 3 | [6] |
| Rv0110 | *-* | Possible conserved integral membrane protein | 3 | 0.727 | 7 |  |
| Rv0119 | *fadD7* | Possible fatty-acid-CoA ligase | 1 | 0.077 | 0 | [1] |
| Rv0120c | *fusA2* | Possible elongation factor | 2 | -0.062 | 0 | [1, 6-8] |
| Rv0121c | *-* | Conserved hypothetical protein | 10 | -0.234 | 0 |  |
| Rv0125 | *pepA* | Possible serine protease | 7 | 0.182 | 1 | [1, 2, 4] |
| Rv0126 | *treS* | Trehalose synthase | 0 | -0.367 | 0 | [1] |
| Rv0127 | *-* | Conserved hypothetical protein | 10 | -0.183 | 0 | [1, 6] |
| Rv0129c | *fbpC* | Secreted antigen | 1 | -0.253 | 1 | [2-4, 7] |
| Rv0130 | *htdZ* | Possible 3-hydroxyl-thioester dehydratase | 7 | 0.091 | 0 |  |
| Rv0131c | *fadE1* | Possible acyl-CoA dehydrogenase | 1 | -0.291 | 0 | [6, 10] |
| Rv0133 | *-* | Gcn5-related n-acetyltransferase | 7 | -0.477 | 0 | [1] |
| Rv0134 | *ephF* | Possible epoxide hydrolase | 0 | -0.196 | 0 | [1, 10] |
| Rv0136 | *cyp138* | Possible cytochrome | 7 | -0.235 | 0 | [1] |
| Rv0141c | *-* | hypothetical protein | 10 | -0.232 | 0 | [8] |
| Rv0143c | *-* | Possible conserved transmembrane protein | 3 | 0.574 | 10 |  |
| Rv0144 | *-* | Possible transcriptional regulatory protein | 9 | -0.096 | 1 | [1, 6] |
| Rv0147 | *-* | Possible aldehyde dehydrogenase (NAD+) dependent | 7 | -0.144 | 0 | [1, 6, 8-10] |
| Rv0148 | *-* | Possible short-chain type dehydrogenase/reductase | 7 | 0.111 | 0 | [1, 3, 7, 10] |
| Rv0149 | *-* | Possible quinone oxidoreductase | 7 | 0.415 | 0 | [1, 8] |
| Rv0154c | *fadE2* | Possible acyl-CoA dehydrogenase | 1 | -0.276 | 0 | [1, 6] |
| Rv0155 | *pntAa* | Possible NAD(P) transhydrogenase | 7 | 0.137 | 0 | [1, 3, 7] |
| Rv0161 | *-* | Possible oxidoreductase | 7 | 0.081 | 0 |  |
| Rv0162c | *adhE1* | Possible zinc-type alcohol dehydrogenase | 7 | 0.328 | 0 | [1] |
| Rv0163 | *-* | Conserved hypothetical protein | 10 | 0.169 | 0 |  |
| Rv0167 | *yrbE1A* | Conserved hypothetical integral membrane protein | 0 | 1 | 6 | [1, 3] |
| Rv0168 | *yrbE1B* | Conserved hypothetical integral membrane protein | 0 | 0.772 | 5 |  |
| Rv0169 | *mce1A* | Mce-family protein | 0 | 0.071 | 1 | [6] |
| Rv0170 | *mce1B* | Mce-family protein | 0 | -0.06 | 1 | [1, 2] |
| Rv0171 | *mce1C* | Mce-family protein | 0 | -0.156 | 1 | [3] |
| Rv0172 | *mce1D* | Mce-family protein | 0 | -0.022 | 1 | [2, 10] |
| Rv0173 | *lprK* | Possible mce-family lipoprotein | 3 | -0.069 | 1 | [2, 10] |
| Rv0174 | *mce1F* | Mce-family protein | 0 | -0.086 | 1 | [2, 3, 6, 8] |
| Rv0176 | *-* | Possible conserved mce associated transmembrane protein | 3 | 0.045 | 3 | [1, 3] |
| Rv0177 | *-* | Possible conserved mce associated protein | 10 | -0.186 | 1 | [1, 6] |
| Rv0180c | *-* | Possible conserved transmembrane protein | 3 | 0.482 | 6 | [1, 3, 5, 10] |
| Rv0183 | *-* | Possible lysophospholipase | 7 | -0.028 | 0 | [1, 3, 4, 6-8] |
| Rv0186 | *bglS* | Possible beta-glucosidase | 7 | -0.087 | 0 | [6] |
| Rv0187 | *-* | Possible o-methyltransferase | 7 | 0.171 | 0 | [1, 6] |
| Rv0189c | *ilvD* | Possible dihydroxy-acid dehydratase | 7 | 0.155 | 0 | [1, 6, 8, 10] |
| Rv0190 | *-* | Conserved hypothetical protein | 10 | -0.164 | 0 |  |
| Rv0201c | *-* | Conserved hypothetical protein | 10 | -0.305 | 0 |  |
| Rv0205 | *-* | Possible conserved transmembrane protein | 3 | 1.058 | 8 | [3] |
| Rv0206c | *mmpL3* | Possible conserved transmembrane transport protein | 3 | 0.237 | 11 | [3, 6, 10] |
| Rv0211 | *pckA* | Possible iron-regulated phosphoenolpyruvate carboxykinase | 7 | -0.321 | 0 | [1, 2, 6-8, 10] |
| Rv0216 | *-* | Double hotdog hydratase | 7 | -0.016 | 0 | [1, 3, 5, 8] |
| Rv0218 | *-* | Possible conserved transmembrane protein | 3 | 0.446 | 5 |  |
| Rv0221 | *-* | Conserved hypothetical protein | 10 | -0.118 | 0 | [1] |
| Rv0222 | *echA1* | Possible enoyl-CoA hydratase | 1 | 0.106 | 0 | [1] |
| Rv0226c | *-* | Possible conserved transmembrane protein | 3 | 0.648 | 10 | [6] |
| Rv0227c | *-* | Possible conserved membrane protein | 3 | -0.41 | 2 | [1, 3, 5, 6, 9, 10] |
| Rv0229c | *-* | Possible conserved membrane protein with pin domain | 3 | -0.441 | 1 |  |
| Rv0230c | *php* | Possible phosphotriesterase | 1 | -0.097 | 0 |  |
| Rv0231 | *fadE4* | Possible acyl-CoA dehydrogenase | 1 | -0.155 | 0 | [1, 8, 10] |
| Rv0233 | *nrdB* | Possible ribonucleoside-diphosphate reductase | 2 | -0.372 | 0 | [6, 8] |
| Rv0234c | *gabD1* | Possible succinate-semialdehyde dehydrogenase [NADP+] dependent | 7 | 0.007 | 0 | [1, 6-8] |
| Rv0235c | *-* | Possible conserved transmembrane protein | 3 | 0.238 | 6 |  |
| Rv0237 | *lpqI* | Possible conserved lipoprotein | 3 | 0.123 | 0 | [2, 3, 5, 6, 10] |
| Rv0238 | *-* | Possible transcriptional regulatory protein | 9 | -0.052 | 0 | [1, 6] |
| Rv0241c | *-* | Double hotdog hydratase | 7 | 0.016 | 0 | [1, 9] |
| Rv0242c | *fabG4* | Possible 3-oxoacyl-[acyl-carrier protein] reductase | 1 | 0.069 | 0 | [1, 2, 4-7, 9] |
| Rv0243 | *fadA2* | Possible acetyl-CoA acyltransferase | 1 | -0.05 | 0 | [1, 3-6, 9, 10] |
| Rv0244c | *fadE5* | Possible acyl-CoA dehydrogenase | 1 | -0.012 | 0 | [1, 2, 6, 8, 10] |
| Rv0247c | *-* | Possible succinate dehydrogenase | 7 | -0.455 | 0 | [1, 3, 5, 7, 8] |
| Rv0248c | *-* | Possible succinate dehydrogenase | 7 | -0.332 | 0 | [1, 3, 5-7, 9, 10] |
| Rv0249c | *-* | Possible succinate dehydrogenase | 7 | 0.229 | 5 | [3] |
| Rv0250c | *-* | Conserved hypothetical protein | 10 | -0.435 | 0 | [1] |
| Rv0251c | *hsp* | Heat shock protein | 0 | -0.473 | 0 | [1, 3, 5, 9] |
| Rv0265c | *fecB2* | Possible periplasmic iron-transport lipoprotein | 3 | 0.033 | 0 | [1-3, 5] |
| Rv0266c | *oplA* | Possible 5-oxoprolinase | 7 | -0.162 | 0 | [1, 6, 10] |
| Rv0270 | *fadD2* | Possible fatty-acid-CoA ligase | 1 | -0.071 | 0 | [1, 3, 5-7, 9, 10] |
| Rv0271c | *fadE6* | Possible acyl-CoA dehydrogenase | 1 | 0.018 | 0 | [1, 6] |
| Rv0272c | *-* | Hypothetical protein | 10 | -0.262 | 0 |  |
| Rv0274 | *-* | Conserved hypothetical protein | 10 | -0.125 | 0 |  |
| Rv0281 | *-* | Conserved hypothetical protein. | 10 | -0.239 | 0 | [1, 3] |
| Rv0282 | *-* | Conserved hypothetical protein | 10 | -0.146 | 0 | [1, 6, 7, 10] |
| Rv0283 | *-* | Possible conserved membrane protein | 3 | 0.039 | 1 | [1, 2, 6, 10] |
| Rv0284 | *-* | Possible conserved membrane protein | 3 | -0.126 | 1 | [1, 6, 10] |
| Rv0285 | *PE5* | PE family protein | 6 | 0.665 | 0 | [2] |
| Rv0287 | *esxG* | Esat-6 like protein | 3 | 0.299 | 0 | [2, 7] |
| Rv0290 | *-* | Possible conserved transmembrane protein | 3 | 1.002 | 11 | [1, 3, 6, 10] |
| Rv0291 | *mycP3* | Possible membrane-anchored mycosin | 7 | 0.15 | 1 | [1, 2, 5, 10] |
| Rv0292 | *-* | Possible conserved transmembrane protein | 3 | -0.034 | 2 | [1, 10] |
| Rv0293c | *-* | Conserved hypothetical protein | 10 | 0.042 | 0 | [1] |
| Rv0296c | *atsG* | Possible sulfatase | 7 | -0.366 | 0 | [6] |
| Rv0301 | *-* | Conserved hypothetical protein with pin domain | 0 | -0.268 | 0 |  |
| Rv0307c | *-* | Hypothetical protein | 10 | 0.26 | 0 |  |
| Rv0308 | *-* | Possible conserved integral membrane protein | 3 | 0.67 | 6 | [3, 5] |
| Rv0309 | *-* | Possible conserved exported protein | 3 | 0.146 | 1 | [2] |
| Rv0310c | *-* | Conserved hypothetical protein | 10 | -0.281 | 0 |  |
| Rv0311 | *-* | Hypothetical protein | 10 | -0.022 | 0 |  |
| Rv0313 | *-* | Conserved hypothetical protein | 10 | -0.529 | 0 | [1, 3] |
| Rv0314c | *-* | Possible conserved membrane protein | 3 | 0.019 | 1 | [3] |
| Rv0315 | *-* | Possible beta-1,3-glucanase precursor | 7 | -0.351 | 0 | [2, 3] |
| Rv0316 | *-* | Possible muconolactone isomerase | 7 | -0.325 | 0 | [6] |
| Rv0319 | *pcp* | Possible pyrrolidone-carboxylate peptidase | 7 | 0.327 | 0 |  |
| Rv0321 | *dcd* | Possible deoxycytidine triphosphate deaminase | 7 | -0.245 | 0 | [1] |
| Rv0328 | *-* | Possible transcriptional regulatory protein | 9 | -0.01 | 0 | [6] |
| Rv0331 | *-* | Possible dehydrogenase/reductase | 7 | 0.128 | 0 |  |
| Rv0332 | *-* | Conserved hypothetical protein | 10 | -0.287 | 0 | [1, 6] |
| Rv0333 | *-* | Hypothetical protein | 10 | 0.082 | 0 |  |
| Rv0338c | *-* | Possible iron-sulfur-binding reductase | 7 | -0.107 | 6 | [1, 6, 10] |
| Rv0346c | *ansP2* | Possible l-asparagine permease | 3 | 0.714 | 12 |  |
| Rv0350 | *dnaK* | Possible chaperone protein | 0 | -0.368 | 0 | [1-3, 5-7] |
| Rv0351 | *grpE* | Possible grpe protein | 0 | -0.58 | 0 | [1, 7] |
| Rv0352 | *dnaJ1* | Possible chaperone protein | 0 | -0.448 | 0 | [1, 6] |
| Rv0356c | *-* | Conserved hypothetical protein | 10 | -0.112 | 0 |  |
| Rv0357c | *purA* | Possible adenylosuccinate synthetase | 7 | -0.137 | 0 | [10] |
| Rv0358 | *-* | Conserved hypothetical protein | 10 | -0.062 | 0 |  |
| Rv0359 | *-* | Possible conserved integral membrane protein | 3 | 0.816 | 6 |  |
| Rv0360c | *-* | Conserved hypothetical protein | 10 | -0.119 | 0 | [3] |
| Rv0361 | *-* | Possible conserved membrane protein | 3 | -0.477 | 1 | [3, 10] |
| Rv0363c | *fba* | Possible fructose-bisphosphate aldolase | 7 | -0.08 | 0 | [1, 2, 7] |
| Rv0364 | *-* | Possible conserved transmembrane protein | 3 | 0.759 | 5 |  |
| Rv0365c | *-* | Conserved hypothetical protein | 10 | -0.26 | 0 | [6, 10] |
| Rv0379 | *secE2* | Possible protein transport protein | 3 | -0.261 | 0 | [1, 3] |
| Rv0382c | *pyrE* | Possible orotate phosphoribosyltransferase | 7 | 0.068 | 0 | [1] |
| Rv0383c | *-* | Possible conserved secreted protein | 3 | -0.445 | 1 | [1, 6, 10] |
| Rv0384c | *clpB* | Possible endopeptidase ATP binding protein | 0 | -0.266 | 0 | [1, 5-7] |
| Rv0389 | *purT* | Possible phosphoribosylglycinamide formyltransferase | 7 | 0.197 | 0 | [6] |
| Rv0390 | *-* | Conserved hypothetical protein | 10 | -0.309 | 0 |  |
| Rv0391 | *metZ* | Possible o-succinylhomoserine sulfhydrylase | 7 | 0.063 | 0 |  |
| Rv0392c | *ndhA* | Possible membrane NADH dehydrogenase | 7 | 0.101 | 1 | [1, 6] |
| Rv0399c | *lpqK* | Possible conserved lipoprotein | 3 | -0.093 | 0 |  |
| Rv0400c | *fadE7* | Acyl-CoA dehydrogenase | 1 | -0.007 | 0 | [1, 7, 10] |
| Rv0402c | *mmpL1* | Possible conserved transmembrane transport protein | 3 | 0.214 | 12 | [2, 6] |
| Rv0406c | *-* | Beta lactamase like protein | 10 | -0.012 | 0 |  |
| Rv0407 | *fgd1* | Possible f420-dependent glucose-6-phosphate dehydrogenase | 7 | -0.247 | 0 | [1, 3, 7, 10] |
| Rv0410c | *pknG* | Serine/threonine-protein kinase | 9 | -0.193 | 0 | [6] |
| Rv0411c | *glnH* | Possible glutamine-binding lipoprotein | 3 | 0.023 | 0 | [2, 3] |
| Rv0412c | *-* | Possible conserved membrane protein | 3 | -0.035 | 4 | [5, 10] |
| Rv0414c | *thiE* | Possible thiamine-phosphate pyrophosphorylase | 7 | -0.054 | 0 |  |
| Rv0415 | *thiO* | Possible thiamine biosynthesis oxidoreductase | 7 | 0.014 | 0 |  |
| Rv0417 | *thiG* | Possible thiamin biosynthesis protein | 7 | 0.362 | 0 |  |
| Rv0418 | *lpqL* | Possible lipoprotein aminopeptidase | 3 | -0.057 | 1 | [1, 3, 5, 6, 10] |
| Rv0419 | *lpqM* | Possible lipoprotein peptidase | 3 | -0.019 | 1 | [3] |
| Rv0423c | *thiC* | Possible thiamine biosynthesis protein | 7 | -0.218 | 0 | [6] |
| Rv0426c | *-* | Possible transmembrane protein | 3 | 0.282 | 2 |  |
| Rv0431 | *-* | Putative tuberculin related peptide | 3 | 0.29 | 1 | [3, 5, 10] |
| Rv0432 | *sodC* | Possible periplasmic superoxide dismutase | 0 | -0.061 | 0 | [1, 3, 5, 9, 10] |
| Rv0437c | *psd* | Possible phosphatidylserine decarboxylase | 1 | 0.138 | 0 | [1, 10] |
| Rv0439c | *-* | Possible dehydrogenase/reductase | 7 | -0.231 | 0 | [1, 3] |
| Rv0440 | *groEL2* | 60 kDa chaperonin 2 | 0 | -0.091 | 0 | [1, 3, 5-7, 10] |
| Rv0443 | *-* | Conserved hypothetical protein | 10 | -0.174 | 0 | [1, 3] |
| Rv0444c | *-* | Conserved hypothetical protein | 10 | 0.062 | 0 | [1] |
| Rv0445c | *sigK* | Possible alteRNAtive RNA polymerase sigma factor | 2 | -0.39 | 0 | [1, 6, 10] |
| Rv0450c | *mmpL4* | Possible conserved transmembrane transport protein | 3 | 0.266 | 11 | [3, 6] |
| Rv0456c | *echA2* | Enoyl-CoA hydratase | 1 | -0.12 | 0 |  |
| Rv0458 | *-* | Possible aldehyde dehydrogenase | 7 | -0.018 | 0 | [1] |
| Rv0461 | *-* | Possible transmembrane protein | 3 | 0.483 | 3 |  |
| Rv0462 | *lpdC* | Dihydrolipoamide dehydrogenase | 7 | 0.104 | 0 | [1, 2, 6, 7, 9] |
| Rv0464c | *-* | Conserved hypothetical protein | 10 | -0.145 | 0 | [1, 3, 6, 10] |
| Rv0468 | *fadB2* | Possible 3-hydroxybutyryl-CoA dehydrogenase | 1 | 0.17 | 0 | [1, 3, 6, 7] |
| Rv0474 | *-* | Possible transcriptional regulatory protein | 9 | -0.464 | 0 | [6] |
| Rv0475 | *hbhA* | Iron-regulated heparin binding hemagglutinin | 3 | -0.593 | 0 | [1, 3, 6, 7, 9, 10] |
| Rv0476 | *-* | Possible conserved transmembrane protein | 3 | 1.083 | 2 |  |
| Rv0479c | *-* | Possible conserved membrane protein | 3 | -0.196 | 1 | [1, 5, 10] |
| Rv0480c | *-* | Possible amidohydrolase | 7 | 0.061 | 0 | [6] |
| Rv0484c | *-* | Possible short-chain type oxidoreductase | 7 | 0.129 | 0 | [1] |
| Rv0489 | *gpm1* | Possible phosphoglycerate mutase | 7 | -0.199 | 0 | [1] |
| Rv0492c | *-* | Possible oxidoreductase gmc-type | 7 | 0.04 | 0 | [6, 10] |
| Rv0493c | *-* | Conserved hypothetical protein | 10 | -0.257 | 0 | [1] |
| Rv0497 | *-* | Possible conserved transmembrane protein | 3 | -0.284 | 3 | [3] |
| Rv0498 | *-* | Conserved hypothetical protein | 10 | -0.158 | 0 |  |
| Rv0500 | *proC* | Possible pyrroline-5-carboxylate reductase | 7 | 0.392 | 0 | [1, 10] |
| Rv0500A | *-* | Conserved hypothetical protein | 10 | -0.133 | 0 |  |
| Rv0504c | *-* | Conserved hypothetical protein | 10 | -0.047 | 0 | [1] |
| Rv0506 | *mmpS2* | Possible conserved membrane protein | 3 | 0.309 | 1 | [2] |
| Rv0511 | *hemD* | Possible uroporphyrin-III c-methyltransferase | 7 | -0.035 | 0 | [6, 10] |
| Rv0512 | *hemB* | Possible delta-aminolevulinic acid dehydratase | 7 | 0.05 | 0 |  |
| Rv0513 | *-* | Possible conserved transmembrane protein | 3 | 0.018 | 2 | [3] |
| Rv0514 | *-* | Possible transmembrane protein | 3 | 0.393 | 2 |  |
| Rv0518 | *-* | Possible exported protein | 3 | -0.059 | 0 |  |
| Rv0523c | *-* | Conserved hypothetical protein | 10 | -0.298 | 0 | [1, 6, 9, 10] |
| Rv0524 | *hemL* | Possible glutamate-1-semialdehyde 2,1-aminomutase | 7 | 0.202 | 0 | [1] |
| Rv0526 | *-* | Possible thioredoxin protein | 7 | -0.135 | 0 | [1-3, 5, 10] |
| Rv0530 | *-* | Conserved hypothetical protein | 10 | -0.276 | 0 | [1] |
| Rv0531 | *-* | Possible conserved membrane protein | 3 | 0.43 | 2 | [5] |
| Rv0533c | *fabH* | 3-oxoacyl-[acyl-carrier-protein] synthase III | 1 | 0.084 | 0 |  |
| Rv0537c | *-* | Possible integral membrane protein | 3 | 0.551 | 12 |  |
| Rv0538 | *-* | Possible conserved membrane protein | 3 | -0.001 | 1 | [3] |
| Rv0543c | *-* | Conserved hypothetical protein | 10 | -0.186 | 0 |  |
| Rv0544c | *-* | Possible conserved transmembrane protein | 3 | 1.043 | 2 |  |
| Rv0546c | *-* | Conserved hypothetical protein | 10 | -0.278 | 0 |  |
| Rv0548c | *menB* | Possible naphthoate synthase | 7 | -0.304 | 0 | [1, 5] |
| Rv0549c | *-* | Conserved hypothetical protein with pin domain | 0 | 0.088 | 0 |  |
| Rv0552 | *-* | Conserved hypothetical protein | 10 | -0.055 | 0 | [6] |
| Rv0553 | *menC* | Possible muconate cycloisomerase | 7 | 0.356 | 0 | [1, 3] |
| Rv0554 | *bpoC* | Possible peroxidase | 0 | 0.056 | 0 |  |
| Rv0556 | *-* | Possible conserved transmembrane protein | 3 | 0.299 | 2 | [1] |
| Rv0559c | *-* | Possible conserved secreted protein | 3 | -0.187 | 0 | [2] |
| Rv0560c | *-* | Possible benzoquinone methyltransferase | 7 | 0.067 | 0 |  |
| Rv0562 | *grcC1* | Possible polyprenyl-diphosphate synthase | 7 | 0.011 | 0 | [1, 3] |
| Rv0563 | *htpX* | Possible protease transmembrane protein heat shock protein | 0 | 0.304 | 4 | [2, 3, 5, 10] |
| Rv0564c | *gpdA1* | Possible glycerol-3-phosphate dehydrogenase [NAD(P)+] | 1 | 0.072 | 0 |  |
| Rv0566c | *-* | Conserved hypothetical protein | 10 | -0.543 | 0 | [1, 6, 7] |
| Rv0576 | *-* | Possible transcriptional regulatory protein | 9 | -0.013 | 0 | [1, 3] |
| Rv0577 | *TB27.3* | Conserved hypothetical protein | 10 | -0.027 | 0 | [1, 7] |
| Rv0578c | *PE_PGRS7* | PE-PGRS family protein | 6 | -0.299 | 0 |  |
| Rv0580c | *-* | Conserved hypothetical protein | 10 | -0.061 | 0 | [3, 5-7, 9, 10] |
| Rv0583c | *lpqN* | Possible conserved lipoprotein | 3 | -0.158 | 0 | [1-3, 10] |
| Rv0586 | *-* | Possible transcriptional regulatory protein | 9 | -0.189 | 0 |  |
| Rv0592 | *mce2D* | Mce-family protein | 0 | -0.109 | 1 | [6] |
| Rv0598c | *-* | Conserved hypothetical protein with pin domain | 0 | 0.251 | 0 | [3] |
| Rv0604 | *lpqO* | Possible conserved lipoprotein | 3 | 0 | 0 | [1] |
| Rv0616A | *-* | Conserved hypothetical protein | 0 | -0.355 | 0 |  |
| Rv0617 | *-* | Conserved hypothetical protein with pin domain | 0 | 0.38 | 0 |  |
| Rv0624 | *-* | Conserved hypothetical protein with pin domain | 0 | -0.06 | 0 |  |
| Rv0625c | *-* | Possible conserved transmembrane protein | 3 | 0.593 | 5 |  |
| Rv0627 | *-* | Conserved hypothetical protein with pin domain | 0 | 0.288 | 0 |  |
| Rv0630c | *recB* | Possible exonuclease v (beta chain) | 2 | -0.166 | 0 | [6] |
| Rv0632c | *echA3* | Possible enoyl-CoA hydratase | 1 | 0.206 | 0 | [1, 6, 7, 10] |
| Rv0633c | *-* | Possible exported protein | 3 | -0.503 | 1 |  |
| Rv0634c | *-* | Possible glyoxalase II | 0 | -0.168 | 0 | [3] |
| Rv0635 | *-* | Conserved hypothetical protein | 10 | -0.174 | 0 | [1, 3] |
| Rv0636 | *-* | Single hotdog hydratase | 7 | 0.02 | 0 | [3, 6, 7] |
| Rv0638 | *secE1* | Possible preprotein translocase | 3 | -0.152 | 1 | [3, 10] |
| Rv0639 | *nusG* | Possible transcription antitermination protein | 2 | -0.18 | 0 | [1, 3, 7] |
| Rv0640 | *rplK* | Possible 50s ribosomal protein | 2 | -0.133 | 0 | [1, 6, 9] |
| Rv0641 | *rplA* | Possible 50s ribosomal protein | 2 | -0.2 | 0 | [1, 6, 7, 9] |
| Rv0642c | *mmaA4* | Methoxy mycolic acid synthase | 1 | -0.416 | 0 | [1, 3, 6, 7] |
| Rv0644c | *mmaA2* | Methoxy mycolic acid synthase | 1 | -0.231 | 0 | [1, 3] |
| Rv0645c | *mmaA1* | Methoxy mycolic acid synthase | 1 | -0.326 | 0 | [1, 3] |
| Rv0647c | *-* | Conserved hypothetical protein | 10 | -0.157 | 0 | [1, 6, 10] |
| Rv0651 | *rplJ* | Possible 50s ribosomal protein | 2 | 0.084 | 0 | [1, 3, 6, 7, 9] |
| Rv0652 | *rplL* | Possible 50s ribosomal protein | 2 | 0.18 | 0 | [1, 3, 5-7, 9] |
| Rv0654 | *-* | Possible dioxygenase | 7 | -0.295 | 0 |  |
| Rv0655 | *mkl* | Possible ribonucleotide-transport ATP-binding protein abc transporter | 3 | -0.177 | 0 | [1, 6] |
| Rv0667 | *rpoB* | DNA-directed RNA polymerase | 2 | -0.326 | 0 | [1, 3, 6, 10] |
| Rv0668 | *rpoC* | DNA-directed RNA polymerase | 2 | -0.363 | 0 | [1, 3, 6, 10] |
| Rv0669c | *-* | Possible hydrolase | 7 | -0.294 | 0 | [6, 10] |
| Rv0670 | *end* | Possible endonuclease | 2 | -0.167 | 0 |  |
| Rv0675 | *echA5* | Possible enoyl-CoA hydratase | 1 | 0.059 | 0 | [1] |
| Rv0676c | *mmpL5* | Possible conserved transmembrane transport protein | 3 | 0.324 | 12 | [6, 10] |
| Rv0677c | *mmpS5* | Possible conserved membrane protein | 3 | 0.213 | 1 | [2] |
| Rv0678 | *-* | Conserved hypothetical protein | 10 | -0.36 | 0 | [1] |
| Rv0679c | *-* | Conserved hypothetical threonine rich protein | 10 | -0.055 | 0 | [1, 3] |
| Rv0680c | *-* | Possible conserved transmembrane protein | 3 | 0.151 | 2 | [2, 3] |
| Rv0682 | *rpsL* | Possible 30s ribosomal protein | 2 | -0.814 | 0 |  |
| Rv0683 | *rpsG* | Possible 30s ribosomal protein | 2 | -0.606 | 0 | [1, 6, 9, 10] |
| Rv0684 | *fusA1* | Possible elongation factor g | 2 | -0.289 | 0 | [1, 7] |
| Rv0685 | *tuf* | Possible iron-regulated elongation factor | 2 | -0.288 | 0 | [1, 3, 6, 7, 9, 10] |
| Rv0686 | *-* | Possible membrane protein | 3 | 0.274 | 2 | [1, 10] |
| Rv0688 | *-* | Putative ferredoxin reductase | 7 | 0.048 | 0 |  |
| Rv0694 | *lldD1* | Possible l-lactate dehydrogenase | 7 | -0.003 | 0 | [6, 10] |
| Rv0700 | *rpsJ* | 30s ribosomal protein s10 | 2 | -0.326 | 0 | [1, 3, 6, 9] |
| Rv0702 | *rplD* | Possible 50s ribosomal protein | 2 | -0.427 | 0 | [1, 6, 9, 10] |
| Rv0703 | *rplW* | Possible 50s ribosomal protein | 2 | -0.285 | 0 | [1, 3, 6, 9, 10] |
| Rv0705 | *rpsS* | Possible 30s ribosomal protein | 2 | -0.811 | 0 | [1] |
| Rv0706 | *rplV* | Possible 50s ribosomal protein | 2 | -0.494 | 0 | [1, 6, 10] |
| Rv0707 | *rpsC* | Possible 30s ribosomal protein | 2 | -0.621 | 0 | [1, 6, 9, 10] |
| Rv0709 | *rpmC* | Possible 50s ribosomal protein | 2 | -0.748 | 0 | [1, 7] |
| Rv0710 | *rpsQ* | Possible 30s ribosomal protein | 2 | -0.665 | 0 | [1, 10] |
| Rv0711 | *atsA* | Possible arylsulfatase | 7 | -0.251 | 0 | [6] |
| Rv0712 | *-* | Conserved hypothetical protein | 10 | -0.306 | 0 |  |
| Rv0716 | *rplE* | Possible 50s ribosomal protein | 2 | -0.344 | 0 | [1, 3, 7, 9, 10] |
| Rv0718 | *rpsH* | Possible 30s ribosomal protein | 2 | -0.2 | 0 | [1, 6, 10] |
| Rv0719 | *rplF* | Possible 50s ribosomal protein | 2 | -0.367 | 0 | [1, 6, 9, 10] |
| Rv0720 | *rplR* | Possible 50s ribosomal protein | 2 | -0.329 | 0 | [1, 9, 10] |
| Rv0721 | *rpsE* | Possible 30s ribosomal protein | 2 | -0.171 | 0 | [1, 6, 7, 9, 10] |
| Rv0722 | *rpmD* | Possible 50s ribosomal protein | 2 | -0.44 | 0 | [10] |
| Rv0723 | *rplO* | Possible 50s ribosomal protein | 2 | -0.428 | 0 | [1, 10] |
| Rv0724 | *sppA* | Possible protease | 3 | -0.018 | 0 | [1, 6, 7, 10] |
| Rv0727c | *fucA* | Possible l-fuculose phosphate aldolase | 7 | 0.164 | 0 |  |
| Rv0730 | *-* | Gcn5-related n-acetyltransferase | 10 | -0.29 | 0 | [1] |
| Rv0731c | *-* | Conserved hypothetical protein | 10 | -0.289 | 0 | [1, 9] |
| Rv0732 | *secY* | Possible preprotein translocase | 3 | 0.635 | 9 | [2] |
| Rv0733 | *adk* | Possible adenylate kinase | 7 | -0.411 | 0 | [1, 7] |
| Rv0734 | *mapA* | Possible methionine aminopeptidase | 7 | 0.151 | 0 | [1, 3] |
| Rv0738 | *-* | Conserved hypothetical protein | 10 | -0.096 | 0 | [3] |
| Rv0744c | *-* | Possible transcriptional regulatory protein | 9 | -0.583 | 0 |  |
| Rv0753c | *mmsA* | Possible methylmalonate-semialdehyde dehydrogenase | 7 | -0.014 | 0 | [1, 5, 7] |
| Rv0756c | *-* | Hypothetical protein | 10 | 0.023 | 0 | [3] |
| Rv0757 | *phoP* | Possible two component system response transcriptional positive regulator | 9 | -0.191 | 0 | [1, 6, 10] |
| Rv0760c | *-* | Conserved hypothetical protein | 10 | -0.215 | 0 |  |
| Rv0761c | *adhB* | Possible zinc-containing alcohol dehydrogenase NAD dependent | 7 | 0.115 | 0 | [1, 3] |
| Rv0769 | *-* | Possible dehydrogenase/reductase | 7 | 0.259 | 0 | [1] |
| Rv0772 | *purD* | Possible phosphoribosylamine--glycine ligase | 7 | 0.262 | 0 |  |
| Rv0773c | *ggtA* | Possible bifunctional acylase | 7 | -0.011 | 0 |  |
| Rv0785 | *-* | Conserved hypothetical protein | 10 | -0.189 | 0 | [6] |
| Rv0786c | *-* | Conserved hypothetical protein | 10 | 0.189 | 0 |  |
| Rv0788 | *purQ* | Possible phosphoribosylformylglycinamidine synthase I | 7 | 0.223 | 0 | [1, 3] |
| Rv0791c | *-* | Conserved hypothetical protein | 10 | -0.153 | 0 |  |
| Rv0794c | *lpdB* | Possible oxidoreductase | 7 | 0.018 | 0 |  |
| Rv0798c | *cfp29* | 29 kDa antigen | 0 | -0.141 | 0 | [1, 5, 7, 9, 10] |
| Rv0799c | *-* | Conserved hypothetical protein | 10 | -0.079 | 0 | [10] |
| Rv0800 | *pepC* | Possible aminopeptidase | 7 | 0.003 | 0 | [6, 7] |
| Rv0801 | *-* | Conserved hypothetical protein | 10 | -0.123 | 0 | [1] |
| Rv0803 | *purL* | Phosphoribosylformylglycinamidine synthase II | 7 | 0.036 | 0 | [1, 3] |
| Rv0811c | *-* | Conserved hypothetical protein | 10 | -0.19 | 0 | [6] |
| Rv0812 | *pabC* | Possible amino acid aminotransferase | 7 | 0.035 | 0 | [1, 6] |
| Rv0813c | *-* | Conserved hypothetical protein | 10 | -0.244 | 0 | [1] |
| Rv0814c | *sseC2* | Conserved hypothetical protein | 7 | 0.161 | 0 |  |
| Rv0815c | *cysA2* | Possible thiosulfate sulfurtransferase | 7 | -0.494 | 0 | [1, 3, 6, 7] |
| Rv0817c | *-* | Possible conserved exported protein | 3 | 0.199 | 1 |  |
| Rv0821c | *phoY2* | Possible phosphate-transport system transcriptional regulatory protein | 3 | 0 | 0 | [1, 3] |
| Rv0827c | *-* | Possible transcriptional regulatory protein | 9 | -0.236 | 0 |  |
| Rv0830 | *-* | Conserved hypothetical protein | 10 | -0.081 | 0 | [1] |
| Rv0831c | *-* | Conserved hypothetical protein | 10 | -0.212 | 0 | [1, 3, 6] |
| Rv0838 | *lpqR* | Possible conserved lipoprotein | 3 | -0.022 | 0 | [2] |
| Rv0844c | *narL* | Possible nitrate/nitrite response transcriptional regulatory protein | 9 | 0.119 | 0 |  |
| Rv0846c | *-* | Possible oxidase | 7 | -0.169 | 0 |  |
| Rv0853c | *pdc* | Possible pyruvate or indole-3-pyruvate decarboxylase | 7 | 0.094 | 0 | [1] |
| Rv0854 | *-* | Conserved hypothetical protein | 10 | -0.41 | 0 |  |
| Rv0858c | *-* | Possible aminotransferase | 7 | 0.116 | 0 | [3] |
| Rv0859 | *fadA* | Possible acyl-CoA thiolase | 1 | 0.035 | 0 | [1] |
| Rv0860 | *fadB* | Possible fatty oxidation protein | 1 | -0.055 | 0 | [1, 6, 7, 10] |
| Rv0864 | *moaC2* | Possible molybdenum cofactor biosynthesis protein | 7 | 0.02 | 0 |  |
| Rv0865 | *mog* | Possible molybdopterin biosynthesis mog protein | 7 | 0.215 | 0 |  |
| Rv0866 | *moaE2* | Possible molybdenum cofactor biosynthesis protein | 7 | 0.173 | 0 |  |
| Rv0870c | *-* | Possible conserved integral membrane protein | 3 | 1.416 | 3 |  |
| Rv0871 | *cspB* | Possible cold shock-like protein | 0 | -0.67 | 0 | [1, 7] |
| Rv0873 | *fadE10* | Possible acyl-CoA dehydrogenase | 1 | -0.147 | 0 | [1, 6, 7, 9, 10] |
| Rv0879c | *-* | Possible conserved transmembrane protein | 3 | 0.654 | 2 |  |
| Rv0880 | *-* | Possible transcriptional regulatory protein | 9 | -0.085 | 0 |  |
| Rv0884c | *serC* | Possible phosphoserine aminotransferase | 7 | -0.03 | 0 | [1, 2, 7] |
| Rv0888 | *-* | Possible exported protein | 3 | -0.103 | 1 |  |
| Rv0889c | *citA* | Possible citrate synthase II | 7 | -0.013 | 0 | [6] |
| Rv0896 | *gltA2* | Possible citrate synthase I | 7 | -0.201 | 0 | [1, 6, 9, 10] |
| Rv0897c | *-* | Possible oxidoreductase | 7 | 0.105 | 0 |  |
| Rv0899 | *ompA* | Outer membrane protein | 3 | 0.035 | 1 | [3, 10] |
| Rv0901 | *-* | Possible conserved exported or membrane protein | 3 | -0.466 | 1 | [1, 3] |
| Rv0904c | *accD3* | Putative acetyl-coenzyme a carboxylase carboxyl transferase | 1 | 0.155 | 0 | [6] |
| Rv0905 | *echA6* | Possible enoyl-CoA hydratase | 1 | -0.031 | 0 | [1, 6, 7, 10] |
| Rv0906 | *-* | Conserved hypothetical protein | 10 | -0.034 | 0 | [1, 3, 5, 7, 10] |
| Rv0907 | *-* | Conserved hypothetical protein | 3 | -0.19 | 0 | [6, 9, 10] |
| Rv0908 | *ctpE* | Possible metal cation transporter ATPase p-type | 3 | 0.361 | 9 |  |
| Rv0911 | *-* | Conserved hypothetical protein | 10 | -0.096 | 0 |  |
| Rv0914c | *-* | Possible lipid carrier protein or keto acyl-CoA thiolase | 1 | -0.2 | 0 |  |
| Rv0918 | *-* | Conserved hypothetical protein | 10 | -0.252 | 0 |  |
| Rv0925c | *-* | Conserved hypothetical protein | 10 | -0.356 | 0 |  |
| Rv0927c | *-* | Possible short-chain type dehydrogenase/reductase | 7 | 0.41 | 0 | [1, 10] |
| Rv0928 | *pstS3* | Periplasmic phosphate-binding lipoprotein | 3 | -0.145 | 0 | [2, 3, 5, 10] |
| Rv0931c | *pknD* | Transmembrane serine/threonine-protein kinase d | 9 | -0.055 | 1 | [1, 6, 10] |
| Rv0932c | *pstS2* | Periplasmic phosphate-binding lipoprotein | 3 | -0.138 | 0 | [1-3, 5, 10] |
| Rv0933 | *pstB* | Phosphate-transport ATP-binding protein abc transporter | 3 | -0.127 | 0 | [1, 6, 10] |
| Rv0934 | *pstS1* | Periplasmic phosphate-binding lipoprotein | 3 | 0.066 | 0 | [1-3, 5-7, 9, 10] |
| Rv0935 | *pstC1* | Phosphate-transport integral membrane abc transporter | 3 | 0.917 | 8 |  |
| Rv0936 | *pstA2* | Phosphate-transport integral membrane abc transporter | 3 | 0.654 | 6 |  |
| Rv0946c | *pgi* | Possible glucose-6-phosphate isomerase | 7 | -0.082 | 0 | [1, 10] |
| Rv0951 | *sucC* | Possible succinyl-CoA synthetase | 7 | 0.02 | 0 | [3, 6] |
| Rv0952 | *sucD* | Possible succinyl-CoA synthetase | 7 | 0.213 | 0 | [1, 3, 6, 7] |
| Rv0953c | *-* | Possible oxidoreductase | 7 | -0.114 | 0 |  |
| Rv0954 | *-* | Possible conserved transmembrane protein | 3 | -0.015 | 4 | [3, 5, 10] |
| Rv0960 | *-* | Conserved hypothetical protein with pin domain | 0 | 0.15 | 0 |  |
| Rv0967 | *-* | Conserved hypothetical protein | 10 | -0.15 | 0 | [6, 7] |
| Rv0968 | *-* | Conserved hypothetical protein | 10 | 0.132 | 0 | [1, 3, 5, 7, 10] |
| Rv0969 | *ctpV* | Possible metal cation transporter p-type ATPase | 3 | 0.303 | 6 | [1, 10] |
| Rv0972c | *fadE12* | Possible acyl-CoA dehydrogenase | 1 | -0.044 | 0 | [1, 6] |
| Rv0979A | *rpmF* | Possible 50s ribosomal protein l32 | 2 | -0.789 | 0 | [10] |
| Rv0983 | *pepD* | Possible serine protease | 7 | 0.022 | 1 |  |
| Rv0984 | *moaB2* | Possible pterin-4-alpha-carbinolamine dehydratase | 7 | 0.412 | 0 | [1, 3, 7] |
| Rv0985c | *mscL* | Possible large-conductance ion mechanosensitive channel | 3 | 0.283 | 2 | [1, 10] |
| Rv0988 | *-* | Possible conserved exported protein | 3 | -0.194 | 1 |  |
| Rv0991c | *-* | Conserved hypothetical serine rich protein | 10 | -0.673 | 0 |  |
| Rv0999 | *-* | Hypothetical protein | 10 | 0.137 | 0 | [1-3, 10] |
| Rv1001 | *arcA* | Possible arginine deiminase | 7 | 0.052 | 0 |  |
| Rv1002c | *-* | Conserved membrane protein | 3 | 0.481 | 8 |  |
| Rv1004c | *-* | Possible membrane protein | 3 | 0.835 | 1 |  |
| Rv1006 | *-* | Hypothetical protein | 10 | -0.304 | 0 | [1, 3, 5, 6, 9, 10] |
| Rv1007c | *metS* | Possible methionyl-tRNA synthetase | 2 | -0.228 | 0 | [6] |
| Rv1008 | *tatD* | Possible deoxyribonuclease | 3 | -0.135 | 0 | [1, 6] |
| Rv1015c | *rplY* | Possible 50s ribosomal protein | 2 | -0.091 | 0 | [1, 6] |
| Rv1016c | *lpqT* | Possible conserved lipoprotein | 3 | 0.009 | 0 | [10] |
| Rv1017c | *prsA* | Possible ribose-phosphate pyrophosphokinase | 7 | -0.082 | 0 | [1, 6, 7] |
| Rv1018c | *glmU* | Possible UDP-n-acetylglucosamine pyrophosphorylase | 3 | -0.021 | 0 |  |
| Rv1019 | *-* | Possible transcriptional regulatory protein | 9 | -0.155 | 0 | [3] |
| Rv1022 | *lpqU* | Possible conserved lipoprotein | 3 | -0.077 | 1 | [3] |
| Rv1023 | *eno* | Possible enolase | 7 | 0.015 | 0 | [1, 3, 5, 9] |
| Rv1024 | *-* | Possible conserved membrane protein | 3 | -0.618 | 0 |  |
| Rv1025 | *-* | Conserved hypothetical protein | 10 | -0.142 | 0 |  |
| Rv1030 | *kdpB* | Possible potassium-transporting p-type ATPase b chain | 3 | 0.358 | 7 |  |
| Rv1031 | *kdpC* | Possible potassium-transporting ATPase c chain | 3 | 0.062 | 1 |  |
| Rv1056 | *-* | Conserved hypothetical protein | 10 | -0.382 | 0 | [1] |
| Rv1059 | *-* | Conserved hypothetical protein | 10 | 0.28 | 0 |  |
| Rv1060 | *-* | Hypothetical protein | 10 | 0.178 | 0 | [1, 3, 10] |
| Rv1061 | *-* | Conserved hypothetical protein | 10 | -0.338 | 0 | [1] |
| Rv1069c | *-* | Conserved hypothetical protein | 10 | 0.029 | 5 | [6, 10] |
| Rv1070c | *echA8* | Possible enoyl-CoA hydratase | 1 | 0.066 | 0 | [1, 7] |
| Rv1071c | *echA9* | Possible enoyl-CoA hydratase | 1 | 0.132 | 0 | [3, 6] |
| Rv1072 | *-* | Possible conserved transmembrane protein | 3 | 0.801 | 7 |  |
| Rv1074c | *fadA3* | Possible beta-ketoacyl CoA thiolase | 1 | -0.11 | 0 | [1, 2, 7] |
| Rv1077 | *cysM2* | Possible cystathionine beta-synthase | 7 | -0.022 | 0 | [1, 2, 7] |
| Rv1078 | *pra* | Possible proline-rich antigen homolog | 10 | 0.085 | 2 | [1, 3, 5] |
| Rv1079 | *metB* | Possible cystathionine gamma-synthase | 7 | 0.001 | 0 | [1] |
| Rv1080c | *greA* | Possible transcription elongation factor | 2 | -0.632 | 0 | [1, 6, 7] |
| Rv1093 | *glyA1* | Possible serine hydroxymethyltransferase 1 | 7 | 0.045 | 0 | [1, 3, 5-7] |
| Rv1094 | *desA2* | Possible acyl-[acyl-carrier protein] desaturase | 1 | -0.189 | 0 | [1, 3, 6, 10] |
| Rv1096 | *-* | Possible glycosyl hydrolase | 7 | -0.202 | 1 |  |
| Rv1097c | *-* | Possible membrane glycine and proline rich protein | 3 | -0.088 | 1 | [3, 5, 6, 10] |
| Rv1098c | *fum* | Possible fumarase | 7 | 0.021 | 0 | [1-3, 6, 7, 10] |
| Rv1099c | *-* | Conserved hypothetical protein | 10 | -0.017 | 0 | [1, 6, 7] |
| Rv1100 | *-* | Conserved hypothetical protein | 10 | -0.297 | 1 |  |
| Rv1101c | *-* | Conserved membrane protein | 3 | 0.842 | 8 | [3, 10] |
| Rv1106c | *-* | 3-beta-hydroxysteroid dehydrogenase | 7 | -0.054 | 0 | [1, 7, 10] |
| Rv1109c | *-* | Conserved hypothetical protein | 10 | -0.268 | 0 | [1, 6, 7] |
| Rv1110 | *lytB2* | Possible lytb-related protein | 3 | -0.078 | 0 |  |
| Rv1117 | *-* | Conserved hypothetical protein | 10 | -0.262 | 0 |  |
| Rv1121 | *zwf1* | Possible glucose-6-phosphate 1-dehydrogenase | 7 | -0.194 | 0 | [1, 3] |
| Rv1122 | *gnd2* | Possible 6-phosphogluconate dehydrogenase | 7 | -0.165 | 0 | [1, 6] |
| Rv1124 | *ephC* | Possible epoxide hydrolase | 0 | -0.183 | 0 | [1, 6, 9] |
| Rv1132 | *-* | Conserved membrane protein | 3 | 0.445 | 8 | [6] |
| Rv1133c | *metE* | Possible5-methyltetrahydropteroyltriglutamate | 7 | -0.019 | 0 | [1, 6, 7] |
| Rv1143 | *mcr* | Possible alpha-methylacyl-CoA racemase | 1 | -0.112 | 0 |  |
| Rv1144 | *-* | Possible short-chain type dehydrogenase/reductase | 7 | 0.296 | 0 | [1, 7] |
| Rv1147 | *-* | Conserved hypothetical protein | 10 | 0.071 | 0 |  |
| Rv1152 | *-* | Possible transcriptional regulatory protein | 9 | -0.12 | 0 |  |
| Rv1155 | *-* | Conserved hypothetical protein | 10 | -0.283 | 0 |  |
| Rv1156 | *-* | Conserved hypothetical protein | 10 | -0.192 | 0 |  |
| Rv1157c | *-* | Conserved hypothetical ala-, pro-rich protein | 10 | 0.079 | 0 |  |
| Rv1161 | *narG* | Possible respiratory nitrate reductase | 7 | -0.363 | 0 | [1, 2, 6, 10] |
| Rv1162 | *narH* | Possible respiratory nitrate reductase | 7 | -0.298 | 0 | [6] |
| Rv1164 | *narI* | Possible respiratory nitrate reductase | 7 | 0.446 | 5 |  |
| Rv1166 | *lpqW* | Possible conserved lipoprotein | 3 | 0.027 | 0 | [2, 3, 5] |
| Rv1169c | *lipX* | PE family protein | 6 | -0.015 | 0 |  |
| Rv1170 | *mshB* | N-acetyl-1-d-myo-inosityl-2-amino-2-deoxy-alpha-d-glucopyranoside deacetylase | 7 | -0.084 | 0 | [1] |
| Rv1175c | *fadH* | Possible NADph dependent 2,4-dienoyl-CoA reductase | 1 | -0.112 | 0 | [1, 6] |
| Rv1177 | *fdxC* | Possible ferredoxin | 7 | -0.233 | 0 |  |
| Rv1178 | *-* | Possible aminotransferase | 7 | 0.24 | 0 | [1, 3] |
| Rv1179c | *-* | Hypothetical protein | 10 | -0.045 | 0 | [1, 6] |
| Rv1181 | *pks4* | Possible polyketide beta-ketoacyl synthase | 1 | 0.033 | 0 | [1, 6] |
| Rv1183 | *mmpL10* | Possible conserved transmembrane transport protein | 3 | 0.302 | 11 | [2, 6] |
| Rv1184c | *-* | Possible exported protein | 3 | -0.108 | 1 |  |
| Rv1188 | *-* | Possible proline dehydrogenase | 7 | -0.164 | 0 | [6] |
| Rv1191 | *-* | Conserved hypothetical protein | 10 | 0.034 | 0 | [1, 3] |
| Rv1192 | *-* | Hypothetical protein | 10 | -0.155 | 0 | [1] |
| Rv1194c | *-* | Conserved hypothetical protein | 10 | -0.2 | 0 | [1, 10] |
| Rv1196 | *PPE18* | PPE family protein | 6 | 0.24 | 0 |  |
| Rv1198 | *esxL* | Putative esat-6 like protein | 3 | -0.105 | 0 | [1-3, 6] |
| Rv1201c | *-* | Possible transferase | 7 | 0.168 | 0 | [1, 3, 7] |
| Rv1202 | *dapE* | Possible succinyl-diaminopimelate desuccinylase | 7 | 0.004 | 0 |  |
| Rv1206 | *fadD6* | Possible fatty-acid-CoA ligase | 1 | -0.046 | 0 | [1, 10] |
| Rv1209 | *-* | Conserved hypothetical protein | 10 | 0.239 | 1 | [1, 3] |
| Rv1213 | *glgC* | Possible glucose-1-phosphate adenylyltransferase | 7 | -0.099 | 0 | [6] |
| Rv1215c | *-* | Conserved hypothetical protein | 10 | -0.418 | 0 | [6, 10] |
| Rv1216c | *-* | Possible conserved integral membrane protein | 3 | 0.69 | 5 |  |
| Rv1217c | *-* | Possible tetronasin-transport integral membrane protein abc transporter | 3 | 0.715 | 12 |  |
| Rv1218c | *-* | Possible tetronasin-transport ATP-binding protein abc transporter | 3 | -0.122 | 0 | [1, 6, 7] |
| Rv1220c | *-* | Possible methyltransferase | 7 | 0.161 | 0 | [1] |
| Rv1223 | *htrA* | Possible serine protease | 7 | -0.127 | 1 | [5, 6, 10] |
| Rv1226c | *-* | Possible transmembrane protein | 3 | 0.311 | 5 | [6] |
| Rv1227c | *-* | Possible transmembrane protein | 3 | 0.106 | 2 |  |
| Rv1229c | *mrp* | Possible mrp-related protein | 7 | 0.079 | 0 | [1, 7] |
| Rv1231c | *-* | Possible membrane protein | 3 | -0.342 | 2 | [6] |
| Rv1234 | *-* | Possible transmembrane protein | 3 | 0.202 | 2 | [1, 10] |
| Rv1235 | *lpqY* | Possible sugar-binding lipoprotein | 3 | -0.004 | 0 | [3] |
| Rv1236 | *sugA* | Possible sugar-transport integral membrane protein abc transporter | 3 | 0.744 | 5 |  |
| Rv1237 | *sugB* | Possible sugar-transport integral membrane protein abc transporter | 3 | 0.916 | 6 |  |
| Rv1238 | *sugC* | Possible sugar-transport ATP-binding protein abc transporter | 3 | -0.046 | 0 |  |
| Rv1239c | *corA* | Possible magnesium and cobalt transport transmembrane protein | 3 | -0.089 | 2 | [3, 10] |
| Rv1240 | *mdh* | Possible malate dehydrogenase | 7 | 0.096 | 0 | [1, 6, 7] |
| Rv1242 | *-* | Conserved hypothetical protein with pin domain | 0 | 0.069 | 0 | [1] |
| Rv1244 | *lpqZ* | Possible lipoprotein | 3 | 0.269 | 0 | [3, 6] |
| Rv1245c | *-* | Possible short-chain type dehydrogenase/reductase | 7 | 0.101 | 0 | [1, 6, 7, 9, 10] |
| Rv1247c | *-* | Conserved hypothetical protein | 0 | -0.154 | 0 |  |
| Rv1252c | *lprE* | Possible lipoprotein | 3 | -0.132 | 0 | [2] |
| Rv1254 | *-* | Possible acyltransferase | 7 | 0.673 | 10 |  |
| Rv1257c | *-* | Possible oxidoreductase | 7 | 0.204 | 0 | [6] |
| Rv1261c | *-* | Conserved hypothetical protein | 10 | -0.666 | 0 | [1, 3, 9] |
| Rv1263 | *amiB2* | Possible amidase | 7 | 0.001 | 0 | [10] |
| Rv1265 | *-* | Hypothetical protein | 10 | -0.464 | 0 | [1] |
| Rv1269c | *-* | Conserved Possible secreted protein | 3 | 0.212 | 0 | [1, 2] |
| Rv1270c | *lprA* | Possible lipoprotein | 3 | 0.014 | 1 | [1-3, 5, 6, 10] |
| Rv1272c | *-* | Possible drugs-transport transmembrane ATP-binding protein abc transporter | 3 | 0.152 | 3 |  |
| Rv1273c | *-* | Possible drugs-transport transmembrane ATP-binding protein abc transporter | 3 | 0.402 | 6 |  |
| Rv1274 | *lprB* | Possible lipoprotein | 3 | -0.193 | 0 | [3] |
| Rv1275 | *lprC* | Possible lipoprotein | 3 | 0.131 | 0 | [1, 3, 5, 6] |
| Rv1279 | *-* | Possible dehydrogenase | 7 | -0.143 | 0 | [1, 6, 10] |
| Rv1280c | *oppA* | Possible periplasmic oligopeptide-binding lipoprotein | 3 | -0.146 | 1 | [5, 6, 9, 10] |
| Rv1281c | *oppD* | Possible oligopeptide-transport ATP-binding protein abc transporter | 3 | 0.077 | 0 | [6, 7] |
| Rv1284 | *canA* | Beta-carbonic anhydrase | 7 | -0.162 | 0 | [1, 7] |
| Rv1288 | *-* | Conserved hypothetical protein | 10 | 0.012 | 0 | [3] |
| Rv1292 | *argS* | Possible arginyl-tRNA synthetase | 2 | -0.147 | 0 | [3, 6] |
| Rv1293 | *lysA* | Possible diaminopimelate decarboxylase | 7 | 0.141 | 0 | [6] |
| Rv1295 | *thrC* | Possible threonine synthase | 7 | 0.175 | 0 | [1, 6, 7] |
| Rv1297 | *rho* | Possible transcription termination factor rho homolog | 2 | -0.712 | 0 | [1, 3, 6, 7] |
| Rv1299 | *prfA* | Possible peptide chain release factor 1 | 2 | -0.406 | 0 |  |
| Rv1301 | *-* | Conserved hypothetical protein | 10 | 0.193 | 0 | [3] |
| Rv1302 | *rfe* | Possible undecapaprenyl-phosphate alpha-n-acetylglucosaminyltransferase | 3 | 0.848 | 11 | [6] |
| Rv1304 | *ATPB* | Possible ATP synthase a chain | 7 | 0.818 | 5 | [1, 3, 10] |
| Rv1306 | *ATPF* | Possible ATP synthase b chain | 7 | 0.04 | 1 | [1, 3, 5, 10] |
| Rv1307 | *ATPH* | Possible ATP synthase delta chain | 7 | 0.027 | 1 | [1, 3, 5, 6, 9, 10] |
| Rv1308 | *ATPA* | Possible ATP synthase alpha chain | 7 | -0.207 | 0 | [1, 3, 6, 7, 10] |
| Rv1309 | *ATPG* | Possible ATP synthase gamma chain | 7 | -0.267 | 0 | [1, 3, 6, 9, 10] |
| Rv1310 | *ATPD* | Possible ATP synthase beta chain | 7 | -0.168 | 0 | [1-3, 6, 9, 10] |
| Rv1311 | *ATPC* | Possible ATP synthase epsilon chain | 7 | -0.061 | 0 | [1, 9] |
| Rv1314c | *-* | Conserved hypothetical protein | 10 | -0.093 | 0 |  |
| Rv1322A | *-* | Conserved hypothetical protein | 10 | 0.051 | 0 |  |
| Rv1323 | *fadA4* | Possible acetyl-CoA acetyltransferase | 1 | 0.139 | 0 | [1, 2, 7] |
| Rv1324 | *-* | Possible thioredoxin | 7 | 0 | 0 | [1, 6, 7] |
| Rv1325c | *PE_PGRS24* | PE-PGRS family protein | 6 | 0.179 | 0 |  |
| Rv1327c | *glgE* | Possible glucanase | 7 | -0.284 | 0 | [1, 6] |
| Rv1328 | *glgP* | Possible glycogen phosphorylase | 7 | -0.201 | 0 | [1, 6, 7, 10] |
| Rv1336 | *cysM* | Possible cysteine synthase b | 7 | -0.065 | 0 |  |
| Rv1339 | *-* | Conserved hypothetical protein | 10 | 0 | 0 | [1] |
| Rv1340 | *rphA* | Possible ribonuclease | 2 | -0.025 | 0 | [1] |
| Rv1341 | *-* | Conserved hypothetical protein | 10 | 0.064 | 0 | [3] |
| Rv1342c | *-* | Conserved membrane protein | 3 | 0.584 | 3 | [10] |
| Rv1343c | *lprD* | Possible conserved lipoprotein | 3 | -0.39 | 2 |  |
| Rv1350 | *fabG2* | Possible 3-oxoacyl-[acyl-carrier protein] reductase | 1 | 0.203 | 0 |  |
| Rv1363c | *-* | Possible membrane protein | 3 | -0.416 | 1 |  |
| Rv1368 | *lprF* | Possible conserved lipoprotein | 3 | -0.032 | 1 | [1, 3, 5, 6, 10] |
| Rv1377c | *-* | Putative transferase | 7 | -0.332 | 0 |  |
| Rv1380 | *pyrB* | Possible aspartate carbamoyltransferase | 7 | 0.08 | 0 | [1, 6] |
| Rv1381 | *pyrC* | Possible dihydroorotase | 7 | 0.091 | 0 |  |
| Rv1382 | *-* | Possible export or membrane protein | 3 | 0.049 | 1 | [2] |
| Rv1383 | *carA* | Possible carbamoyl-phosphate synthase small chain | 7 | -0.071 | 0 | [1] |
| Rv1384 | *carB* | Possible carbamoyl-phosphate synthase large chain | 7 | 0.004 | 0 | [1, 6] |
| Rv1385 | *pyrF* | Possible orotidine 5'-phosphate decarboxylase | 7 | 0.402 | 0 |  |
| Rv1386 | *PE15* | PE family protein | 6 | 0.597 | 0 | [2] |
| Rv1387 | *PPE20* | PPE family protein | 6 | 0.171 | 0 |  |
| Rv1388 | *mihF* | Putative integration host factor | 2 | -0.539 | 0 | [1, 6] |
| Rv1389 | *gmk* | Possible guanylate kinase | 7 | -0.041 | 0 |  |
| Rv1390 | *rpoZ* | Possible DNA-directed RNA polymerase | 2 | -0.207 | 0 | [3] |
| Rv1392 | *metK* | Possible s-adenosylmethionine synthetase | 7 | -0.106 | 0 | [1, 6, 7] |
| Rv1397c | *-* | Conserved hypothetical protein with pin domain | 0 | -0.008 | 0 | [1, 6, 9, 10] |
| Rv1404 | *-* | Possible transcriptional regulatory protein | 9 | -0.05 | 0 | [1] |
| Rv1409 | *ribG* | Possible bifunctional riboflavin biosynthesis protein | 7 | 0.088 | 0 | [1, 3, 6] |
| Rv1410c | *P55* | Aminoglycosides/tetracycline-transport integral membrane protein | 3 | 0.757 | 14 | [3, 10] |
| Rv1411c | *lprG* | Possible conserved lipoprotein | 3 | -0.127 | 0 | [1, 3, 5, 6, 9, 10] |
| Rv1412 | *ribC* | Possible riboflavin synthase alpha chain | 7 | 0.095 | 0 | [1] |
| Rv1416 | *ribH* | Possible riboflavin synthase beta chain | 7 | 0.21 | 0 | [1] |
| Rv1417 | *-* | Possible conserved membrane protein | 3 | 0.512 | 2 | [1] |
| Rv1418 | *lprH* | Possible lipoprotein | 3 | -0.003 | 1 |  |
| Rv1421 | *-* | Conserved hypothetical protein | 10 | -0.179 | 0 | [1, 6] |
| Rv1424c | *-* | Possible membrane protein | 3 | -0.148 | 0 | [6] |
| Rv1431 | *-* | Conserved membrane protein | 3 | 0.305 | 8 |  |
| Rv1436 | *gap* | Possible glyceraldehyde 3-phosphate dehydrogenase | 7 | 0.024 | 0 | [1-3, 6, 7, 10] |
| Rv1437 | *pgk* | Possible phosphoglycerate kinase | 7 | 0.217 | 0 | [1-3] |
| Rv1438 | *tpi* | Possible triosephosphate isomerase | 7 | 0.091 | 0 | [1, 2, 6, 7] |
| Rv1439c | *-* | Hypothetical protein | 10 | -0.077 | 0 |  |
| Rv1443c | *-* | Hypothetical protein | 10 | -0.303 | 0 | [1, 10] |
| Rv1444c | *-* | Hypothetical protein | 10 | -0.23 | 0 |  |
| Rv1445c | *devB* | Possible 6-phosphogluconolactonase | 7 | 0.028 | 0 | [1] |
| Rv1448c | *tal* | Possible transaldolase | 7 | -0.193 | 0 | [2, 6] |
| Rv1449c | *tkt* | Possible transketolase | 7 | -0.172 | 0 | [1-3, 6, 7] |
| Rv1454c | *qor* | Possible quinone reductase | 7 | 0.166 | 0 | [1, 2, 6, 7] |
| Rv1455 | *-* | Conserved hypothetical protein | 10 | -0.169 | 0 | [1] |
| Rv1458c | *-* | Possible unidentified antibiotic-transport ATP-binding protein abc transporter | 3 | 0.005 | 0 | [1] |
| Rv1459c | *-* | Possible conserved integral membrane protein | 3 | 0.63 | 10 |  |
| Rv1464 | *csd* | Possible cysteine desulfurase | 7 | 0.022 | 0 | [1, 3, 7] |
| Rv1465 | *-* | Possible nitrogen fixation related protein | 7 | -0.22 | 0 |  |
| Rv1466 | *-* | Conserved hypothetical protein | 10 | -0.004 | 0 | [3] |
| Rv1467c | *fadE15* | Possible acyl-CoA dehydrogenase | 1 | -0.016 | 0 | [1, 3, 6, 10] |
| Rv1468c | *PE_PGRS29* | PE-PGRS family protein | 6 | 0.199 | 0 |  |
| Rv1474c | *-* | Possible transcriptional regulatory protein | 9 | -0.452 | 0 | [6] |
| Rv1475c | *acn* | Possible iron-regulated aconitate hydratase | 7 | -0.251 | 0 | [1, 2, 6, 7] |
| Rv1476 | *-* | Possible membrane protein | 3 | 0.022 | 1 | [1, 3, 5] |
| Rv1479 | *moxR1* | Possible transcriptional regulatory protein | 9 | 0.051 | 0 | [1, 3, 5-7, 10] |
| Rv1481 | *-* | Possible membrane protein | 3 | 0.207 | 3 | [1, 3, 5, 10] |
| Rv1483 | *fabG1* | 3-oxoacyl-[acyl-carrier protein] reductase | 1 | 0.036 | 0 | [1, 3] |
| Rv1484 | *inhA* | NADH-dependent enoyl-[acyl-carrier-protein] reductase | 1 | 0.152 | 0 | [1, 3, 7, 10] |
| Rv1487 | *-* | Conserved membrane protein | 3 | 0.754 | 2 | [5] |
| Rv1488 | *-* | Possible exported conserved protein | 3 | -0.095 | 1 | [1-3, 5, 6, 9, 10] |
| Rv1489 | *-* | Conserved hypothetical protein | 10 | 0.814 | 2 | [10] |
| Rv1495 | *-* | Conserved hypothetical protein | 0 | -0.212 | 0 |  |
| Rv1498A | *-* | Conserved hypothetical protein | 10 | 0.017 | 0 | [2] |
| Rv1508c | *-* | Possible membrane protein | 3 | 0.5 | 10 | [6] |
| Rv1511 | *gmdA* | GDP-d-mannose dehydratase | 7 | -0.316 | 0 | [1] |
| Rv1512 | *epiA* | Possible nucleotide-sugar epimerase | 7 | -0.025 | 0 |  |
| Rv1513 | *-* | Conserved hypothetical protein | 10 | -0.17 | 0 | [1, 10] |
| Rv1530 | *adh* | Possible alcohol dehydrogenase | 7 | 0.239 | 0 |  |
| Rv1531 | *-* | Conserved hypothetical protein | 10 | 0.051 | 0 |  |
| Rv1533 | *-* | Conserved hypothetical protein | 10 | -0.01 | 0 |  |
| Rv1535 | *-* | Hypothetical protein | 10 | 0.197 | 0 |  |
| Rv1536 | *ileS* | Isoleucyl-tRNA synthetase | 2 | -0.265 | 0 | [1, 6] |
| Rv1538c | *ansA* | Possible l-aparaginase | 7 | 0.287 | 0 | [3] |
| Rv1542c | *glbN* | Possible hemoglobin | 7 | 0.154 | 0 |  |
| Rv1543 | *-* | Possible fatty acyl-CoA reductase | 1 | -0.084 | 0 | [1, 3, 6, 7, 10] |
| Rv1544 | *-* | Possible ketoacyl reductase | 1 | 0.184 | 0 | [1, 3, 7, 10] |
| Rv1546 | *-* | Conserved hypothetical protein | 10 | -0.211 | 0 | [1, 3] |
| Rv1552 | *frdA* | Possible fumarate reductase | 7 | -0.23 | 0 | [6] |
| Rv1553 | *frdB* | Possible fumarate reductase | 7 | -0.064 | 0 |  |
| Rv1558 | *-* | Conserved hypothetical protein | 10 | -0.476 | 0 | [1, 3, 7] |
| Rv1561 | *-* | Conserved hypothetical protein with pin domain | 0 | 0.199 | 0 |  |
| Rv1563c | *treY* | Maltooligosyltrehalose synthase | 0 | -0.021 | 0 |  |
| Rv1566c | *-* | Possible invasin protein | 0 | -0.111 | 1 |  |
| Rv1568 | *bioA* | Possible adenosylmethionine-8-amino-7-oxononanoate aminotransferase | 7 | 0.306 | 0 |  |
| Rv1571 | *-* | Conserved hypothetical protein | 10 | 0.133 | 0 |  |
| Rv1593c | *-* | Conserved hypothetical protein | 10 | -0.227 | 0 | [1] |
| Rv1594 | *NADA* | Possible quinolinate synthetase | 7 | -0.042 | 0 |  |
| Rv1596 | *NADC* | Possible nicotinate-nucleotide pyrophosphatase | 7 | 0.1 | 0 | [1, 3, 7] |
| Rv1600 | *hisC1* | Possible histidinol-phosphate aminotransferase | 7 | 0.067 | 0 | [6] |
| Rv1603 | *hisA* | Possible phosphoribosylformimino-5-aminoimidazole | 7 | 0.045 | 0 | [3] |
| Rv1606 | *hisI* | Possible phosphoribosyl-amp 1,6 cyclohydrolase | 7 | -0.279 | 0 |  |
| Rv1608c | *bcpB* | Possible peroxidoxin | 0 | -0.118 | 0 | [1] |
| Rv1609 | *trpE* | Possible anthranilate synthase component I | 7 | -0.143 | 0 | [1] |
| Rv1610 | *-* | Possible conserved membrane protein | 3 | 0.338 | 4 |  |
| Rv1611 | *trpC* | Possible indole-3-glycerol phosphate synthase | 7 | 0.246 | 0 | [1, 3, 6] |
| Rv1612 | *trpB* | Possible tryptophan synthase, beta subunit | 7 | -0.078 | 0 |  |
| Rv1613 | *trpA* | Possible tryptophan synthase, alpha subunit | 7 | 0.3 | 0 | [1, 3] |
| Rv1614 | *lgt* | Possible prolipoprotein diacylglyceryl transferases | 3 | 0.04 | 6 | [1, 10] |
| Rv1615 | *-* | Possible hypothetical membrane protein | 3 | 0.048 | 2 |  |
| Rv1617 | *pykA* | Possible pyruvate kinase | 7 | 0.013 | 0 | [1, 6] |
| Rv1622c | *cydB* | Possible integral membrane cytochrome d ubiquinol oxidase | 7 | 0.718 | 8 |  |
| Rv1623c | *cydA* | Possible integral membrane cytochrome d ubiquinol oxidase | 7 | 0.425 | 9 | [3] |
| Rv1624c | *-* | Possible conserved membrane protein | 3 | 1.354 | 6 |  |
| Rv1626 | *-* | Possible two-component system transcriptional regulator | 9 | -0.163 | 0 | [1, 3, 6, 7] |
| Rv1627c | *-* | Possible nonspecific lipid-transfer protein | 1 | 0.005 | 0 | [1] |
| Rv1628c | *-* | Conserved hypothetical protein | 10 | -0.436 | 0 |  |
| Rv1629 | *polA* | Possible DNA polymerase I | 2 | -0.222 | 0 | [1, 6] |
| Rv1630 | *rpsA* | Possible ribosomal protein | 2 | -0.351 | 0 | [1, 6, 9] |
| Rv1636 | *TB15.3* | Iron-regulated Conserved hypothetical protein | 10 | 0.005 | 0 | [1, 3, 7] |
| Rv1637c | *-* | Conserved hypothetical protein | 10 | -0.102 | 0 | [1] |
| Rv1638 | *uvrA* | Possible excinuclease abc | 2 | -0.249 | 0 | [1, 6] |
| Rv1641 | *infC* | Possible initiation factor | 2 | -0.764 | 0 | [1, 6, 9] |
| Rv1643 | *rplT* | Possible 50s ribosomal protein | 2 | -0.611 | 0 | [1, 9, 10] |
| Rv1652 | *argC* | Possible n-acetl-gamma-glutamyl-phoshate reductase | 7 | 0.226 | 0 | [7] |
| Rv1653 | *argJ* | Possible glutamate n-acetyltransferase | 7 | 0.199 | 0 |  |
| Rv1654 | *argB* | Possible acetylglutamate kinase | 7 | 0.32 | 0 | [1, 3, 7] |
| Rv1655 | *argD* | Possible acetylornithine aminotransferase | 7 | 0.294 | 0 | [7] |
| Rv1656 | *argF* | Possible ornithine carbamoyltransferase | 7 | -0.093 | 0 |  |
| Rv1659 | *argH* | Possible argininosuccinate lyase | 7 | 0.022 | 0 | [3] |
| Rv1665 | *pks11* | Possible chalcone synthase | 1 | 0.163 | 0 |  |
| Rv1676 | *-* | Hypothetical protein | 10 | -0.317 | 0 | [1] |
| Rv1679 | *fadE16* | Possible acyl-CoA dehydrogenase | 1 | 0.321 | 0 | [1, 3, 7] |
| Rv1683 | *-* | Possible long-chain acyl-CoA synthase | 1 | 0.027 | 0 | [1, 6] |
| Rv1699 | *pyrG* | Possible ctp synthase | 7 | -0.201 | 0 | [1] |
| Rv1703c | *-* | Possible catechol-o-methyltransferase | 7 | -0.115 | 0 | [1, 3, 6, 7] |
| Rv1707 | *-* | Possible conserved transmembrane protein | 3 | 0.691 | 11 |  |
| Rv1719 | *-* | Possible transcriptional regulatory protein | 9 | -0.075 | 0 |  |
| Rv1721c | *-* | Conserved hypothetical protein | 0 | -0.104 | 0 |  |
| Rv1722 | *-* | Possible carboxylase | 1 | -0.274 | 0 |  |
| Rv1731 | *gabD2* | Possible succinate-semialdehyde dehydrogenase [NADP+] dependent | 7 | 0 | 0 | [1, 6, 7, 10] |
| Rv1732c | *-* | Conserved hypothetical protein | 10 | -0.109 | 0 | [7] |
| Rv1737c | *narK2* | Possible nitrate/nitrite transporter | 3 | 0.825 | 12 | [1] |
| Rv1738 | *-* | Conserved hypothetical protein | 10 | -0.439 | 0 | [1, 3, 6, 10] |
| Rv1742 | *-* | Hypothetical protein | 8 | -0.302 | 0 | [6] |
| Rv1746 | *pknF* | Anchored-membrane serine/threonine-protein kinase | 9 | -0.188 | 1 |  |
| Rv1747 | *-* | Possible conserved transmembrane ATP-binding protein abc transporter | 3 | 0.058 | 6 | [1, 6] |
| Rv1748 | *-* | Hypothetical protein | 10 | -0.344 | 1 | [5, 9] |
| Rv1749c | *-* | Possible integral membrane protein | 3 | 0.325 | 4 | [5] |
| Rv1751 | *-* | Possible oxidoreductase | 7 | -0.127 | 0 | [1, 6] |
| Rv1754c | *-* | Conserved hypothetical protein | 10 | -0.449 | 1 | [6] |
| Rv1761c | *-* | Hypothetical exported protein | 3 | 0.302 | 1 |  |
| Rv1762c | *-* | Hypothetical protein | 10 | -0.155 | 0 | [1, 6] |
| Rv1766 | *-* | Conserved hypothetical protein | 10 | 0.463 | 0 |  |
| Rv1767 | *-* | Conserved hypothetical protein | 10 | 0.102 | 0 |  |
| Rv1769 | *-* | Conserved hypothetical protein | 10 | -0.202 | 0 | [1] |
| Rv1770 | *-* | Conserved hypothetical protein | 10 | -0.025 | 0 | [1, 10] |
| Rv1771 | *-* | L-gulono-1,4-lactone dehydrogenase | 7 | -0.373 | 0 | [1, 3, 6, 9, 10] |
| Rv1774 | *-* | Possible oxidoreductase | 7 | -0.044 | 0 |  |
| Rv1778c | *-* | Hypothetical protein | 10 | -0.019 | 0 | [1] |
| Rv1779c | *-* | Hypothetical integral membrane protein | 3 | -0.236 | 4 | [6] |
| Rv1780 | *-* | Conserved hypothetical protein | 10 | 0.067 | 0 | [1, 5] |
| Rv1782 | *-* | Possible conserved membrane protein | 3 | -0.048 | 1 | [1] |
| Rv1784 | *-* | Conserved hypothetical protein | 10 | -0.123 | 0 | [2, 3, 6] |
| Rv1789 | *PPE26* | PPE family protein | 6 | 0.344 | 0 | [3] |
| Rv1793 | *esxN* | Putative esat-6 like protein | 3 | -0.111 | 0 | [1-3] |
| Rv1794 | *-* | Conserved hypothetical protein | 10 | -0.121 | 0 | [1, 3, 7] |
| Rv1795 | *-* | Conserved hypothetical membrane protein | 3 | 0.715 | 10 | [1] |
| Rv1796 | *mycP5* | Possible proline rich membrane-anchored mycosin | 7 | -0.03 | 2 | [3] |
| Rv1806 | *PE20* | PE family protein | 6 | 0.809 | 0 |  |
| Rv1808 | *PPE32* | PPE family protein | 6 | 0.504 | 0 |  |
| Rv1809 | *PPE33* | PPE family protein | 6 | 0.169 | 0 |  |
| Rv1816 | *-* | Possible transcriptional regulatory protein | 9 | 0.075 | 0 |  |
| Rv1817 | *-* | Possible flavoprotein | 7 | 0.051 | 0 | [6] |
| Rv1819c | *-* | Possible drugs-transport transmembrane ATP-binding protein abc transporter | 3 | 0.14 | 6 | [1, 6, 10] |
| Rv1822 | *pgsA2* | Possiblecdp-diacylglycerol--glycerol-3-phosphate 3-phosphatidyltransferase | 1 | 0.72 | 4 | [3] |
| Rv1823 | *-* | Conserved hypothetical protein | 10 | 0.122 | 0 | [6] |
| Rv1825 | *-* | Conserved hypothetical protein | 10 | -0.044 | 1 | [10] |
| Rv1827 | *garA* | Conserved hypothetical protein with fha domain | 10 | -0.368 | 0 | [1, 2, 7] |
| Rv1832 | *gcvB* | Possible glycine dehydrogenase | 7 | 0.077 | 0 | [1, 6] |
| Rv1833c | *-* | Possible haloalkane dehalogenase | 7 | -0.1 | 0 | [1, 2] |
| Rv1836c | *-* | Conserved hypothetical protein | 10 | -0.087 | 1 | [1, 3, 5, 10] |
| Rv1837c | *glcB* | Possible malate synthase g | 7 | -0.155 | 0 | [1-3, 5-7] |
| Rv1843c | *guaB1* | Possible inosine-5'-monophosphate dehydrogenase | 7 | 0.083 | 0 | [1, 6, 7] |
| Rv1847 | *-* | Conserved hypothetical protein | 10 | -0.137 | 0 |  |
| Rv1848 | *ureA* | Urease gamma subunit | 7 | -0.227 | 0 |  |
| Rv1850 | *ureC* | Urease alpha subunit | 7 | 0.008 | 0 |  |
| Rv1852 | *ureG* | Urease accessory protein | 7 | 0.048 | 0 |  |
| Rv1855c | *-* | Possible oxidoreductase | 7 | 0.126 | 0 | [1] |
| Rv1856c | *-* | Possible oxidoreductase | 7 | 0.102 | 0 | [1, 3] |
| Rv1857 | *modA* | Possible molybdate-binding lipoprotein | 3 | 0.339 | 0 |  |
| Rv1858 | *modB* | Possible molbdenum-transport integral membrane protein abc transporter | 3 | 0.935 | 6 |  |
| Rv1860 | *apa* | Alanine and proline rich secreted protein | 3 | -0.256 | 1 | [2, 7] |
| Rv1862 | *adhA* | Possible alcohol dehydrogenase | 7 | 0.023 | 0 |  |
| Rv1864c | *-* | Conserved hypothetical protein | 10 | -0.207 | 0 |  |
| Rv1869c | *-* | Possible reductase | 7 | -0.002 | 0 | [1-3] |
| Rv1871c | *-* | Conserved hypothetical protein | 10 | -0.488 | 0 | [1] |
| Rv1872c | *lldD2* | Possible l-lactate dehydrogenase | 7 | 0.058 | 0 | [1, 3, 6, 9, 10] |
| Rv1874 | *-* | Hypothetical protein | 10 | -0.042 | 0 | [1] |
| Rv1875 | *-* | Conserved hypothetical protein | 10 | -0.149 | 0 | [6, 7] |
| Rv1876 | *bfrA* | Possible bacterioferritin | 7 | -0.455 | 0 | [1, 2, 9, 10] |
| Rv1878 | *glnA3* | Possible glutamine synthetase | 7 | 0.216 | 0 | [1] |
| Rv1881c | *lppE* | Possible conserved lipoprotein | 3 | -0.105 | 1 | [6] |
| Rv1884c | *rpfC* | Possible resuscitation-promoting factor | 3 | -0.028 | 0 | [2] |
| Rv1885c | *-* | Conserved hypothetical protein | 10 | -0.262 | 1 | [3] |
| Rv1886c | *fbpB* | Secreted antigen | 1 | -0.179 | 1 | [1, 2, 7] |
| Rv1891 | *-* | Conserved hypothetical protein | 10 | -0.144 | 0 | [2] |
| Rv1899c | *lppD* | Possible lipoprotein | 3 | -0.09 | 0 | [1, 2, 7] |
| Rv1905c | *aao* | Possible d-amino acid oxidase | 7 | 0.034 | 0 |  |
| Rv1908c | *katG* | Catalase-peroxidase-peroxynitritase | 0 | -0.325 | 0 | [1-3, 6, 7, 10] |
| Rv1909c | *furA* | Ferric uptake regulation protein | 9 | -0.015 | 0 | [7] |
| Rv1914c | *-* | Hypothetical protein | 10 | 0.18 | 0 | [1] |
| Rv1919c | *-* | Conserved hypothetical protein | 10 | -0.281 | 0 | [1, 3, 10] |
| Rv1923 | *lipD* | Possible lipase | 7 | 0.04 | 0 | [7] |
| Rv1924c | *-* | Hypothetical protein | 10 | 0.802 | 3 | [10] |
| Rv1925 | *fadD31* | Possible acyl-CoA ligase | 1 | -0.013 | 0 | [1, 3, 6, 7, 10] |
| Rv1926c | *mpt63* | Immunogenic protein | 3 | 0.328 | 1 | [2, 7] |
| Rv1928c | *-* | Possible short-chain type dehydrogenase/reductase | 7 | 0.225 | 0 |  |
| Rv1932 | *tpx* | Possible thiol peroxidase | 0 | 0.286 | 0 | [1-3, 7] |
| Rv1942c | *-* | Conserved hypothetical protein | 0 | 0.287 | 0 |  |
| Rv1943c | *-* | Conserved hypothetical protein | 0 | -0.314 | 0 |  |
| Rv1944c | *-* | Conserved hypothetical protein | 10 | -0.289 | 0 |  |
| Rv1961 | *-* | Hypothetical protein | 10 | -0.341 | 0 |  |
| Rv1962c | *-* | Conserved hypothetical protein with pin domain | 0 | 0.253 | 0 |  |
| Rv1977 | *-* | Conserved hypothetical protein | 10 | -0.191 | 1 | [1, 6] |
| Rv1978 | *-* | Conserved hypothetical protein | 10 | -0.055 | 0 | [1, 6, 10] |
| Rv1980c | *mpt64* | Immunogenic protein | 3 | -0.137 | 1 | [2, 7] |
| Rv1982A | *-* | Conserved hypothetical protein | 0 | -0.17 | 0 |  |
| Rv1984c | *cfp21* | Possible cutinase precursor | 3 | 0.217 | 0 | [2, 3, 7] |
| Rv1987 | *-* | Possible chitinase | 3 | 0.215 | 1 |  |
| Rv1990c | *-* | Possible transcriptional regulatory protein | 9 | 0.038 | 0 |  |
| Rv1993c | *-* | Conserved hypothetical protein | 10 | 0.147 | 0 | [1, 3, 5] |
| Rv1995 | *-* | Hypothetical protein | 10 | -0.341 | 0 | [1] |
| Rv1998c | *-* | Conserved hypothetical protein | 10 | -0.023 | 0 |  |
| Rv2010 | *-* | Conserved hypothetical protein | 0 | 0.061 | 0 |  |
| Rv2018 | *-* | Conserved hypothetical protein | 10 | -0.09 | 0 |  |
| Rv2019 | *-* | Hypothetical protein | 10 | -0.063 | 0 |  |
| Rv2022c | *-* | Conserved hypothetical protein | 10 | -0.398 | 0 |  |
| Rv2030c | *-* | Conserved hypothetical protein | 10 | -0.216 | 0 | [1, 3, 6, 10] |
| Rv2031c | *hspX* | Heat shock protein | 0 | -0.52 | 0 | [1, 3, 5-7, 9, 10] |
| Rv2034 | *-* | Possible arsr-type repressor protein | 9 | -0.384 | 0 |  |
| Rv2037c | *-* | Possible conserved transmembrane protein | 3 | 0.154 | 0 | [1, 6] |
| Rv2041c | *-* | Possible sugar-binding lipoprotein | 3 | -0.131 | 0 | [3, 6] |
| Rv2042c | *-* | Conserved hypothetical protein | 10 | -0.104 | 0 | [1] |
| Rv2046 | *lppI* | Possible lipoprotein | 3 | 0.083 | 0 | [1, 3] |
| Rv2050 | *-* | Conserved hypothetical protein | 10 | -0.907 | 0 |  |
| Rv2051c | *ppm1* | Polyprenol-monophosphomannose synthase | 3 | 0.058 | 7 | [1, 6] |
| Rv2052c | *-* | Conserved hypothetical protein | 10 | 0.021 | 0 | [6] |
| Rv2054 | *-* | Conserved hypothetical protein | 10 | 0.01 | 0 | [1, 6, 9, 10] |
| Rv2061c | *-* | Conserved hypothetical protein | 10 | -0.266 | 0 | [1, 3, 6, 10] |
| Rv2063 | *-* | Conserved hypothetical protein | 0 | -0.209 | 0 |  |
| Rv2068c | *blaC* | Beta-lactamase | 7 | -0.091 | 0 | [1-3, 6, 10] |
| Rv2074 | *-* | Conserved hypothetical protein | 10 | -0.178 | 0 | [2] |
| Rv2080 | *lppJ* | Possible lipoprotein | 3 | -0.343 | 1 | [2, 5] |
| Rv2088 | *pknJ* | Possible transmembrane serine/threonine-protein | 9 | 0.075 | 0 |  |
| Rv2091c | *-* | Possible membrane protein | 3 | -0.685 | 1 | [1, 3, 5, 9, 10] |
| Rv2092c | *helY* | Possible ATP-dependent DNA helicase | 2 | -0.263 | 0 | [1, 6] |
| Rv2093c | *tatC* | Possible sec-independent protein translocase transmembrane protein | 3 | 0.552 | 6 | [6] |
| Rv2094c | *tatA* | Possible sec-independent protein translocase membrane-bound protein | 3 | -0.235 | 1 | [3] |
| Rv2095c | *-* | Conserved hypothetical protein | 10 | 0.084 | 0 |  |
| Rv2109c | *prcA* | Proteasome alpha subunit | 7 | -0.283 | 0 | [1, 6, 7] |
| Rv2110c | *prcB* | Proteasome beta subunit | 7 | 0.021 | 0 | [1, 2, 7, 9] |
| Rv2112c | *-* | Conserved hypothetical protein | 10 | -0.167 | 0 | [1] |
| Rv2113 | *-* | Possible integral membrane protein | 3 | 0.732 | 8 | [1, 10] |
| Rv2114 | *-* | Hypothetical protein | 10 | -0.308 | 0 | [6] |
| Rv2116 | *lppK* | Possible conserved lipoprotein | 3 | -0.094 | 0 | [1, 3, 10] |
| Rv2118c | *-* | Possible RNA methyltransferase | 7 | -0.051 | 0 | [1] |
| Rv2120c | *-* | Possible conserved integral membrane protein | 3 | 1.021 | 5 | [1, 3] |
| Rv2121c | *hisG* | Possible ATP phosphoribosyltransferase | 7 | 0.047 | 0 | [1] |
| Rv2127 | *ansP1* | Possible l-asparagine permease | 3 | 0.7 | 12 | [3] |
| Rv2129c | *-* | Possible oxidoreductase | 7 | 0.18 | 0 | [1, 3, 6, 9, 10] |
| Rv2130c | *mshC* | Cysteine:1d-myo-inosityl 2-amino-2-deoxy--d-glucopyranoside ligase | 7 | -0.211 | 0 | [1, 3] |
| Rv2131c | *cysQ* | Possible monophosphatase | 7 | -0.067 | 0 | [1, 3] |
| Rv2134c | *-* | Conserved hypothetical protein | 10 | -0.098 | 0 |  |
| Rv2135c | *-* | Conserved hypothetical protein | 10 | -0.076 | 0 |  |
| Rv2136c | *-* | Possible conserved transmembrane protein | 3 | 0.862 | 5 |  |
| Rv2138 | *lppL* | Possible conserved lipoprotein | 3 | 0.078 | 1 | [3] |
| Rv2139 | *pyrD* | Possible dihydroorotate dehydrogenase | 7 | -0.005 | 0 | [6, 10] |
| Rv2140c | *TB18.6* | Conserved hypothetical protein | 10 | -0.104 | 0 | [1-3, 7] |
| Rv2141c | *dapE2* | Conserved hypothetical protein | 7 | -0.053 | 0 |  |
| Rv2142c | *-* | Hypothetical protein | 0 | -0.415 | 0 |  |
| Rv2145c | *wag31* | Conserved hypothetical protein | 3 | -0.726 | 0 | [1, 3, 6, 7] |
| Rv2146c | *-* | Possible conserved transmembrane protein | 3 | 1.377 | 2 |  |
| Rv2148c | *-* | Conserved hypothetical protein | 10 | -0.157 | 0 |  |
| Rv2149c | *yfiH* | Conserved hypothetical protein | 10 | 0.091 | 0 | [6] |
| Rv2159c | *-* | Conserved hypothetical protein | 10 | -0.05 | 0 | [1, 6, 10] |
| Rv2161c | *-* | Conserved hypothetical protein | 7 | 0.135 | 0 | [1, 7] |
| Rv2165c | *-* | Conserved hypothetical protein | 10 | -0.105 | 0 | [6] |
| Rv2166c | *-* | Conserved hypothetical protein | 10 | -0.489 | 0 |  |
| Rv2169c | *-* | Possible conserved transmembrane protein | 3 | -0.122 | 2 |  |
| Rv2171 | *lppM* | Possible conserved lipoprotein | 3 | 0.062 | 1 | [3] |
| Rv2172c | *-* | Conserved hypothetical protein | 10 | -0.059 | 0 | [1] |
| Rv2174 | *mptA* | Alpha(1->6)mannosyltransferase | 3 | 0.795 | 12 |  |
| Rv2175c | *-* | Conserved hypothetical regulatory protein | 9 | -0.141 | 0 | [1] |
| Rv2181 | *-* | Possible alpha(1->2)mannosyltransferase | 3 | 0.68 | 10 | [3, 6] |
| Rv2183c | *-* | Conserved hypothetical protein | 10 | -0.069 | 0 | [3] |
| Rv2185c | *TB16.3* | Conserved hypothetical protein | 10 | -0.394 | 0 | [1, 3, 7] |
| Rv2187 | *fadD15* | Possible long-chain-fatty-acid-CoA ligase | 1 | 0.058 | 0 | [1, 7] |
| Rv2192c | *trpD* | Possible anthranilate phosphoribosyltransferase | 7 | 0.196 | 0 | [1, 2] |
| Rv2194 | *qcrC* | Possible ubiquinol-cytochrome c reductase | 7 | -0.045 | 2 | [3, 5, 10] |
| Rv2195 | *qcrA* | Possible rieske iron-sulfur protein | 7 | -0.164 | 3 | [1, 3, 5, 6, 9, 10] |
| Rv2196 | *qcrB* | Possible ubiquinol-cytochrome c reductase | 7 | 0.324 | 9 | [1, 3, 6, 10] |
| Rv2197c | *-* | Possible conserved transmembrane protein | 3 | 0.514 | 4 | [1, 3] |
| Rv2198c | *mmpS3* | Possible conserved membrane protein | 3 | -0.482 | 1 | [1, 3, 10] |
| Rv2200c | *ctaC* | Possible transmembrane cytochrome c oxidase | 7 | -0.094 | 3 | [1-3, 5, 10] |
| Rv2201 | *asnB* | Possible asparagine synthetase | 7 | -0.032 | 0 | [1, 2, 6, 10] |
| Rv2202c | *adoK* | Adenosine kinase | 7 | 0.144 | 0 | [1] |
| Rv2203 | *-* | Possible conserved membrane protein | 3 | -0.178 | 1 | [3] |
| Rv2204c | *-* | Conserved hypothetical protein | 10 | -0.204 | 0 | [1, 3] |
| Rv2207 | *cobT* | Possiblenicotinate-nucleotide-dimethylbenzimidazol phosphoribosyltransferase | 7 | 0.269 | 0 |  |
| Rv2210c | *ilvE* | Possible branched-chain amino acid transaminase | 7 | 0.001 | 0 | [1] |
| Rv2211c | *gcvT* | Possible aminomethyltransferase | 7 | 0.137 | 0 |  |
| Rv2213 | *pepB* | Possible aminopeptidase | 7 | 0.073 | 0 | [1] |
| Rv2215 | *dlaT* | Dihydrolipoamide acyltransferase | 7 | -0.127 | 0 | [1, 6, 7] |
| Rv2216 | *-* | Conserved hypothetical protein | 10 | 0.049 | 0 | [1, 9] |
| Rv2219 | *-* | Possible conserved transmembrane protein | 3 | -0.039 | 2 | [3, 6, 10] |
| Rv2219A | *-* | Possible conserved membrane protein | 3 | 0.756 | 2 |  |
| Rv2220 | *glnA1* | Glutamine synthetase | 7 | -0.421 | 0 | [1-3, 5-7, 9, 10] |
| Rv2222c | *glnA2* | Possible glutamine synthetase | 7 | -0.255 | 0 | [1, 3, 6, 10] |
| Rv2224c | *-* | Possible exported protease | 3 | -0.214 | 1 | [1-3, 5, 6, 9, 10] |
| Rv2226 | *-* | Conserved hypothetical protein | 10 | -0.458 | 0 | [1, 6] |
| Rv2228c | *-* | Conserved hypothetical protein | 10 | -0.164 | 0 | [1] |
| Rv2229c | *-* | Conserved hypothetical protein | 10 | -0.545 | 0 | [6] |
| Rv2230c | *-* | Conserved hypothetical protein | 10 | 0.162 | 0 |  |
| Rv2235 | *-* | Possible conserved transmembrane protein | 3 | -0.072 | 3 | [6] |
| Rv2237 | *-* | Conserved hypothetical protein | 10 | -0.069 | 0 | [1] |
| Rv2240c | *-* | Hypothetical protein | 10 | -0.069 | 0 |  |
| Rv2241 | *aceE* | Possible pyruvate dehydrogenase e1 component | 7 | -0.382 | 0 | [1, 2, 6, 7, 10] |
| Rv2244 | *acpM* | Meromycolate extension acyl carrier protein | 1 | -0.249 | 0 | [1, 3, 6, 7, 10] |
| Rv2245 | *kasA* | 3-oxoacyl-[acyl-carrier protein] synthase | 1 | 0.03 | 0 | [1, 6] |
| Rv2246 | *kasB* | 3-oxoacyl-[acyl-carrier protein] synthase | 1 | -0.002 | 0 | [1, 3, 6, 7, 10] |
| Rv2249c | *glpD1* | Possible glycerol-3-phosphate dehydrogenase | 7 | 0.065 | 0 | [6] |
| Rv2251 | *-* | Possible flavoprotein | 7 | 0.007 | 0 | [1, 2, 6] |
| Rv2257c | *-* | Conserved hypothetical protein | 10 | 0.136 | 0 |  |
| Rv2258c | *-* | Possible transcriptional regulatory protein | 9 | 0.175 | 0 | [1, 3] |
| Rv2280 | *-* | Possible dehydrogenase | 7 | 0.094 | 0 | [1] |
| Rv2284 | *lipM* | Possible esterase | 7 | -0.016 | 3 | [1, 3, 6, 10] |
| Rv2285 | *-* | Conserved hypothetical protein | 10 | 0.136 | 0 |  |
| Rv2289 | *cdh* | Possible cdp-diacylglycerol pyrophosphatase | 1 | -0.16 | 1 | [1, 3, 10] |
| Rv2294 | *-* | Possible aminotransferase | 7 | -0.083 | 0 | [1, 3] |
| Rv2296 | *-* | Possible haloalkane dehalogenase | 7 | -0.228 | 0 | [1, 3, 6, 7, 9, 10] |
| Rv2297 | *-* | Hypothetical protein | 10 | -0.297 | 0 | [6] |
| Rv2298 | *-* | Conserved hypothetical protein | 10 | -0.027 | 0 | [1-3, 6] |
| Rv2299c | *htpG* | Possible chaperone protein | 0 | -0.441 | 0 | [1, 3, 6, 7] |
| Rv2300c | *-* | Conserved hypothetical protein | 10 | -0.309 | 0 | [6] |
| Rv2301 | *cut2* | Possible cutinase | 3 | 0.135 | 1 | [2, 3, 7] |
| Rv2302 | *-* | Conserved hypothetical protein | 10 | -0.385 | 0 | [3] |
| Rv2305 | *-* | Hypothetical protein | 10 | 0.118 | 0 | [1, 3, 10] |
| Rv2314c | *-* | Conserved hypothetical protein | 10 | -0.051 | 0 | [1, 2] |
| Rv2315c | *-* | Conserved hypothetical protein | 10 | -0.142 | 0 |  |
| Rv2319c | *-* | Hypothetical protein | 10 | 0.084 | 0 |  |
| Rv2320c | *rocE* | Possible cationic amino acid transport integral membrane protein | 3 | 0.828 | 12 |  |
| Rv2323c | *-* | Conserved hypothetical protein | 10 | 0.096 | 0 |  |
| Rv2324 | *-* | Possible transcriptional regulatory protein | 9 | -0.095 | 0 |  |
| Rv2326c | *-* | Possible transmembrane ATP-binding protein abc transorter | 3 | 0.307 | 4 | [1, 10] |
| Rv2327 | *-* | Conserved hypothetical protein | 10 | -0.091 | 0 |  |
| Rv2330c | *lppP* | Possible lipoprotein | 3 | -0.06 | 1 |  |
| Rv2334 | *cysK1* | Possible cysteine synthase | 7 | 0.129 | 0 | [1, 2] |
| Rv2345 | *-* | Possible conserved transmembrane protein | 3 | -0.13 | 3 | [1, 5, 6, 10] |
| Rv2346c | *esxO* | Putative esat-6 like protein | 3 | -0.085 | 0 | [1, 2, 6] |
| Rv2349c | *plcC* | Possible phospholipase | 7 | -0.125 | 0 |  |
| Rv2350c | *plcB* | Possible membrane-associated phospholipase | 7 | -0.105 | 0 |  |
| Rv2351c | *plcA* | Possible membrane-associated phospholipase | 7 | -0.084 | 0 |  |
| Rv2357c | *glyS* | Possible glycyl-tRNA synthetase | 2 | -0.508 | 0 | [1, 3, 6, 7] |
| Rv2358 | *-* | Possible transcriptional regulatory protein | 9 | 0.02 | 0 | [3] |
| Rv2359 | *furB* | Possible ferric uptake regulation protein | 9 | -0.443 | 0 | [1, 3] |
| Rv2361c | *uppS* | Long (c50) chain z-isoprenyl diphosphate synthase | 3 | -0.561 | 0 | [6, 10] |
| Rv2363 | *amiA2* | Possible amidase | 7 | 0.004 | 0 | [1] |
| Rv2376c | *cfp2* | Low molecular weight antigen | 3 | 0.306 | 1 | [2, 7] |
| Rv2387 | *-* | Conserved hypothetical protein | 10 | 0.563 | 10 | [3] |
| Rv2391 | *sirA* | Ferredoxin-dependent sulfite reductase | 7 | -0.391 | 0 | [7] |
| Rv2394 | *ggtB* | Possible gamma-glutamyltranspeptidase precursor | 7 | -0.111 | 0 | [1, 3, 10] |
| Rv2400c | *subI* | Possible sulfate-binding lipoprotein | 3 | -0.011 | 0 | [5, 10] |
| Rv2402 | *-* | Conserved hypothetical protein | 10 | -0.382 | 0 | [1, 6] |
| Rv2405 | *-* | Conserved hypothetical protein | 10 | -0.365 | 0 | [1, 6] |
| Rv2406c | *-* | Conserved hypothetical protein | 10 | 0.208 | 0 | [1] |
| Rv2410c | *-* | Conserved hypothetical protein | 10 | -0.167 | 0 | [1, 3, 10] |
| Rv2412 | *rpsT* | Possible 30s ribosomal protein | 2 | -0.859 | 0 | [6] |
| Rv2416c | *eis* | Enhanced intracellular survival protein | 0 | -0.095 | 0 | [1, 7] |
| Rv2427c | *proA* | Possible gamma-glutamyl phosphate reductase protein | 7 | 0.066 | 0 | [1, 6, 8] |
| Rv2429 | *ahpD* | Alkyl hydroperoxide reductase d protein | 0 | 0.173 | 0 | [1, 3, 7] |
| Rv2431c | *PE25* | PE family protein | 6 | 0.025 | 0 | [2] |
| Rv2441c | *rpmA* | Possible 50s ribosomal protein | 2 | -0.486 | 0 | [6] |
| Rv2442c | *rplU* | Possible 50s ribosomal protein | 2 | -0.168 | 0 | [1] |
| Rv2443 | *dctA* | Possible c4-dicarboxylate-transport transmembrane protein | 3 | 0.689 | 7 |  |
| Rv2445c | *ndkA* | Possible nucleoside diphosphate kinase | 7 | 0.106 | 0 | [1-3, 7] |
| Rv2448c | *valS* | Possible valyl-tRNA synthase protein | 2 | -0.304 | 0 | [6] |
| Rv2454c | *-* | Possible oxidoreductase | 7 | -0.123 | 0 | [1, 6] |
| Rv2455c | *-* | Possible oxidoreductase | 7 | -0.09 | 0 | [1, 3, 10] |
| Rv2457c | *clpX* | Possible ATP-dependent clp protease ATP-binding subunit | 7 | -0.163 | 0 | [3, 6] |
| Rv2460c | *clpP2* | Possible ATP-dependent clp protease proteolytic subunit 2 | 7 | -0.149 | 0 | [1, 3, 6, 10] |
| Rv2461c | *clpP1* | Possible ATP-dependent clp protease proteolytic subunit 1 | 7 | 0.039 | 0 | [1, 6] |
| Rv2462c | *tig* | Possible trigger factor (tf) protein | 3 | -0.387 | 0 | [1, 6, 7] |
| Rv2465c | *rpiB* | Ribose-5-phosphate isomerase | 7 | -0.193 | 0 | [2, 7] |
| Rv2466c | *-* | Conserved hypothetical protein | 10 | -0.29 | 0 |  |
| Rv2467 | *pepN* | Possible aminopeptidase | 7 | -0.156 | 0 | [1] |
| Rv2468c | *-* | Conserved hypothetical protein | 10 | 0.194 | 0 | [1] |
| Rv2470 | *glbO* | Possible globin | 7 | -0.53 | 0 |  |
| Rv2473 | *-* | Possible alanine and proline rich membrane protein | 3 | -0.189 | 1 |  |
| Rv2475c | *-* | Conserved hypothetical protein | 10 | -0.129 | 0 |  |
| Rv2476c | *gdh* | Possible NAD-dependent glutamate dehydrogenase | 7 | -0.056 | 0 | [1, 6, 10] |
| Rv2496c | *pdhB* | Possible pyruvate dehydrogenase e1 component | 7 | -0.064 | 0 | [1, 6] |
| Rv2497c | *pdhA* | Possible pyruvate dehydrogenase e1 component | 7 | -0.305 | 0 |  |
| Rv2498c | *citE* | Possible citrate (pro-3s)-lyase | 7 | 0.01 | 0 |  |
| Rv2504c | *sCoA* | Possible succinyl-CoA:3-ketoacid-coenzyme a transferase | 1 | -0.012 | 0 |  |
| Rv2506 | *-* | Possible transcriptional regulatory protein | 9 | -0.022 | 0 |  |
| Rv2507 | *-* | Possible conserved proline rich membrane protein | 3 | -0.372 | 1 |  |
| Rv2508c | *-* | Possible conserved integral membrane leucine and alanine rich protein | 3 | 0.773 | 12 |  |
| Rv2509 | *-* | Possible short-chain type dehydrogenase/reductase | 7 | 0.123 | 0 | [1, 6] |
| Rv2510c | *-* | Conserved hypothetical protein | 10 | 0.001 | 0 |  |
| Rv2517c | *-* | Hypothetical protein | 10 | -0.961 | 0 |  |
| Rv2518c | *lppS* | Possible conserved lipoprotein | 3 | -0.124 | 1 | [3] |
| Rv2526 | *-* | Hypothetical protein | 0 | -0.183 | 0 |  |
| Rv2528c | *mrr* | Possible restriction system protein | 2 | -0.305 | 0 | [1] |
| Rv2531c | *ad* | Possible amino acid decarboxylase | 7 | -0.162 | 0 | [6] |
| Rv2534c | *efp* | Possible elongation factor | 2 | -0.209 | 0 | [3, 7] |
| Rv2535c | *pepQ* | Possible cytoplasmic peptidase | 7 | 0.055 | 0 | [1] |
| Rv2536 | *-* | Possible conserved transmembrane protein | 3 | 0.253 | 4 | [3, 5, 9, 10] |
| Rv2537c | *aroD* | 3-dehydroquinate dehydratase | 7 | 0.145 | 1 |  |
| Rv2554c | *-* | Conserved hypothetical protein | 10 | -0.254 | 0 | [1, 6] |
| Rv2557 | *-* | Conserved hypothetical protein | 10 | -0.198 | 0 | [1] |
| Rv2560 | *-* | Possible proline and glycine rich transmembrane protein | 3 | 0.488 | 4 | [3] |
| Rv2563 | *-* | Possible glutamine-transport transmembrane protein abc transporter | 3 | 0.714 | 4 | [1-3, 10] |
| Rv2564 | *glnQ* | Possible glutamine-transport ATP-binding protein abc transporter | 3 | 0.097 | 0 | [1] |
| Rv2565 | *-* | Conserved hypothetical protein | 10 | 0.032 | 0 | [6] |
| Rv2572c | *aspS* | Possible aspartyl-tRNA synthetase | 2 | -0.157 | 0 | [1, 3, 6] |
| Rv2574 | *-* | Conserved hypothetical protein | 10 | -0.105 | 0 | [1, 10] |
| Rv2575 | *-* | Possible conserved membrane glycine rich protein | 3 | -0.169 | 1 | [2] |
| Rv2577 | *-* | Conserved hypothetical protein | 10 | -0.412 | 0 | [6] |
| Rv2579 | *dhaA* | Possible haloalkane dehalogenase | 7 | -0.294 | 0 | [1] |
| Rv2581c | *-* | Posible glyoxalase II | 0 | -0.021 | 0 | [1, 3, 5, 7] |
| Rv2582 | *ppiB* | Possible peptidyl-prolyl cis-trans isomerase | 2 | -0.256 | 1 | [1, 3, 10] |
| Rv2584c | *apt* | Adenine phosphoribosyltransferase | 7 | 0.274 | 0 | [1] |
| Rv2586c | *secF* | Possible protein-export membrane protein | 3 | 0.098 | 6 | [1, 3, 6, 10] |
| Rv2587c | *secD* | Possible protein-export membrane protein | 3 | 0.094 | 6 | [1, 3, 6, 10] |
| Rv2588c | *yajC* | Possible conserved membrane protein secretion factor | 3 | -0.151 | 1 | [1, 3, 5] |
| Rv2589 | *gabT* | 4-aminobutyrate aminotransferase | 7 | 0.211 | 0 | [1] |
| Rv2597 | *-* | Possible membrane protein | 3 | 0.031 | 1 | [1, 3, 9, 10] |
| Rv2599 | *-* | Possible conserved membrane protein | 3 | -0.194 | 1 |  |
| Rv2602 | *-* | Conserved hypothetical protein with pin domain | 0 | 0.157 | 0 |  |
| Rv2603c | *-* | Highly Conserved hypothetical protein | 10 | -0.324 | 0 | [1] |
| Rv2605c | *tesB2* | Possible acyl-CoA thioesterase II | 1 | -0.298 | 0 | [1, 6, 7] |
| Rv2606c | *snzP* | Possible pyridoxine biosynthesis protein | 7 | 0.148 | 0 | [1, 3, 5, 6] |
| Rv2607 | *pdxH* | Possible pyridoxamine 5'-phosphate oxidase | 7 | -0.533 | 0 |  |
| Rv2612c | *pgsA1* | Possible pi synthase | 1 | 0.634 | 3 | [3] |
| Rv2614c | *thrS* | Possible threonyl-tRNA synthetase | 2 | -0.372 | 0 | [1] |
| Rv2617c | *-* | Possible transmembrane protein | 3 | 0.725 | 3 |  |
| Rv2619c | *-* | Conserved hypothetical protein | 10 | -0.157 | 0 | [7] |
| Rv2620c | *-* | Possible conserved transmembrane protein | 3 | 1.169 | 4 |  |
| Rv2623 | *TB31.7* | Conserved hypothetical protein | 10 | 0.104 | 0 | [1, 3, 6, 7, 10] |
| Rv2626c | *-* | Conserved hypothetical protein | 10 | 0.042 | 0 | [1, 3, 6, 7] |
| Rv2627c | *-* | Conserved hypothetical protein | 10 | -0.245 | 0 | [1, 10] |
| Rv2632c | *-* | Conserved hypothetical protein | 10 | -0.269 | 0 | [1] |
| Rv2637 | *dedA* | Possible transmembrane protein | 3 | 0.702 | 4 | [3] |
| Rv2658c | *-* | Possible prophage protein | 5 | -0.179 | 0 |  |
| Rv2667 | *clpC2* | Possible ATP-dependent protease ATP-binding subunit | 7 | -0.122 | 0 | [6] |
| Rv2671 | *ribD* | Possible bifunctional enzyme riboflavin biosynthesis protein | 7 | 0.022 | 0 | [6] |
| Rv2672 | *-* | Possible secreted protease | 7 | 0.043 | 1 | [2, 3, 10] |
| Rv2673 | *-* | Possible conserved integral membrane protein | 3 | 0.588 | 8 | [3] |
| Rv2674 | *-* | Conserved hypothetical protein | 10 | -0.574 | 0 |  |
| Rv2676c | *-* | Conserved hypothetical protein | 10 | -0.232 | 0 | [1] |
| Rv2678c | *hemE* | Possible uroporphyrinogen decarboxylase | 7 | 0.318 | 0 | [3, 6] |
| Rv2680 | *-* | Conserved hypothetical protein | 10 | -0.243 | 0 |  |
| Rv2683 | *-* | Conserved hypothetical protein | 10 | 0.128 | 0 |  |
| Rv2689c | *-* | Conserved hypothetical alanine and valine and glycine rich protein | 10 | -0.044 | 0 |  |
| Rv2691 | *ceoB* | Potassium uptake protein | 3 | 0.02 | 0 | [1, 7] |
| Rv2692 | *ceoC* | Potassium uptake protein | 3 | 0.105 | 0 | [1] |
| Rv2693c | *-* | Possible conserved integral membrane alanine and leucine rich protein | 3 | 0.711 | 6 | [2] |
| Rv2694c | *-* | Conserved hypothetical protein | 10 | -0.616 | 0 |  |
| Rv2696c | *-* | Conserved hypothetical alanine and glycine and valine rich protein | 10 | -0.227 | 0 | [6] |
| Rv2697c | *dut* | Possible deoxyuridine 5'-triphosphate nucleotidohydrolase | 7 | 0.212 | 0 |  |
| Rv2698 | *-* | Possible conserved alanine rich transmembrane protein | 3 | 0.406 | 2 |  |
| Rv2700 | *-* | Possible conserved secreted alanine rich protein | 3 | -0.063 | 1 | [10] |
| Rv2702 | *ppgK* | Polyphosphate glucokinase | 7 | -0.013 | 0 | [3] |
| Rv2703 | *sigA* | RNA polymerase sigma factor | 2 | -0.64 | 0 | [1, 6, 7, 10] |
| Rv2704 | *-* | Conserved hypothetical protein | 10 | 0.106 | 0 | [1] |
| Rv2710 | *sigB* | RNA polymerase sigma factor | 2 | -0.442 | 0 | [6] |
| Rv2711 | *ideR* | Iron-dependent repressor and activator | 9 | -0.167 | 0 | [1, 3] |
| Rv2713 | *sthA* | Possible soluble pyridine nucleotide transhydrogenase | 7 | -0.004 | 0 | [6] |
| Rv2714 | *-* | Conserved hypothetical alanine and leucine rich protein | 10 | -0.259 | 0 | [1] |
| Rv2716 | *-* | Conserved hypothetical protein | 10 | -0.142 | 0 | [7] |
| Rv2717c | *-* | Conserved hypothetical protein | 10 | -0.13 | 0 |  |
| Rv2720 | *lexA* | Repressor lexa | 9 | 0.026 | 0 |  |
| Rv2721c | *-* | Possible conserved transmembrane alanine and glycine rich protein | 3 | -0.097 | 2 | [1-3, 5, 10] |
| Rv2723 | *-* | Possible conserved integral membrane protein | 3 | 0.612 | 9 |  |
| Rv2728c | *-* | Conserved hypothetical alanine rich protein | 10 | 0.242 | 0 | [3] |
| Rv2731 | *-* | Conserved hypothetical alanine and arginine rich protein | 10 | -0.811 | 0 | [6, 7, 9] |
| Rv2732c | *-* | Possible conserved transmembrane protein | 3 | 0.364 | 4 |  |
| Rv2740 | *ephG* | Epoxide hydrolase | 0 | 0.05 | 0 | [1, 3, 6] |
| Rv2744c | *35kd_ag* | Conserved 35 kDa alanine rich protein | 10 | -0.464 | 0 | [1, 3, 5-7, 9, 10] |
| Rv2746c | *pgsA3* | Possible PGP synthase | 1 | 0.83 | 4 |  |
| Rv2753c | *dapA* | Possible dihydrodipicolinate synthase | 7 | 0.226 | 0 | [1] |
| Rv2754c | *thyX* | Possible thymidylate synthase | 7 | -0.187 | 0 |  |
| Rv2765 | *-* | Possible alanine rich hydrolase | 7 | -0.117 | 0 |  |
| Rv2766c | *fabG5* | Possible short-chain type dehydrogenase/reductase | 7 | 0.258 | 0 | [1-3] |
| Rv2772c | *-* | Possible conserved transmembrane protein | 3 | 0.193 | 2 |  |
| Rv2773c | *dapB* | Dihydrodipicolinate reductase | 7 | 0.178 | 0 | [1, 7] |
| Rv2778c | *-* | Conserved hypothetical protein | 10 | -0.39 | 0 |  |
| Rv2783c | *gpsI* | Bifunctional protein polyribonucleotide nucleotidyltransferase | 2 | 0.044 | 0 | [1, 6, 7, 9] |
| Rv2784c | *lppU* | Possible lipoprotein | 3 | -0.11 | 0 |  |
| Rv2785c | *rpsO* | Possible 30s ribosomal protein | 2 | -0.597 | 0 | [1, 6, 9, 10] |
| Rv2788 | *sirR* | Possible transcriptional repressor | 9 | -0.261 | 0 |  |
| Rv2789c | *fadE21* | Possible acyl-CoA dehydrogenase | 1 | -0.049 | 0 |  |
| Rv2790c | *ltp1* | Possible lipid-transfer protein | 3 | -0.279 | 0 | [6] |
| Rv2793c | *truB* | Possible tRNA pseudouridine synthase | 2 | -0.037 | 0 | [6] |
| Rv2801c | *-* | Conserved hypothetical protein | 0 | -0.086 | 0 |  |
| Rv2831 | *echA16* | Possible enoyl-CoA hydratase | 1 | 0.077 | 0 | [1, 2, 5-7, 10] |
| Rv2833c | *ugpB* | Possible sn-glycerol-3-phosphate-binding lipoprotein | 3 | -0.17 | 0 | [3, 6] |
| Rv2837c | *-* | Conserved hypothetical protein | 10 | 0.199 | 0 |  |
| Rv2841c | *nusA* | Possible n utilization substance protein a | 2 | -0.287 | 0 | [1] |
| Rv2842c | *-* | Conserved hypothetical protein | 10 | -0.06 | 0 | [1, 6, 10] |
| Rv2843 | *-* | Possible conserved transmembrane alanine rich protein | 3 | 0.28 | 2 |  |
| Rv2844 | *-* | Conserved hypothetical alanine rich protein | 10 | -0.023 | 0 | [3] |
| Rv2845c | *proS* | Possible prolyl-tRNA synthetase | 2 | -0.138 | 0 | [6, 10] |
| Rv2846c | *efpA* | Possible integral membrane efflux protein | 3 | 0.707 | 14 |  |
| Rv2852c | *mqo* | Possible malate:quinone oxidoreductase | 7 | -0.071 | 0 | [1, 10] |
| Rv2855 | *mtr* | NADPH-dependent mycothiol reductase | 7 | 0.029 | 0 | [1] |
| Rv2857c | *-* | Possible short-chain type dehydrogenase/reductase | 7 | 0.241 | 0 | [1] |
| Rv2860c | *glnA4* | Possible glutamine synthetase | 7 | -0.016 | 0 | [1] |
| Rv2868c | *gcpE* | Possible gcpe protein | 10 | 0.103 | 0 | [1] |
| Rv2869c | *rip* | Membrane bound metalloprotease | 3 | 0.484 | 4 | [9] |
| Rv2873 | *mpt83* | Cell surface lipoprotein | 3 | 0.188 | 0 | [1-3, 10] |
| Rv2875 | *mpt70* | Major secreted immunogenic protein | 3 | 0.309 | 1 | [2, 3] |
| Rv2877c | *merT* | Possible conserved integral membrane protein | 3 | 0.997 | 7 |  |
| Rv2878c | *mpt53* | Soluble secreted antigen mpt53 precursor | 3 | 0.334 | 1 | [2, 7] |
| Rv2881c | *cdsA* | Possible integral membrane phosphatidate cytidylyltransferase | 1 | 0.743 | 7 |  |
| Rv2882c | *frr* | Ribosome recycling factor | 2 | -0.601 | 0 | [1, 3, 7] |
| Rv2883c | *pyrH* | Possible uridylate kinase | 7 | 0.192 | 0 | [1, 10] |
| Rv2887 | *-* | Possible transcriptional regulatory protein | 9 | 0.061 | 0 |  |
| Rv2888c | *amiC* | Possible amidase | 7 | 0.018 | 0 | [1, 10] |
| Rv2889c | *tsf* | Possible elongation factor | 2 | -0.073 | 0 | [1, 3, 6, 7, 9] |
| Rv2890c | *rpsB* | Possible 30s ribosomal protein | 2 | -0.284 | 0 | [1, 6, 7, 9, 10] |
| Rv2893 | *-* | Possible oxidoreductase | 7 | 0.079 | 0 | [3, 6] |
| Rv2895c | *viuB* | Possible mycobactin utilization protein | 7 | -0.13 | 0 | [6] |
| Rv2901c | *-* | Conserved hypothetical protein | 10 | -0.535 | 0 | [1, 9] |
| Rv2903c | *lepB* | Possible signal peptidase I | 3 | -0.23 | 1 | [5, 10] |
| Rv2909c | *rpsP* | Possible 30s ribosomal protein s16 | 2 | -0.618 | 0 | [1] |
| Rv2912c | *-* | Possible transcriptional regulatory protein | 9 | 0.099 | 0 | [6] |
| Rv2913c | *-* | Possible d-amino acid aminohydrolase | 7 | -0.166 | 0 | [6] |
| Rv2915c | *-* | Conserved hypothetical protein | 10 | -0.169 | 0 |  |
| Rv2919c | *glnB* | Possible nitrogen regulatory protein p-II | 9 | -0.047 | 0 | [1] |
| Rv2920c | *amt* | Possible ammonium-transport integral membrane protein | 3 | 0.736 | 11 |  |
| Rv2921c | *ftsY* | Possible cell division protein | 3 | 0.103 | 1 | [1, 3, 10] |
| Rv2923c | *-* | Conserved hypothetical protein | 10 | -0.132 | 0 | [6] |
| Rv2925c | *rnc* | Possible ribonuclease iii | 2 | -0.063 | 0 |  |
| Rv2926c | *-* | Conserved hypothetical protein | 10 | -0.145 | 0 |  |
| Rv2935 | *ppsE* | Phenolpthiocerol synthesis type-i polyketide synthase | 1 | -0.053 | 0 | [6, 10] |
| Rv2937 | *drrB* | Possible daunorubicin-dim-transport integral membrane protein abc transporter | 3 | 0.766 | 6 |  |
| Rv2938 | *drrC* | Possible daunorubicin-dim-transport integral membrane protein abc transporter | 3 | 0.952 | 6 |  |
| Rv2940c | *mas* | Possible multifunctional mycocerosic acid synthase membrane-associated | 1 | -0.053 | 0 | [1, 3, 6, 10] |
| Rv2941 | *fadD28* | Fatty-acid-CoA ligase | 1 | -0.134 | 0 | [1, 3] |
| Rv2942 | *mmpL7* | Conserved transmembrane transport protein | 3 | 0.392 | 12 | [10] |
| Rv2945c | *lppX* | Possible conserved lipoprotein | 3 | 0.013 | 1 | [1-3, 5, 6, 10] |
| Rv2951c | *-* | Possible oxidoreductase | 7 | -0.013 | 0 | [1, 5-7, 9, 10] |
| Rv2952 | *-* | Possible methyltransferase | 7 | -0.439 | 0 | [1, 6, 9] |
| Rv2953 | *-* | Conserved hypothetical protein | 10 | -0.166 | 0 | [1, 3, 5-7] |
| Rv2955c | *-* | Conserved hypothetical protein | 10 | -0.156 | 0 | [1, 5, 6, 9] |
| Rv2959c | *-* | Possible methyltransferase | 7 | -0.295 | 0 | [1, 3, 10] |
| Rv2963 | *-* | Possible integral membrane protein | 3 | 0.783 | 9 |  |
| Rv2965c | *kdtB* | Possible phosphopantetheine adenylyltransferase | 3 | 0.073 | 0 |  |
| Rv2967c | *pca* | Possible pyruvate carboxylase | 7 | -0.026 | 0 | [6] |
| Rv2969c | *-* | Possible conserved membrane or secreted protein | 3 | 0.097 | 1 | [1, 3, 5, 9, 10] |
| Rv2970c | *lipN* | Possible lipase/esterase | 7 | 0.001 | 0 | [1, 5, 7] |
| Rv2971 | *-* | Possible oxidoreductase | 7 | -0.081 | 0 | [1, 3, 7] |
| Rv2976c | *ung* | Possible uracil-DNA glycosylase | 2 | 0.009 | 0 |  |
| Rv2977c | *thiL* | Possible thiamine-monophosphate kinase | 7 | 0.086 | 0 |  |
| Rv2980 | *-* | Possible conserved secreted protein | 3 | 0.135 | 1 |  |
| Rv2982c | *gpdA2* | Possible glycerol-3-phosphate dehydrogenase [NAD(P)+] | 1 | 0.296 | 0 | [1, 3] |
| Rv2986c | *hupB* | Possible DNA-binding protein hu homolog | 2 | -0.556 | 0 | [1, 6] |
| Rv2987c | *leuD* | Possible 3-isopropylmalate dehydratase | 7 | 0.072 | 0 | [1, 3] |
| Rv2991 | *-* | Conserved hypothetical protein | 10 | -0.425 | 0 |  |
| Rv2992c | *gltS* | Possible glutamyl-tRNA synthetase | 2 | -0.169 | 0 | [3, 6] |
| Rv2993c | *-* | Possible 2-hydroxyhepta-2,4-diene-1,7-dioate isomerase | 7 | 0.052 | 0 |  |
| Rv2994 | *-* | Possible conserved integral membrane protein | 3 | 0.423 | 8 | [2] |
| Rv2995c | *leuB* | Possible 3-isopropylmalate dehydrogenase | 7 | 0.121 | 0 | [10] |
| Rv2996c | *serA1* | Possible d-3-phosphoglycerate dehydrogenase | 7 | 0.334 | 0 | [1, 3, 6, 10] |
| Rv2999 | *lppY* | Possible conserved lipoprotein | 3 | -0.11 | 0 | [1, 3, 5, 6, 10] |
| Rv3001c | *ilvC* | Possible ketol-acid reductoisomerase | 7 | -0.144 | 0 | [1, 3, 6, 10] |
| Rv3002c | *ilvN* | Possible acetolactate synthase | 7 | -0.043 | 0 | [1] |
| Rv3004 | *cfp6* | Low molecular weight protein antigen | 3 | 0.322 | 1 | [2] |
| Rv3006 | *lppZ* | Possible conserved lipoprotein | 3 | -0.065 | 0 | [1-3, 5, 10] |
| Rv3009c | *gatB* | Possible glutamyl-tRNA(gln) amidotransferase | 2 | -0.137 | 0 | [1, 7] |
| Rv3010c | *pfkA* | Possible 6-phosphofructokinase | 7 | -0.102 | 0 | [6] |
| Rv3011c | *gatA* | Possible glutamyl-tRNA(gln) amidotransferase | 2 | 0.083 | 0 |  |
| Rv3012c | *gatC* | Possible glutamyl-tRNA(gln) amidotransferase | 2 | -0.181 | 0 |  |
| Rv3013 | *-* | Conserved hypothetical protein | 10 | 0.117 | 0 |  |
| Rv3025c | *iscS* | Possible cysteine desulfurase | 7 | 0.096 | 0 |  |
| Rv3028c | *fixB* | Possible electron transfer flavoprotein | 7 | 0.358 | 0 | [1, 3, 5-7] |
| Rv3029c | *fixA* | Possible electron transfer flavoprotein | 7 | -0.108 | 0 | [1, 3, 5-7] |
| Rv3033 | *-* | Hypothetical protein | 10 | -0.098 | 0 | [1-3, 5] |
| Rv3034c | *-* | Possible transferase | 7 | -0.088 | 0 | [6] |
| Rv3035 | *-* | Conserved hypothetical protein | 10 | -0.014 | 0 | [5] |
| Rv3039c | *echA17* | Possible enoyl-CoA hydratase | 1 | 0.03 | 0 | [1, 6] |
| Rv3043c | *ctaD* | Possible cytochrome c oxidase polypeptide I | 7 | 0.582 | 12 | [3, 5, 10] |
| Rv3045 | *adhC* | Possible NADp-dependent alcohol dehydrogenase | 7 | 0.039 | 0 | [1, 3, 6, 7] |
| Rv3046c | *-* | Conserved hypothetical protein | 10 | -0.24 | 0 | [3] |
| Rv3048c | *nrdF2* | Ribonucleoside-diphosphate reductase | 2 | -0.312 | 0 |  |
| Rv3058c | *-* | Possible transcriptional regulatory protein | 9 | -0.222 | 0 | [1] |
| Rv3066 | *-* | Possible transcriptional regulatory protein | 9 | -0.325 | 0 |  |
| Rv3068c | *pgmA* | Possible phosphoglucomutase | 7 | -0.105 | 0 | [6] |
| Rv3069 | *-* | Possible conserved transmembrane protein | 3 | 0.502 | 4 | [3] |
| Rv3075c | *-* | Conserved hypothetical protein | 10 | -0.123 | 0 | [1, 7] |
| Rv3079c | *-* | Conserved hypothetical protein | 10 | -0.083 | 0 |  |
| Rv3086 | *adhD* | Possible zinc-type alcohol dehydrogenase | 7 | 0.188 | 0 | [1] |
| Rv3090 | *-* | Hypothetical alanine and valine rich protein | 10 | -0.006 | 2 | [1, 3, 5, 10] |
| Rv3091 | *-* | Conserved hypothetical protein | 10 | -0.117 | 0 | [1, 6, 10] |
| Rv3092c | *-* | Possible conserved integral membrane protein | 3 | 0.816 | 4 | [6] |
| Rv3099c | *-* | Conserved hypothetical protein | 10 | -0.073 | 0 | [1, 3, 6] |
| Rv3101c | *ftsX* | Putative cell division protein | 3 | 0.386 | 4 | [1, 3, 6, 10] |
| Rv3102c | *ftsE* | Putative cell division ATP-binding protein | 3 | -0.251 | 0 | [1] |
| Rv3104c | *-* | Possible conserved transmembrane protein | 3 | 0.354 | 3 | [3] |
| Rv3105c | *prfB* | Possible peptide chain release factor | 2 | -0.458 | 0 | [1] |
| Rv3107c | *agpS* | Possible alkyldihydroxyacetonephosphate synthase | 1 | -0.12 | 0 |  |
| Rv3133c | *devR* | Two component transcriptional regulatory protein | 9 | -0.017 | 0 | [1, 3, 6, 7, 10] |
| Rv3136 | *PPE51* | PPE family protein | 6 | 0.333 | 0 |  |
| Rv3139 | *fadE24* | Possible acyl-CoA dehydrogenase | 1 | -0.06 | 0 | [1, 3, 5, 6, 9] |
| Rv3140 | *fadE23* | Possible acyl-CoA dehydrogenase | 1 | -0.152 | 0 | [1, 3, 5] |
| Rv3141 | *fadB4* | Possible NADPH quinone oxidoreductase | 1 | 0.18 | 0 | [1, 3] |
| Rv3143 | *-* | Possible response regulator | 9 | -0.02 | 0 | [1] |
| Rv3145 | *nuoA* | Possible NADH dehydrogenase I | 7 | 0.813 | 3 | [3] |
| Rv3146 | *nuoB* | Possible NADH dehydrogenase I | 7 | 0.155 | 0 | [1, 3] |
| Rv3147 | *nuoC* | Possible NADH dehydrogenase I | 7 | -0.693 | 0 | [1, 6, 7] |
| Rv3148 | *nuoD* | Possible NADH dehydrogenase I | 7 | -0.047 | 0 | [1] |
| Rv3149 | *nuoE* | Possible NADH dehydrogenase I | 7 | -0.325 | 0 | [1] |
| Rv3150 | *nuoF* | Possible NADH dehydrogenase I | 7 | -0.123 | 0 |  |
| Rv3151 | *nuoG* | Possible NADH dehydrogenase I | 7 | -0.024 | 0 | [1, 6] |
| Rv3152 | *nuoH* | Possible NADH dehydrogenase I | 7 | 0.608 | 9 |  |
| Rv3153 | *nuoI* | Possible NADH dehydrogenase I | 7 | -0.635 | 0 | [6] |
| Rv3156 | *nuoL* | Possible NADH dehydrogenase I | 7 | 0.748 | 16 |  |
| Rv3157 | *nuoM* | Possible NADH dehydrogenase I | 7 | 0.762 | 14 | [3] |
| Rv3158 | *nuoN* | Possible NADH dehydrogenase I | 7 | 0.846 | 14 | [1-3, 5, 10] |
| Rv3161c | *-* | Possible dioxygenase | 7 | -0.126 | 0 | [1] |
| Rv3165c | *-* | Hypothetical protein | 10 | -0.173 | 2 | [6] |
| Rv3168 | *-* | Conserved hypothetical protein | 10 | -0.098 | 0 | [1, 3] |
| Rv3169 | *-* | Conserved hypothetical protein | 10 | -0.376 | 0 | [1] |
| Rv3170 | *aofH* | Possible flavin-containing monoamine oxidase | 7 | -0.189 | 0 | [6] |
| Rv3179 | *-* | Conserved hypothetical protein | 10 | -0.151 | 0 |  |
| Rv3193c | *-* | Possible conserved transmembrane protein | 3 | 0.061 | 7 | [1-3, 10] |
| Rv3194c | *-* | Possible conserved secreted protein | 3 | 0.116 | 1 | [2, 10] |
| Rv3196A | *-* | Hypothetical protein | 10 | -0.652 | 0 |  |
| Rv3200c | *-* | Possible transmembrane cation transporter | 3 | 0.216 | 3 | [1, 6, 10] |
| Rv3205c | *-* | Conserved hypothetical protein | 10 | 0.027 | 0 | [1, 7] |
| Rv3210c | *-* | Conserved hypothetical protein | 10 | -0.088 | 0 | [3] |
| Rv3212 | *-* | Conserved hypothetical alanine valine rich protein | 10 | -0.009 | 1 | [6, 10] |
| Rv3214 | *gpm2* | Possible phosphoglycerate mutase | 7 | -0.094 | 0 |  |
| Rv3223c | *sigH* | Alternative RNA polymerase sigma-e factor | 2 | -0.575 | 0 | [1, 7] |
| Rv3224 | *-* | Possible iron-regulated short-chain dehydrogenase/reductase | 7 | 0.102 | 0 | [1, 6, 7] |
| Rv3227 | *aroA* | 3-phosphoshikimate 1-carboxyvinyltransferase | 7 | 0.063 | 0 | [6, 7] |
| Rv3231c | *-* | Conserved hypothetical protein | 10 | 0.3 | 0 |  |
| Rv3236c | *kefB* | Possible conserved integral membrane transport protein | 3 | 1.012 | 10 |  |
| Rv3237c | *-* | Conserved hypothetical protein | 10 | -0.079 | 0 | [1] |
| Rv3238c | *-* | Possible conserved integral membrane protein | 3 | 0.519 | 5 |  |
| Rv3240c | *secA1* | Possible preprotein translocase | 3 | -0.468 | 0 | [1-3, 6] |
| Rv3241c | *-* | Conserved hypothetical protein | 2 | -0.662 | 0 | [1, 3] |
| Rv3243c | *-* | hypothetical protein | 10 | 0.02 | 0 |  |
| Rv3244c | *lpqB* | Possible conserved lipoprotein | 3 | -0.03 | 0 | [2, 3, 5, 6, 10] |
| Rv3248c | *sahH* | Possible adenosylhomocysteinase | 7 | -0.26 | 0 | [1-3, 6, 7] |
| Rv3255c | *manA* | Possible mannose-6-phosphate isomerase | 7 | 0.025 | 0 | [1, 6] |
| Rv3264c | *manB* | D-alpha-d-mannose-1-phosphate guanylyltransferase | 3 | 0.065 | 0 | [1, 6] |
| Rv3269 | *-* | Conserved hypothetical protein | 0 | -0.037 | 0 | [1, 3, 5, 7] |
| Rv3270 | *ctpC* | Possible metal cation-transporting p-type ATPase c | 3 | 0.204 | 0 | [1, 6, 10] |
| Rv3273 | *-* | Possible transmembrane carbonic anhydrase | 7 | 0.476 | 10 | [1, 5, 6, 10] |
| Rv3274c | *fadE25* | Possible acyl-CoA dehydrogenase | 1 | -0.127 | 0 | [1, 6, 7, 10] |
| Rv3275c | *purE* | Possible phosphoribosylaminoimidazole carboxylase catalytic subunit | 7 | 0.405 | 0 |  |
| Rv3276c | *purK* | Possible phosphoribosylaminoimidazole carboxylase ATPase subunit | 7 | 0.033 | 0 | [6] |
| Rv3277 | *-* | Possible conserved transmembrane protein | 3 | 0.286 | 4 | [3] |
| Rv3278c | *-* | Possible conserved transmembrane protein | 3 | 0.267 | 2 | [1, 3, 5] |
| Rv3280 | *accD5* | Possible propionyl-CoA carboxylase beta chain | 1 | -0.16 | 0 | [1, 3, 7] |
| Rv3281 | *-* | Conserved hypothetical protein | 10 | -0.898 | 0 |  |
| Rv3283 | *sseA* | Possible thiosulfate sulfurtransferase | 7 | -0.338 | 0 | [1, 3, 6, 7] |
| Rv3287c | *rsbW* | Anti-sigma factor | 2 | -0.112 | 0 |  |
| Rv3295 | *-* | Possible transcriptional regulatory protein | 9 | -0.25 | 0 | [1] |
| Rv3298c | *lpqC* | Possible esterase lipoprotein | 3 | 0.126 | 0 | [10] |
| Rv3299c | *atsB* | Possible arylsulfatase | 7 | -0.254 | 0 | [6] |
| Rv3302c | *glpD2* | Possible glycerol-3-phosphate dehydrogenase | 7 | 0.076 | 0 | [1, 10] |
| Rv3305c | *amiA1* | Possible n-acyl-l-amino acid amidohydrolase | 7 | 0.114 | 0 | [3] |
| Rv3306c | *amiB1* | Possible amidohydrolase | 7 | 0.157 | 0 |  |
| Rv3307 | *deoD* | Possible purine nucleoside phosphorylase | 7 | 0.256 | 0 |  |
| Rv3309c | *upp* | Possible uracil phosphoribosyltransferase | 7 | 0.197 | 0 | [6] |
| Rv3310 | *sapM* | Acid phosphatase | 3 | 0.066 | 1 | [2] |
| Rv3311 | *-* | Conserved hypothetical protein | 10 | -0.222 | 0 | [1, 3] |
| Rv3313c | *add* | Possible adenosine deaminase | 7 | 0.056 | 0 | [1] |
| Rv3314c | *deoA* | Possible thymidine phosphorylase | 7 | 0.079 | 0 |  |
| Rv3315c | *cdd* | Possible cytidine deaminase | 7 | 0.135 | 0 | [1] |
| Rv3316 | *sdhC* | Possible succinate dehydrogenase | 7 | 0.897 | 3 |  |
| Rv3317 | *sdhD* | Possible succinate dehydrogenase | 7 | 0.135 | 3 |  |
| Rv3318 | *sdhA* | Possible succinate dehydrogenase | 7 | -0.249 | 0 | [1, 6] |
| Rv3319 | *sdhB* | Possible succinate dehydrogenase | 7 | -0.154 | 0 | [1, 6] |
| Rv3332 | *nagA* | Possible n-acetylglucosamine-6-phosphate deacetylase | 3 | 0.316 | 0 |  |
| Rv3335c | *-* | Possible conserved integral membrane protein | 3 | 0.78 | 6 |  |
| Rv3336c | *trpS* | Possible tryptophanyl-tRNA synthetase | 2 | -0.104 | 0 | [1] |
| Rv3339c | *icd1* | Possible isocitrate dehydrogenase [NADP] | 7 | -0.221 | 0 | [1, 6] |
| Rv3340 | *metC* | Possible o-acetylhomoserine sulfhydrylase | 7 | 0.068 | 0 |  |
| Rv3356c | *folD* | Possible bifunctional protein fold | 7 | 0.051 | 0 | [1, 2, 7] |
| Rv3361c | *-* | Conserved hypothetical protein | 10 | -0.081 | 0 |  |
| Rv3362c | *-* | Possible ATP/GTP-binding protein | 10 | 0.209 | 0 |  |
| Rv3364c | *-* | Conserved hypothetical protein | 10 | 0.373 | 0 |  |
| Rv3368c | *-* | Possible oxidoreductase | 7 | -0.224 | 0 | [3] |
| Rv3369 | *-* | Conserved hypothetical protein | 10 | -0.039 | 0 | [1, 2] |
| Rv3384c | *-* | Conserved hypothetical protein with pin domain | 0 | -0.045 | 0 | [1, 10] |
| Rv3389c | *-* | Double hotdog hydratase | 7 | 0.02 | 0 | [1, 6, 7] |
| Rv3390 | *lpqD* | Possible conserved lipoprotein | 3 | -0.087 | 1 | [1, 3, 9, 10] |
| Rv3392c | *cmaA1* | Cyclopropane-fatty-acyl-phospholipid synthase 1 | 1 | -0.117 | 0 | [1, 7] |
| Rv3393 | *iunH* | Possible nucleoside hydrolase | 7 | 0.098 | 0 |  |
| Rv3396c | *guaA* | Possible gmp synthase | 7 | 0.049 | 0 | [1, 7] |
| Rv3400 | *-* | Possible hydrolase | 7 | -0.215 | 0 | [7] |
| Rv3401 | *-* | Conserved hypothetical protein | 7 | -0.266 | 0 | [1, 7, 10] |
| Rv3409c | *choD* | Cholesterol oxidase | 1 | -0.3 | 0 | [1, 10] |
| Rv3410c | *guaB3* | Possible inosine-5'-monophosphate dehydrogenase | 7 | 0.202 | 0 | [1] |
| Rv3417c | *groEL1* | 60 kDa chaperonin 1 | 0 | 0.11 | 0 | [1, 3, 5-7, 10] |
| Rv3418c | *groES* | 10 kDa chaperonin | 0 | -0.325 | 0 | [1-3, 6, 7] |
| Rv3423c | *alr* | Alanine racemase | 7 | 0.02 | 0 | [1] |
| Rv3432c | *gadB* | Possible glutamate decarboxylase | 7 | -0.079 | 0 | [1] |
| Rv3433c | *-* | Conserved hypothetical protein | 10 | 0.382 | 0 |  |
| Rv3435c | *-* | Possible conserved transmembrane protein | 3 | 0.431 | 4 | [6] |
| Rv3436c | *glmS* | Possible glucosamine | 7 | -0.022 | 0 | [1, 3, 6] |
| Rv3438 | *-* | Conserved hypothetical protein | 10 | 0.116 | 0 | [1] |
| Rv3443c | *rplM* | Possible 50s ribosomal protein | 2 | -0.434 | 0 | [1, 6, 9] |
| Rv3451 | *cut3* | Possible cutinase precursor | 3 | 0.246 | 1 |  |
| Rv3455c | *truA* | Possible tRNA pseudouridine synthase a | 2 | -0.141 | 0 | [1, 6] |
| Rv3456c | *rplQ* | Possible 50s ribosomal protein l17 | 2 | -0.672 | 0 | [1, 6, 9, 10] |
| Rv3457c | *rpoA* | Possible DNA-directed RNA polymerase | 2 | -0.196 | 0 | [1, 3, 5-7] |
| Rv3458c | *rpsD* | Possible 30s ribosomal protein s4 | 2 | -0.722 | 0 | [1, 3, 6, 9, 10] |
| Rv3459c | *rpsK* | Possible 30s ribosomal protein s11 | 2 | -0.709 | 0 | [1, 10] |
| Rv3462c | *infA* | Possible translation initiation factor if-1 | 2 | -0.377 | 0 | [1, 6] |
| Rv3463 | *-* | Conserved hypothetical protein | 10 | -0.1 | 0 | [6, 7] |
| Rv3465 | *rmlC* | Dtdp-4-dehydrorhamnose 3,5-epimerase | 7 | -0.177 | 0 |  |
| Rv3472 | *-* | Conserved hypothetical protein | 10 | -0.194 | 0 |  |
| Rv3476c | *kgtP* | Possible dicarboxylic acid transport integral membrane protein | 3 | 0.458 | 11 | [6] |
| Rv3478 | *PPE60* | PE family protein | 6 | 0.276 | 0 |  |
| Rv3479 | *-* | Possible transmembrane protein | 3 | 0.018 | 3 | [6, 10] |
| Rv3481c | *-* | Possible integral membrane protein | 3 | 0.993 | 6 | [3] |
| Rv3483c | *-* | Possible exported protein | 3 | -0.126 | 1 | [10] |
| Rv3484 | *cpsA* | Possible conserved protein | 10 | -0.273 | 1 | [2, 6] |
| Rv3486 | *-* | Conserved hypothetical protein | 10 | 0.674 | 0 |  |
| Rv3489 | *-* | Hypothetical protein | 10 | -0.194 | 0 | [3] |
| Rv3490 | *otsA* | Possible alpha, alpha-trehalose-phosphate synthase | 0 | -0.26 | 0 | [1, 6] |
| Rv3497c | *mce4C* | Mce-family protein | 0 | 0.019 | 1 | [10] |
| Rv3500c | *yrbE4B* | Conserved hypothetical integral membrane protein | 0 | 0.814 | 5 |  |
| Rv3501c | *yrbE4A* | Conserved hypothetical integral membrane protein | 0 | 0.932 | 6 |  |
| Rv3504 | *fadE26* | Possible acyl-CoA dehydrogenase | 1 | -0.247 | 0 |  |
| Rv3505 | *fadE27* | Possible acyl-CoA dehydrogenase | 1 | 0.062 | 0 | [6] |
| Rv3509c | *ilvX* | Possible acetohydroxyacid synthase | 7 | 0.32 | 0 | [1, 2, 7] |
| Rv3510c | *-* | Conserved hypothetical protein | 10 | -0.173 | 0 |  |
| Rv3516 | *echA19* | Possible enoyl-CoA hydratase | 1 | -0.141 | 0 |  |
| Rv3519 | *-* | Hypothetical protein | 10 | -0.076 | 0 | [1, 9, 10] |
| Rv3520c | *-* | Possible coenzyme f420-dependent oxidoreductase | 7 | -0.057 | 0 | [1] |
| Rv3525c | *-* | Possible siderophore-binding protein | 7 | 0.49 | 0 |  |
| Rv3528c | *-* | Hypothetical protein | 10 | -0.339 | 0 | [1] |
| Rv3534c | *hsaF* | Possible 4-hydroxy-2-oxovalerate aldolase | 7 | 0.073 | 0 |  |
| Rv3541c | *-* | Conserved hypothetical protein | 10 | 0.143 | 0 |  |
| Rv3546 | *fadA5* | Possible acetyl-CoA acetyltransferase | 1 | -0.041 | 0 | [1] |
| Rv3547 | *-* | Conserved hypothetical protein | 10 | -0.544 | 0 | [1, 6, 9, 10] |
| Rv3552 | *-* | Possible CoA-transferase | 7 | -0.193 | 0 |  |
| Rv3556c | *fadA6* | Possible acetyl-CoA acetyltransferase | 1 | -0.058 | 0 | [1, 3] |
| Rv3563 | *fadE32* | Possible acyl-CoA dehydrogenase | 1 | 0.15 | 0 | [3] |
| Rv3565 | *aspB* | Possible aspartate aminotransferase | 7 | 0.205 | 0 |  |
| Rv3566c | *nat* | Arylamine n-acetyltransferase | 7 | -0.873 | 0 | [6] |
| Rv3568c | *hsaC* | 3,4-dhsa dioxygenase | 7 | -0.272 | 0 |  |
| Rv3569c | *hsaD* | 4,9-dhsa hydrolase | 7 | -0.108 | 0 | [1, 6] |
| Rv3570c | *hsaA* | Possible oxidoreductase | 7 | -0.225 | 0 | [1] |
| Rv3571 | *hmp* | Possible hemoglobine-related protein | 7 | 0.001 | 0 |  |
| Rv3572 | *-* | Hypothetical protein | 10 | -0.068 | 0 | [2] |
| Rv3575c | *-* | Transcriptional regulatory protein | 9 | -0.028 | 0 | [1] |
| Rv3576 | *lppH* | Possible conserved lipoprotein | 3 | -0.068 | 0 | [3, 9] |
| Rv3580c | *cysS1* | Cysteinyl-tRNA synthetase 1 | 2 | -0.352 | 0 | [6] |
| Rv3581c | *ispF* | Possible 2c-methyl-d-erythritol 2,4-cyclodiphosphate synthase | 7 | 0.303 | 0 | [3] |
| Rv3582c | *ispD* | 4-diphosphocytidyl-2c-methyl-d-erythritol synthase | 7 | 0.29 | 0 | [1, 3] |
| Rv3583c | *-* | Possible transcription factor | 9 | -0.313 | 0 |  |
| Rv3584 | *lpqE* | Possible conserved lipoprotein | 3 | 0.209 | 1 | [1-3, 5, 10] |
| Rv3587c | *-* | Possible conserved membrane protein | 3 | -0.116 | 1 | [2, 3] |
| Rv3588c | *canB* | Beta-carbonic anhydrase | 7 | 0.156 | 0 |  |
| Rv3591c | *-* | Possible hydrolase | 7 | -0.224 | 0 |  |
| Rv3596c | *clpC1* | Possible ATP-dependent protease ATP-binding subunit | 7 | -0.362 | 0 | [1, 3, 6, 7] |
| Rv3597c | *lsr2* | Possible iron-regulated lsr2 protein precursor | 10 | -0.659 | 0 | [1, 6, 7, 9, 10] |
| Rv3598c | *lysS* | Lysyl-tRNA synthetase 1 | 2 | -0.177 | 0 |  |
| Rv3600c | *-* | Conserved hypothetical protein | 10 | 0.161 | 0 | [1] |
| Rv3607c | *folB* | Possible dihydroneopterin aldolase | 7 | -0.139 | 0 |  |
| Rv3614c | *-* | Conserved hypothetical protein | 10 | -0.384 | 0 | [1] |
| Rv3615c | *-* | Conserved hypothetical protein | 10 | -0.012 | 0 | [3] |
| Rv3616c | *-* | Conserved hypothetical alanine and glycine rich protein | 10 | 0.24 | 0 | [1, 3, 5, 10] |
| Rv3618 | *-* | Possible monooxygenase | 7 | -0.139 | 0 |  |
| Rv3623 | *lpqG* | Possible conserved lipoprotein | 3 | -0.055 | 0 | [1, 6, 10] |
| Rv3627c | *-* | Conserved hypothetical protein | 10 | 0.147 | 1 | [2, 10] |
| Rv3628 | *ppa* | Inorganic pyrophosphatase | 7 | -0.403 | 0 | [1, 6, 7] |
| Rv3632 | *-* | Possible conserved membrane protein | 3 | 0.459 | 3 |  |
| Rv3633 | *-* | Conserved hypothetical protein | 10 | -0.216 | 0 | [1, 3] |
| Rv3634c | *galE1* | UDP-glucose 4-epimerase | 7 | -0.133 | 0 | [1, 7] |
| Rv3646c | *topA* | DNA topoisomerase i | 2 | -0.474 | 0 | [1, 6] |
| Rv3666c | *dppA* | Possible periplasmic dipeptide-binding lipoprotein | 3 | -0.182 | 1 | [3] |
| Rv3669 | *-* | Possible conserved transmembrane protein | 3 | 0.277 | 2 | [10] |
| Rv3670 | *ephE* | Possible epoxide hydrolase | 0 | -0.25 | 0 | [6] |
| Rv3671c | *-* | Possible membrane-associated serine protease | 7 | 0.528 | 4 | [2, 10] |
| Rv3673c | *-* | Possible membrane-anchored thioredoxin-like protein | 7 | 0.159 | 1 |  |
| Rv3675 | *-* | Possible membrane protein | 3 | -0.095 | 1 | [6] |
| Rv3676 | *-* | Possible transcriptional regulatory protein | 9 | -0.271 | 0 | [1, 7, 10] |
| Rv3678c | *-* | Conserved hypothetical protein | 10 | 0.47 | 0 | [1] |
| Rv3682 | *ponA2* | Possible bifunctional membrane-associated penicillin-binding protein | 3 | -0.119 | 1 | [1-3, 5, 6] |
| Rv3683 | *-* | Conserved hypothetical protein | 10 | -0.054 | 1 | [1, 7] |
| Rv3684 | *-* | Possible lyase | 7 | -0.147 | 0 | [1, 6, 7] |
| Rv3687c | *rsfB* | Anti-anti-sigma factor | 2 | 0.44 | 0 |  |
| Rv3688c | *-* | Conserved hypothetical protein | 10 | -0.351 | 0 | [1] |
| Rv3689 | *-* | Possible conserved transmembrane protein | 3 | 0.575 | 6 |  |
| Rv3690 | *-* | Possible conserved membrane protein | 3 | -0.278 | 1 |  |
| Rv3691 | *-* | Conserved hypothetical protein | 10 | -0.002 | 0 | [1] |
| Rv3692 | *moxR2* | Possible methanol dehydrogenase transcriptional regulatory protein | 9 | -0.029 | 0 | [1, 6, 10] |
| Rv3693 | *-* | Possible conserved membrane protein | 3 | 0.004 | 1 | [2, 6] |
| Rv3694c | *-* | Possible conserved transmembrane protein | 3 | 0.526 | 6 |  |
| Rv3695 | *-* | Possible conserved membrane protein | 3 | 0.25 | 3 | [1] |
| Rv3696c | *glpK* | Possible glycerol kinase | 7 | -0.098 | 0 |  |
| Rv3699 | *-* | Conserved hypothetical protein | 10 | -0.096 | 0 | [1, 7] |
| Rv3705c | *-* | Conserved hypothetical protein | 10 | 0.071 | 0 | [2] |
| Rv3708c | *asd* | Aspartate-semialdehyde dehydrogenase | 7 | 0.082 | 0 | [1] |
| Rv3709c | *ask* | Aspartokinase | 7 | 0.099 | 0 | [1, 3] |
| Rv3710 | *leuA* | 2-isopropylmalate synthase | 7 | -0.284 | 0 | [2, 6, 7] |
| Rv3713 | *cobQ2* | Possible cobyric acid synthase | 7 | 0.112 | 0 |  |
| Rv3716c | *-* | Conserved hypothetical protein | 10 | -0.078 | 0 | [1, 7] |
| Rv3718c | *-* | Conserved hypothetical protein | 10 | -0.181 | 0 | [1] |
| Rv3719 | *-* | Conserved hypothetical protein | 10 | -0.313 | 0 | [6, 9, 10] |
| Rv3720 | *-* | Possible fatty acid synthase | 1 | -0.362 | 0 | [1, 10] |
| Rv3722c | *-* | Conserved hypothetical protein | 10 | -0.053 | 0 | [1, 2] |
| Rv3723 | *-* | Possible conserved transmembrane protein | 3 | -0.178 | 4 | [1, 3, 5, 10] |
| Rv3726 | *-* | Possible dehydrogenase | 7 | 0.183 | 0 | [1, 10] |
| Rv3731 | *ligC* | Possible ATP-dependent DNA ligase | 2 | -0.337 | 0 |  |
| Rv3732 | *-* | Conserved hypothetical protein | 10 | 0.15 | 2 | [10] |
| Rv3734c | *-* | Conserved hypothetical protein | 10 | -0.018 | 0 | [1, 3, 6, 10] |
| Rv3747 | *-* | Conserved hypothetical protein | 10 | 0.385 | 0 |  |
| Rv3753c | *-* | Conserved hypothetical protein | 10 | -0.068 | 0 | [1] |
| Rv3754 | *tyrA* | Prephenate dehydrogenase | 7 | 0.176 | 0 |  |
| Rv3755c | *-* | Conserved hypothetical protein | 10 | -0.2 | 0 |  |
| Rv3756c | *proZ* | Possible osmoprotectant | 0 | 0.933 | 5 |  |
| Rv3758c | *proV* | Possible osmoprotectant | 0 | 0.046 | 0 | [3, 6] |
| Rv3763 | *lpqH* | 19 kDa lipoprotein antigen precursor | 3 | 0.112 | 0 | [1, 3, 5, 7, 9, 10] |
| Rv3764c | *tcrY* | Possible two component sensor kinase | 9 | -0.013 | 1 | [6] |
| Rv3767c | *-* | Conserved hypothetical protein | 10 | -0.265 | 0 | [1] |
| Rv3768 | *-* | Hypothetical protein | 10 | -0.156 | 0 |  |
| Rv3772 | *hisC2* | Possible histidinol-phosphate aminotransferase | 7 | 0.141 | 0 | [7] |
| Rv3774 | *echA21* | Possible enoyl-CoA hydratase | 1 | 0.065 | 0 | [1, 5, 6] |
| Rv3775 | *lipE* | Possible lipase | 7 | -0.167 | 0 | [1] |
| Rv3777 | *-* | Possible oxidoreductase | 7 | 0.339 | 0 | [1, 6] |
| Rv3780 | *-* | Conserved hypothetical protein | 10 | -0.366 | 0 | [1, 3] |
| Rv3781 | *rfbE* | Possible o-antigen/lipopolysaccharide transport ATP-binding protein abc transporter | 3 | -0.093 | 0 | [1] |
| Rv3783 | *rfbD* | Possible o-antigen/lipopolysaccharide transport integral membrane protein abc transporter | 3 | 0.51 | 6 |  |
| Rv3790 | *-* | Possible oxidoreductase | 7 | -0.102 | 0 | [1, 6] |
| Rv3791 | *-* | Possible short-chain type dehydrogenase/reductase | 7 | 0.157 | 0 | [1, 3, 10] |
| Rv3792 | *-* | Possible conserved transmembrane protein | 3 | 0.438 | 13 | [6] |
| Rv3793 | *embC* | Integral membrane indolylacetylinositol arabinosyltransferase | 3 | 0.242 | 13 | [3, 6] |
| Rv3794 | *embA* | Integral membrane indolylacetylinositol arabinosyltransferase | 3 | 0.336 | 13 | [3, 6, 10] |
| Rv3795 | *embB* | Integral membrane indolylacetylinositol arabinosyltransferase | 3 | 0.304 | 12 | [3, 6] |
| Rv3796 | *atsH* | Conserved hypothetical protein | 10 | -0.011 | 0 | [3] |
| Rv3797 | *fadE35* | Possible acyl-CoA dehydrogenase | 1 | -0.252 | 0 |  |
| Rv3800c | *pks13* | Polyketide synthase | 1 | -0.186 | 0 | [1, 3, 6] |
| Rv3801c | *fadD32* | Possible fatty-acid-CoA ligase | 1 | -0.185 | 0 | [1, 6, 7, 10] |
| Rv3802c | *-* | Possible conserved membrane protein | 3 | -0.152 | 1 | [1, 3, 5, 10] |
| Rv3804c | *fbpA* | Secreted antigen | 1 | -0.161 | 1 | [1-3, 6, 7] |
| Rv3806c | *-* | Possible conserved integral membrane protein | 3 | 0.705 | 7 | [1, 3, 10] |
| Rv3807c | *-* | Possible conserved transmembrane protein | 3 | 0.5 | 3 | [1] |
| Rv3808c | *glfT2* | Bifunctional udp-galactofuranosyl transferase | 3 | -0.286 | 0 | [1, 6] |
| Rv3809c | *glf* | UDP-galactopyranose mutase | 3 | -0.473 | 0 | [5, 6] |
| Rv3813c | *-* | Conserved hypothetical protein | 10 | 0.148 | 0 |  |
| Rv3818 | *-* | Hypothetical protein | 10 | -0.229 | 0 | [1, 3] |
| Rv3819 | *-* | Hypothetical protein | 10 | -0.166 | 0 | [1, 3] |
| Rv3825c | *pks2* | Possible polyketide synthase | 1 | 0 | 0 | [1, 6] |
| Rv3826 | *fadD23* | Possible fatty-acid-CoA ligase | 1 | 0.064 | 2 |  |
| Rv3835 | *-* | Possible conserved membrane protein | 3 | -0.318 | 1 | [2, 6] |
| Rv3838c | *pheA* | Possible prephenate dehydratase | 7 | 0.096 | 0 | [1] |
| Rv3841 | *bfrB* | Possible bacterioferritin | 7 | -0.277 | 0 | [1, 2, 5, 6, 9, 10] |
| Rv3842c | *glpQ1* | Possible glycerophosphoryl diester phosphodiesterase | 7 | -0.121 | 0 |  |
| Rv3846 | *sodA* | Superoxide dismutase | 0 | -0.206 | 0 | [1-3, 5-7, 10] |
| Rv3849 | *-* | Conserved hypothetical protein | 10 | -0.427 | 0 | [1, 2, 6, 7] |
| Rv3850 | *-* | Conserved hypothetical protein | 10 | -0.386 | 0 | [1, 6, 10] |
| Rv3851 | *-* | Possible membrane protein | 3 | 0.535 | 2 |  |
| Rv3852 | *hns* | Possible histone-like protein | 2 | -0.535 | 1 | [5, 10] |
| Rv3853 | *rraA* | Regulator of RNAse activity | 9 | 0.21 | 0 |  |
| Rv3858c | *gltD* | Possible NADH-dependent glutamate synthase | 7 | -0.297 | 0 | [1, 6] |
| Rv3863 | *-* | Hypothetical alanine rich protein | 10 | -0.216 | 0 | [6] |
| Rv3864 | *-* | Conserved hypothetical protein | 10 | 0.132 | 1 |  |
| Rv3865 | *-* | Conserved hypothetical protein | 10 | 0.183 | 0 | [3] |
| Rv3867 | *-* | Conserved hypothetical protein | 10 | -0.464 | 0 | [1] |
| Rv3869 | *-* | Possible conserved membrane protein | 3 | 0.03 | 1 | [9] |
| Rv3873 | *PPE68* | PPE family protein | 6 | -0.04 | 0 |  |
| Rv3874 | *esxB* | 10 kDa culture filtrate antigen | 3 | -0.668 | 0 | [1, 2, 6, 7] |
| Rv3875 | *esxA* | 6 kDa early secretory antigenic target | 3 | -0.256 | 0 | [1, 2, 7] |
| Rv3876 | *-* | Conserved hypothetical proline and alanine rich protein | 10 | -0.615 | 0 | [6] |
| Rv3877 | *-* | Possible conserved transmembrane protein | 3 | 0.67 | 11 |  |
| Rv3878 | *-* | Conserved hypothetical alanine rich protein | 10 | -0.063 | 0 |  |
| Rv3879c | *-* | Hypothetical alanine and proline rich protein | 10 | -0.234 | 0 | [6] |
| Rv3880c | *-* | Conserved hypothetical protein | 10 | -0.216 | 0 | [1, 3] |
| Rv3881c | *-* | Conserved hypothetical alanine and glycine rich protein | 10 | -0.483 | 0 | [2] |
| Rv3883c | *mycP1* | Membrane-anchored mycosin | 7 | 0.148 | 1 |  |
| Rv3888c | *-* | Possible conserved membrane protein | 3 | -0.309 | 0 | [1] |
| Rv3895c | *-* | Possible conserved membrane protein | 3 | 0.093 | 1 |  |
| Rv3909 | *-* | Conserved hypothetical protein | 10 | 0.036 | 0 | [6] |
| Rv3910 | *-* | Possible conserved transmembrane protein | 3 | 0.323 | 15 | [1, 6, 10] |
| Rv3913 | *trxB2* | Possible thioredoxin reductase | 7 | -0.092 | 0 | [7] |
| Rv3914 | *trxC* | Thioredoxin | 7 | 0.058 | 0 | [1, 2, 6, 7] |
| Rv3917c | *parB* | Possible chromosome partitioning protein | 3 | -0.23 | 0 | [1, 2, 6] |

Reference List

1. Gu SL, Chen J, Dobos KM, Bradbury EM, Belisle JT, and Chen X: **Comprehensive proteomic profiling of the membrane constituents of a *Mycobacterium tuberculosis* strain.** *Mol.Cell Proteomics* 2003, **2**:1284-1296.

2. Malen H, Berven FS, Fladmark KE, and Wiker HG: **Comprehensive analysis of exported proteins from *Mycobacterium tuberculosis* H37Rv**. *Proteomics* 2007, **7**:1702-1718.

3. Malen H, Berven FS, Softeland T, Arntzen MO, D'Santos CS, De Souza GA, and Wiker HG: **Membrane and membrane-associated proteins in Triton X-114 extracts of *Mycobacterium bovis* BCG identified using a combination of gel-based and gel-free fractionation strategies**. *Proteomics.* 2008*,* **8:** 1859-1870.

4. Mattow J, Schaible UE, Schmidt F, Hagens K, Siejak F, Brestrich G, Haeselbarth G, Muller EC, Jungblut PR, and Kaufmann S H: **Comparative proteome analysis of culture supernatant proteins from virulent Mycobacterium tuberculosis H37Rv and attenuated M. bovis BCG Copenhagen.** *Electrophoresis* 2003, **24**:3405-3420.

5. Mattow J, Siejak F, Hagens K, Schmidt F, Koehler C, Treumann A, Schaible UE, and Kaufmann SH: **An improved strategy for selective and efficient enrichment of integral plasma membrane proteins of mycobacteria**. *Proteomics.* 2007*,* **7:** 1687-1701.

6. Mawuenyega KG, Forst CV, Dobos KM, Belisle JT, Chen J, Bradbury EM, Bradbury AR, and Chen X: ***Mycobacterium tuberculosis* functional network analysis by global subcellular protein profiling**. *Mol.Biol.Cell* 2005*,* **16:** 396-404.

7. Rosenkrands I, King A, Weldingh K, Moniatte M, Moertz E, and Andersen P: **Towards the proteome of *Mycobacterium tuberculosis***. *Electrophoresis* 2000*,* **21:** 3740-3756.

8. Schmidt F, Donahoe S, Hagens K, Mattow J, Schaible UE, Kaufmann SH, Aebersold R, and Jungblut PR: **Complementary analysis of the *Mycobacterium tuberculosis* proteome by two-dimensional electrophoresis and isotope-coded affinity tag technology**. *Mol.Cell Proteomics* 2004. **3**:24-42.

9. Sinha S, Kosalai K, Arora S, Namane A, Sharma P, Gaikwad AN, Brodin P, and Cole ST: **Immunogenic membrane-associated proteins of *Mycobacterium tuberculosis* revealed by proteomics**. *Microbiology* 2005*,* **151:** 2411-2419.

10. Xiong Y, Chalmers MJ, Gao FP, Cross TA, and Marshall AG: **Identification of *Mycobacterium tuberculosis* H37Rv integral membrane proteins by one-dimensional gel electrophoresis and liquid chromatography electrospray ionization tandem mass spectrometry**. *J.Proteome.Res.* 2005*,* **4:** 855-861.
